# Potential impact and cost-effectiveness of oral HIV pre-exposure prophylaxis for men who have sex with men in Cotonou, Benin: a mathematical modelling study

**DOI:** 10.1016/S2214-109X(25)00098-1

**Published:** 2025-06

**Authors:** Trystan Leng, Léon Kessou, Jesse Heitner, Fernand A Guédou, Luc Béhanzin, Marius Olodo, Souleymane Diabaté, Romain Silhol, Dobromir Dimitrov, Peter Vickerman, Michel Alary, Marie-Claude Boily, Kate M Mitchell

**Affiliations:** UK Medical Research Council Centre for Global Infectious Disease Analysis, School of Public Health, Imperial College London, London, UK; Service de Consultance et Expertise Nouvelle en Afrique, Cotonou, Benin; Massachusetts General Hospital, Boston, MA, USA; OPSDC-ONG, Dispensaire des IST, Centre de Santé Communal de Cotonou-1, Cotonou, Benin; École Nationale de Formation des Techniciens Supérieurs en Santé Publique et en Surveillance Épidémiologique, University of Parakou, Parakou, Benin; OPSDC-ONG, Dispensaire des IST, Centre de Santé Communal de Cotonou-1, Cotonou, Benin; Centre de recherche du CHU de Québec-Université Laval, Québec, QC, Canada; UK Medical Research Council Centre for Global Infectious Disease Analysis, School of Public Health, Imperial College London, London, UK; Fred Hutchinson Cancer Center, Seattle, WA, USA; Population Health Science, Bristol Medical School, University of Bristol, Bristol, UK; Centre de recherche du CHU de Québec-Université Laval, Québec, QC, Canada; UK Medical Research Council Centre for Global Infectious Disease Analysis, School of Public Health, Imperial College London, London, UK; UK Medical Research Council Centre for Global Infectious Disease Analysis, School of Public Health, Imperial College London, London, UK; Department of Nursing and Community Health, Glasgow Caledonian University London, London, UK

## Abstract

**Background:**

Oral HIV pre-exposure prophylaxis (PrEP) can effectively reduce HIV incidence. A 2020–21 demonstration project assessed the feasibility and health outcomes of offering oral PrEP to men who have sex with men (MSM) in Cotonou, Benin. We evaluated the epidemiological impact and cost-effectiveness of this project and the potential scale-up of oral HIV PrEP for MSM in Cotonou.

**Methods:**

We calibrated an HIV transmission-dynamic model structured by age and risk within a Bayesian framework to MSM-specific HIV prevalence and treatment data, parameterised with project behavioural and cost (including PrEP drug, implementation, and HIV care costs) data. We estimated the impact and cost-effectiveness of the 2020–21 Cotonou demonstration project (PrEP coverage, 5–10% of all MSM who are not living with HIV in Grand Cotonou; and adherence, 13–21% taking at least four of seven required doses [ie, at least four doses per week for daily users and at least four of seven expected doses given reported sexual activity for on-demand users]) and of its potential scale-up over 5 years (from 2022 to 2027), reaching 30% coverage of MSM in Grand Cotonou and with demonstration project adherence levels. We additionally modelled ideal PrEP adherence (100% taking at least four of seven required doses). We estimated the percentage of cumulative new HIV infections averted among participating MSM over 1 year and among all MSM in Grand Cotonou and their female partners over 20 years, and cost-effectiveness as cost per disability-adjusted life-year (DALY) averted over 20 years. Costs and DALYs were discounted 3% annually.

**Findings:**

We found that the demonstration project averted an estimated 21·5% (95% uncertainty interval 16·6 to 26·2) of HIV infections among participants over 1 year. With ideal adherence, cases that would be averted increased to 95·2% (90·8 to 98·8). A 5-year PrEP scale-up could avert 3·2% (1·6 to 4·8) of HIV infections among all MSM and female partners over 20 years, at US$388 (36 to 2792) per DALY averted. With ideal adherence, this decreased to −$28 (−126 to 589) per DALY averted.

**Interpretation:**

Low adherence to PrEP restricted the impact of the demonstration project. At 30% coverage among MSM by 2027, PrEP scale-up would be cost-effective at a $1225 threshold with 86·6% probability, and it could be more cost-effective if high adherence could be reached without substantially increasing costs.

**Funding:**

Canadian Institutes of Health Research and US National Institutes of Health.

## Introduction

Key populations, including men who have sex with men (MSM) and female sex workers, have a disproportionate burden of HIV infection in west Africa.^1,2^ In Benin, HIV prevalence among MSM was 8% in 2022, compared with 0·5% among the overall population of men.^3^ Accordingly, ensuring that key populations, including MSM, can access a combination of prevention options is part of Benin’s national HIV prevention strategy.^2^ In several countries in west Africa,^3^ oral HIV pre-exposure prophylaxis (PrEP) is now available.

Oral PrEP has been shown to reduce HIV incidence, including among MSM, when used adequately.^4,5^ Four or more doses a week of a daily oral PrEP regimen of tenofovir disoproxil fumarate and emtricitabine (TDF/FTC) can reduce HIV risk among MSM by 90–100%.^6^ On-demand PrEP, where individuals take TDF/FTC over periods of sexual activity, can also reduce HIV risk by a similar amount.^7^ However, high adherence to oral PrEP regimens is required for such a reduction.^5^ Although long-acting injectable PrEP modalities are likely to become available in the next few years, it is important to evaluate the impact and cost-effectiveness of PrEP interventions that can be implemented before long-acting PrEP becomes available.

Previous modelling suggests that oral PrEP can reduce HIV incidence, but it might not always be cost-effective.^8,9^ Because cost-effectiveness depends on pre-intervention HIV incidence and the reduction in HIV incidence achieved, context-specific modelling capturing data among the relevant populations is required to generate conclusive findings.^9^ To our knowledge, no previous study has modelled the impact of PrEP for MSM in west Africa. Two demonstration projects in west Africa have explored the feasibility and health outcomes associated with oral PrEP for MSM: a demonstration project in Cotonou, Benin from 2020 to 2021,^10^ and a multicountry project (CohMSM-PrEP) from 2017 to 2021.^11^ The Cotonou demonstration project collected data on participant retention, regimen choice, seroconversion, sexual behaviour, and concentrations of tenofovir diphosphate in dried blood spots (to objectively evaluate adherence to PrEP).

In this study, these data were used to parametrise and calibrate a mathematical HIV transmission-dynamic model to evaluate the impact and cost-effectiveness of oral PrEP offered to MSM in Cotonou. We evaluated the 20-year impact of the 1-year MSM PrEP demonstration project in Cotonou in 2020–21, and of scaling up PrEP to the wider MSM population of Cotonou and Abomey-Calavi from 2022 to 2027.

## Methods

### Model structure

We used a deterministic compartmental model of HIV transmission among MSM aged 18–50 years in Cotonou and Abomey-Calavi (Grand Cotonou), Benin. The model was adapted from a US model^12^ through redesign, parametrisation, and calibration to reflect the local Benin context (appendix 2 p 4). The model represented an MSM population stratified by age (18–24, 25–50, and ≥50 years), behavioural risk, HIV testing, PrEP use, HIV infection, HIV care stage, and CD4 cell count, with MSM entering, transitioning through, and exiting the population as they age. We modelled MSM aged 50 years and older to track life-years lived with HIV and HIV-related deaths, but did not model their sexual activity due to insufficient available behavioural data. Motivated by the high proportion (57%) of MSM in the Cotonou demonstration project identifying as bisexual,^10^ we estimated HIV transmission to female partners of MSM using a risk-equation approach.^13^

HIV transmission between MSM occurred in non-commercial or commercial partnerships. HIV transmission from MSM to female partners occurred in stable or casual partnerships. Sex act frequency and condom usage varied by partnership type. Per capita rates of HIV acquisition among MSM, and of HIV transmission to female partners, depended on partnership change rates, number of sex acts per partnership (per non-commercial or commercial partnership with MSM partners, and per stable or casual partnership with female partners), transmission probabilities per act, proportion of condom-protected acts, HIV prevalence, infection stage, viral suppression levels, PrEP use, and PrEP effectiveness among MSM. Model details, schematics, and equations are given in appendix 2 (pp 5–26).

### Modelling PrEP

Daily and on-demand PrEP regimens were modelled to reflect the demonstration project, including regimen switching and discontinuation. We calculated the effectiveness of each regimen against HIV acquisition as a weighted average over three PrEP adherence strata, using strata-specific efficacies from PrEP trials (fully adherent 90–100%, partly adherent 56–96%, and non-adherent 0%^6^), with proportions of PrEP users in each stratum informed by demonstration project biological data. Full adherence was defined as having tenofovir diphosphate levels consistent with having taken four or more of the seven required TDF/FTC doses, partial adherence as having taken two to three doses, and nonadherence as having taken one dose or fewer. Required doses for adherence differs between regimens. As on-demand users follow a 2–1–1 dosing schedule, required dosage depends on sexual activity levels (appendix 2 pp 24–26).

### Model parameters and data sources

Demographic parameters were drawn from UN World Population Prospects estimates for Benin.^14^ Behavioural parameters were obtained from demonstration project^10^ survey responses and biobehavioural surveys of MSM in Grand Cotonou^15,16^ (appendix 2 p 10). The proportion of MSM at high risk was derived from the proportion of demonstration project participants who reported inconsistent condom use or a partner living with HIV in the previous 6 months, or who tested positive for chlamydia or gonorrhoea at recruitment. HIV transmission and progression parameters were informed from published literature for sub-Saharan Africa. Data-informed model parameter priors are detailed in the table and appendix 2 (pp 10–17). Modelled trends in partnerships, condom use, and testing are shown in appendix 2 (pp 42–43).

### Model calibration

The model was calibrated using a Markov chain Monte Carlo approach to data from biobehavioural surveys of MSM in Grand Cotonou on population size, age distribution, overall HIV prevalence (our main scenario) or age-stratified prevalence (sensitivity analysis), antiretroviral therapy (ART) coverage, viral suppression, and the proportion of MSM aged 25–50 years who had never tested for HIV (table, appendix 2 p 33). We performed 1500 000 steps of a Metropolis–Hastings algorithm, discarded the first 200 000 steps, and thinned the remaining set to 1000 parameter sets. Model fits were cross-validated against observed demonstration project incidence. Markov chain Monte Carlo algorithm details, diagnostics, and posteriors are given in appendix 2 (pp 33–41).

### PrEP scenarios

We modelled two PrEP intervention scenarios. First, a 1-year demonstration project, in which 163 MSM were initiated onto daily PrEP and 41 onto on-demand PrEP for 1 year from 2020 (5–10% of HIV-negative MSM in Grand Cotonou). Second, a 5-year PrEP scale-up, in which from 2022, MSM not living with HIV were routinely offered the choice of daily or on-demand PrEP upon testing for HIV, and we calibrated the probability of accepting PrEP to obtain scenarios of 10%, 30%, and 50% coverage among MSM not living with HIV by 2027 (appendix 2 p 47). In both scenarios, all participants stopped taking oral PrEP once it was no longer offered after 1 year in the first scenario and 5 years in the second scenario. Scenarios were considered independently and were compared with a counterfactual scenario without PrEP.

### Model outcomes

We measured impact as the cumulative number of new HIV infections averted among all MSM aged 18–50 years and their female partners over 20 years compared with the counterfactual without PrEP. For the demonstration project, we also estimated the number of new HIV infections averted over 1 year among project participants.

For each scenario, we estimated the disability-adjusted life-years (DALYs) averted over 20 years by comparing the number of years lived with disease and years of life lost (YLL) due to HIV infection with the counterfactual without PrEP, using 2019 Global Burden of Disease weights.^18^ YLL were estimated using Benin-specific life expectancies at the midpoint of each age group, with YLL counted immediately upon death. DALYs for female partners were estimated by multiplying the cumulative number of new HIV infections averted among female partners with DALYs per infection averted among MSM. DALYs were discounted at 3% annually.

For each PrEP scenario, we calculated the incremental cost effectiveness ratio (ICER), ie, the cost per DALY averted compared with the counterfactual without PrEP, over 20 years. We compared the ICER with willingness-to-pay thresholds of 1 × Benin gross domestic product (GDP) per capita (US$1225), the previous WHO threshold for very cost-effective interventions,^19^ and 0·29 × GDP per capita ($355), a Benin-specific threshold based on health opportunity costs analysis.^20^

Model outcomes are expressed as median values and 95% uncertainty intervals based on 1000 runs. Output equations are detailed in appendix 2 (p 31).

### Cost assumptions

We used a bottom-up costing approach to estimate costs from the perspective of the Benin Ministry of Health. We included PrEP drug and implementation costs (from the demonstration project^10^), ART costs (using ART costs among female sex workers in Cotonou^17^), and costs associated with untreated HIV infection (eg, treating opportunistic infections). PrEP scale-up costs were adjusted to reflect likely changes in PrEP costs and service provision at scale-up (Carin Ahouada, Plan International Bénin, personal communication; appendix 2 p 28). The modelled lifetime cost of HIV care was sourced from a review of costs from lower-middle-income countries ($3619; appendix 2 pp 27–29).^21^

Demonstration project costs in Communauté Financière Africaine francs were converted to US$ using an exchange rate from April 28, 2021 (the project midpoint). ART costs, lifetime HIV care costs, and cost-effectiveness thresholds were adjusted to US$ at 2021 values using the World Bank Benin inflation index.^22^ Costs were discounted at 3% annually (table, appendix 2 p 17). All costs are presented in US$ at 2021 values.

### Sensitivity analysis

We considered a range of scenarios assessing the sensitivity of our main results to modelling assumptions, and to PrEP adherence and coverage (appendix 2 p 32). We defined two alternative adherence scenarios: an optimistic scenario, from an alternative interpretation of demonstration project biological adherence data in which we classified those with low but detectable levels of tenofovir diphosphate as partly adherent and classified that on-demand users who reported no sexual activity in the past month had full adherence irrespective of their tenofovir diphosphate levels (appendix 2 p 26); and an ideal scenario, where all PrEP users take four or more of the seven required doses. Ideal adherence in the demonstration project was modelled by also assuming that no participants discontinued PrEP over 1 year. We assumed no extra costs for improving adherence.

Biobehavioural surveys report higher HIV prevalence among MSM aged 18–24 years than MSM aged 25–50 years in 2017^15^ and 2022.^16^ This sustained pattern is difficult to reconcile for HIV, so calibrating the model to age-specific prevalence forced incidence to increase considerably after 2020. However, given the uncertainty in the data and HIV epidemiological context, we believe there is insufficient evidence to strongly conclude that HIV incidence will increase. Therefore, for the main analysis, we fitted the model to overall HIV prevalence over time, which resulted in a wider range of plausible future epidemic trajectories, including constant and decreasing incidence. We evaluated the sensitivity of results to future HIV incidence trends by recalibrating the model to age-specific HIV prevalence estimates.

We also assessed the sensitivity of results to the inclusion or exclusion of HIV infections among female partners, risk prioritisation (offering PrEP to MSM at high risk only), implementation costs, lifetime HIV care costs, discount rates, DALY calculation method (considering an alternative calculation whereby YLL after the intervention evaluation period are not included^23^), and intervention evaluation period (50 *vs* 20 years). We considered these sensitivity scenarios for a 5-year scale-up, for a 20-year scale-up, and for the demonstration project.

### Threshold analysis

We considered the relative change in PrEP costs during PrEP scale-up required to reach an ICER below a 1 × GDP per capita threshold.

### Ethics statement

Ethical approval for this study was granted by the ethics committee of the Centre Hospitalier Universitaire de Québec–Université Laval in Québec, Canada (2020–5106) and the Benin National Ethics Committee for Health Research (N°014/MS/DC/SGM/DRFMT/CNERS/SA-Avis).

### Role of the funding source

The funders had no role in study design, data collection, data analysis, data interpretation, or writing of the report.

## Results

**Figure 1:**
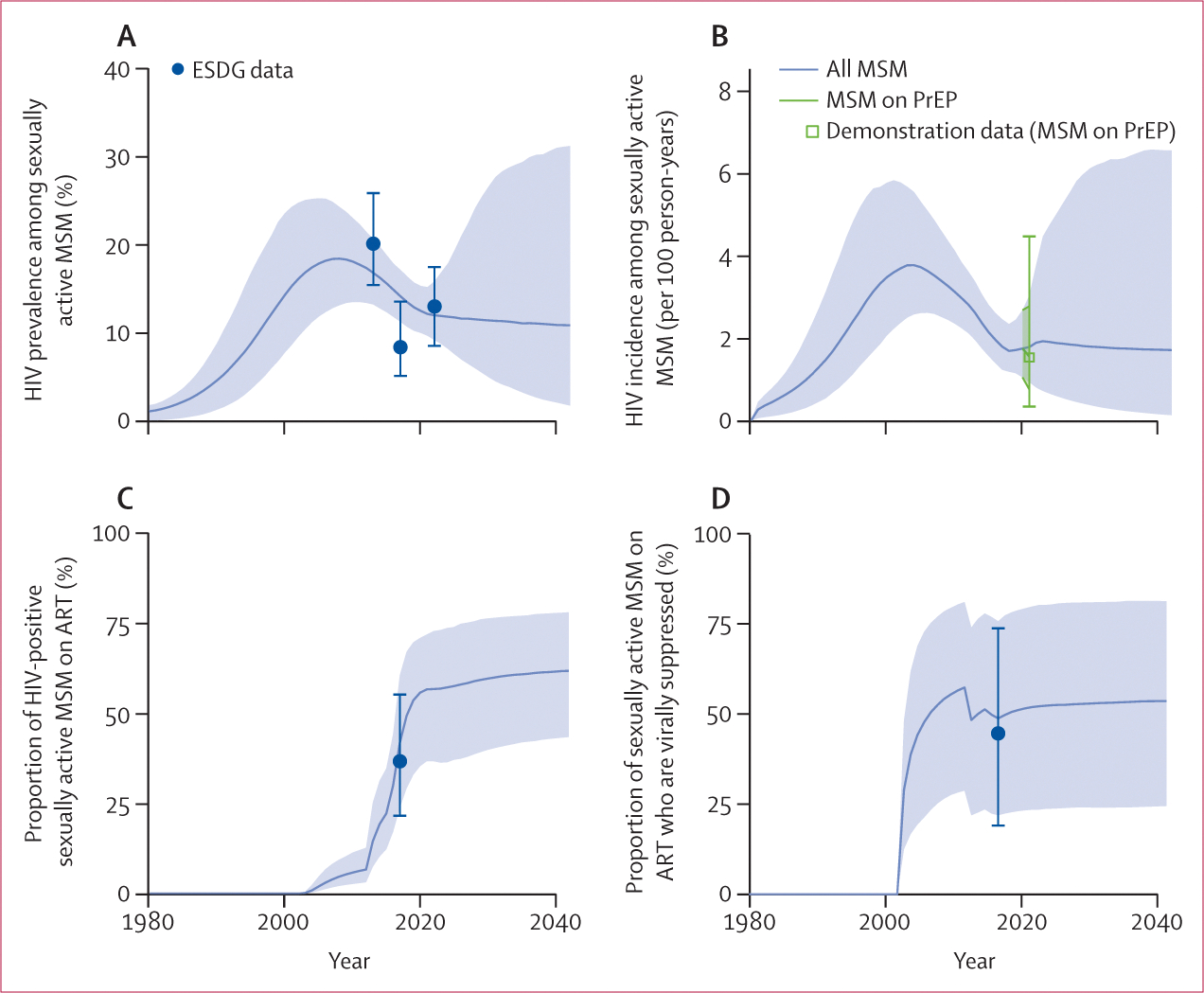
Model fits to key HIV and treatment outcomes Overall HIV prevalence among sexually active MSM (A). HIV incidence per 100 person years among all MSM (grey) and among MSM on PrEP (green) (B). Proportion of HIV-positive MSM on ART (C). Proportion of HIV-positive MSM on ART who are virally suppressed (D). Circle markers represent data from ESDG biobehavioural surveys of MSM in Benin, square markers represent data from the 2020–21 MSM PrEP demonstration project in Cotonou, and crossbars show 95% CI estimates from data. Solid lines and shaded ribbons represent median model predictions and 95% uncertainty intervals from 1000 posterior parameter sets. ART=antiretroviral therapy. ESDG=Enquete de Surveillance de Deuxieme Generation. MSM=men who have sex with men. PrEP=pre-exposure prophylaxis.

The calibrated model reflected the epidemiological HIV and treatment data available for MSM in Cotonou, Benin well, as well as their uncertainty, which resulted in a wide range of plausible future epidemic trajectories (figure 1; appendix 2 p 43). In our main calibration, calibrated to observed overall HIV prevalence among MSM in Grand Cotonou, modelled median HIV prevalence (12·2% in 2022) and incidence (1·9 per 100 person-years in 2022) remained relatively constant from 2022 onwards (figure 1). However, these values increased over time in the additional scenario calibrated to age-specific HIV prevalence data (appendix 2 pp 44–45).

**Figure 2:**
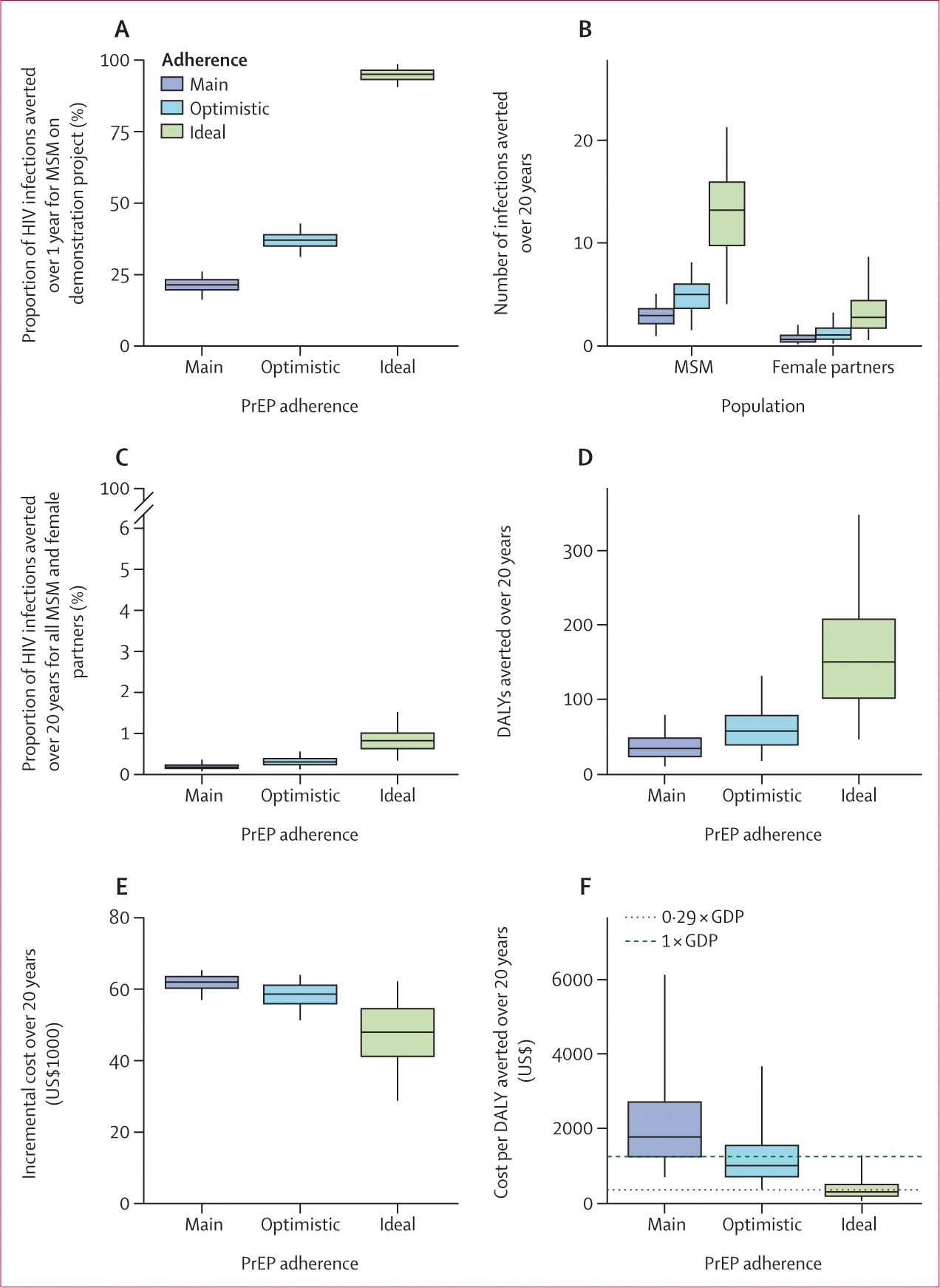
Impact and cost-effectiveness of the 1-year PrEP demonstration project Proportion of HIV infections averted over 1 year among 204 MSM who participated in a 1-year demonstration project (100% of the participants on PrEP) (A). Number of HIV infections averted over 20 years among MSM in Grand Cotonou (left) and among female partners of MSM (right); 5–10% of all uninfected MSM are on PrEP (B). Proportion of HIV infections averted over 20 years among all MSM in Grand Cotonou and their female partners (C). DALYs averted over 20 years (D). Incremental costs (compared with a counterfactual without PrEP) over 20 years (E). Cost per DALY averted over 20 years (F). Dashed line indicates 1 × GDP per capita cost-effectiveness threshold and dotted line indicates 0·29 × GDP per capita cost-effectiveness threshold. Three adherence scenarios are considered (main, optimistic, and ideal; PrEP regimen effectiveness for each adherence scenario is described in the table). Box plots depict model predictions generated from 1000 posterior parameter sets. Whiskers represent the 2·5th and 97·5th percentiles, boxes indicate the 25th and 75th percentiles, and the central line represents the median value. DALY=disability-adjusted life-year. GDP=gross domestic product. MSM=men who have sex with men. PrEP=pre-exposure prophylaxis.

Our results suggest that the demonstration project averted 21·5% (95% uncertainty interval 16·6–26·2) participant HIV infections over 1 year in our main adherence scenario, 37·0% (31·2–42·9) in our optimistic scenario, and 95·2% (90·8–98·8) in our ideal scenario (figure 2A).

The 1-year project (which enrolled 204 [5–10%] of all MSM not living with HIV in Grand Cotonou) was estimated to avert a total of three (95% uncertainty interval 1–5) new HIV infections among MSM and one (0–2) among their female partners over 20 years (figure 2B). Together, this amounted to 0·2% (0·1–0·4) of all HIV infections averted among all MSM and female partners over 20 years in Grand Cotonou (figure 2C), and it did not noticeably affect long-term HIV incidence (appendix 2 p 46).

The demonstration project averted an estimated 34·0 (95% uncertainty interval 10·5–79·6) DALYs at a net cost of $61 688 (56 712–64 973) over 20 years (figure 2D, E). The project ICER over 20 years decreased from $1772 (680–6156) for our main adherence scenario, to $994 (352–3679) for our optimistic scenario, to $285 (44–1280) for our ideal scenario (figure 2F). The ICER fell below the 1 × GDP (0·29 × GDP) threshold in only 24·2% (0%) of model runs under our main adherence scenario, compared with 96·9% (61·2%) assuming ideal adherence. Modelled PrEP regimen choice reflected the trend observed in the demonstration project (appendix 2 p 46). A costs breakdown is shown in appendix 2 (p 47).

**Figure 3:**
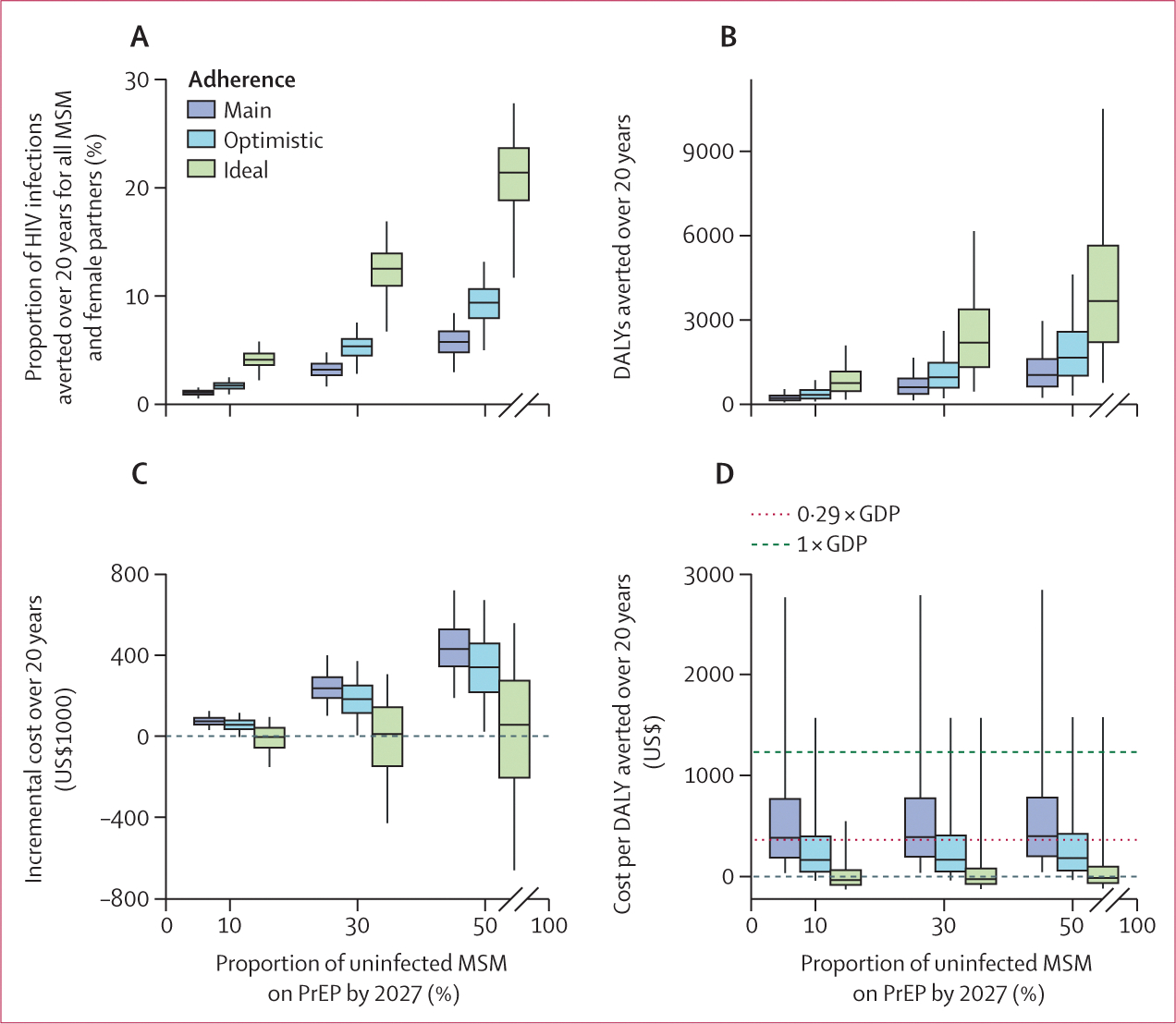
Impact and cost-effectiveness of a 5-year PrEP scale-up to MSM in Grand Cotonou for 20 years from 2022, at 10%, 30%, and 50% coverage by 2027 Proportion of HIV infections averted over 20 years among all MSM in Grand Cotonou and their female partners (A). DALYs averted over 20 years (B). Incremental costs (costs of intervention minus costs of a counterfactual scenario without PrEP) over 20 years (C). Cost per DALY averted over 20 years (D). Three adherence scenarios are considered (main, optimistic, and ideal; PrEP regimen effectiveness for each adherence scenario is described in the table). Dashed line indicates 1 × GDP per capita cost-effectiveness threshold and dotted line indicates 0·29 × GDP per capita cost-effectiveness threshold. Box plots depict model predictions generated from 1000 posterior parameter sets. Whiskers represent the 2·5th and 97·5th percentiles, boxes indicate the 25th and 75th percentiles, and the central line represents the median value. DALY=disability-adjusted life-year. GDP=gross domestic product. MSM=men who have sex with men. PrEP=pre-exposure prophylaxis.

A 5-year PrEP scale-up reaching 30% PrEP coverage of HIV-uninfected MSM in Grand Cotonou by 2027 could avert 3·2% (95% uncertainty interval 1·6–4·8) of all HIV infections among MSM and their female partners over 20 years under our main adherence scenario, 5·3% (2·8–7·5) under our optimistic scenario, and 12·5% (6·7–16·9) under our ideal scenario (figure 3A). Impact was 1·8 (1·7–1·9) times higher at 50% than at 30% coverage (figure 3A). Prevalence and incidence trends, and breakdowns of infections averted and costs, are shown in appendix 2 (p 48).

**Figure 4:**
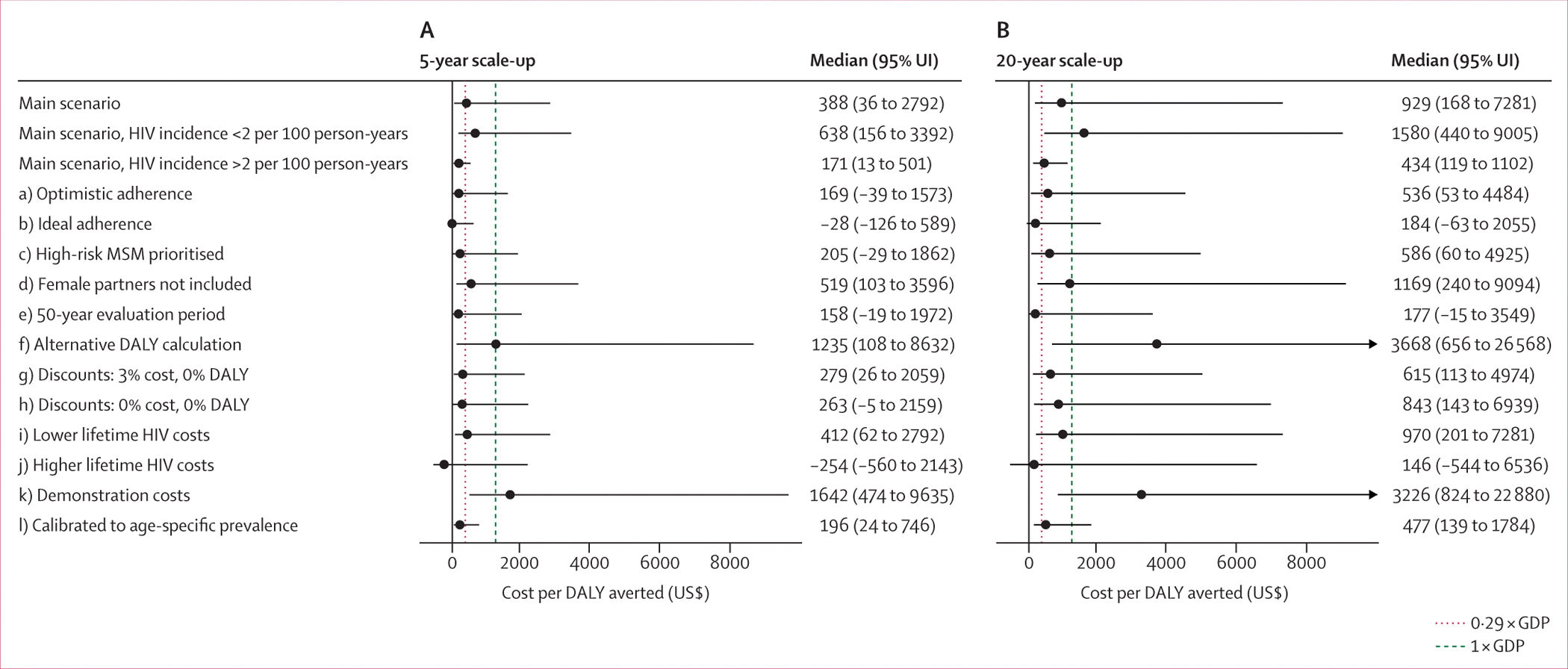
Sensitivity analysis of the cost-effectiveness of PrEP scale-up from 2022, with 30% coverage by 2027 Cost per DALY averted from a 5-year (A) and 20-year (B) PrEP scale-up to MSM in Grand Cotonou under our main scenario (overall and stratified by HIV incidence in 2021), alongside a range of adherence (a–b), distribution (c), DALY calculation (d–h), costing (i–k), and calibration (l) sensitivity scenarios (appendix 2 p 32). In all scenarios, model predictions are generated from 1000 posterior parameter sets. Points indicate median values and lines indicate 95% UIs. Dashed line indicates 1 × GDP per capita cost-effectiveness threshold and dotted line indicates 0·29 × GDP per capita cost-effectiveness threshold. Arrows are present for scenarios in which the upper 95% UI exceeded US$10 000 per DALY averted. DALY=disability-adjusted life-year. GDP=gross domestic product. MSM=men who have sex with men. PrEP=pre-exposure prophylaxis. UI=uncertainty interval.

Over 20 years, a 5-year scale-up with 30% PrEP coverage by 2027 could avert 570 DALYs (95% uncertainty interval 111 to 1628; figure 3B) at a net cost of $237 916 (101 861 to 400 497; figure 3C). The ICER of PrEP scale-up at 30% coverage was $388 (36 to 2792) under our main adherence scenario, $169 (−39 to 1573) under our optimistic scenario, and −$28 (−126 to 589) under our ideal scenario (figure 3D). Cost-effectiveness estimates were similar across different coverage levels (figure 3D, appendix 2 p 49). The ICER fell below the 1 × GDP (0·29 × GDP) threshold in 86·6% (46·1%) of model runs under our main adherence scenario, 95·7% (71·5%) under our optimistic scenario, and 99·6% (94·3%) under our ideal scenario. Estimated ICERs were uncertain and dependent on HIV incidence both at baseline and after 20 years in the absence of PrEP (appendix 2 p 50). For example, in our main adherence scenario, at 30% coverage, the ICER fell below a 0·29 × GDP threshold ($171 [13 to 501]) when HIV incidence exceeded 2 per 100 person-years (figure 4).

The median ICER of the 5-year PrEP scale-up was closer to the 0·29 × GDP than the 1 × GDP threshold for most sensitivity analysis scenarios, and was lower for eight scenarios: optimistic adherence, ideal adherence, risk prioritisation, a 50-year evaluation period, DALYs not being discounted, DALYs and costs not being discounted, higher lifetime HIV care costs, and alternative calibration to age-specific prevalence. These ICER values were lower because more DALYs were averted or lower incremental costs were incurred than in the main scenario (appendix 2 pp 51–52). For ideal adherence and higher lifetime HIV care costs, PrEP scale-up was cost-saving in most model runs. The median ICER of a 5-year scale-up was above a 1 × GDP threshold under two scenarios: using an alternative DALY calculation (because fewer DALYs were averted), and using demonstration project costs (because higher costs were incurred).

The median ICER of a 20-year scale-up of our main cost-effectiveness scenario fell just below the 1 × GDP threshold. Because we used a 20-year intervention evaluation period, the health benefits of offering PrEP towards the end of the 20-year scale-up are not fully captured, resulting in a higher ICER than a 5-year PrEP scale-up. The median ICER fell below a 0·29 × GDP threshold for scenarios of ideal adherence, 50-year evaluation period, and higher lifetime HIV costs. Notably, similar ICERs were obtained for a 5-year and 20-year scale-up when evaluated over 50 years.

For the 1-year demonstration project, only ideal adherence resulted in a median ICER below 0·29 × GDP (appendix 2 53–54).

Combined initiation and ongoing PrEP costs would have to be under 2·5 times their PrEP scale-up value (95% uncertainty 0·5–7·1) under our main adherence scenario, 4·1 times (0·8–11·3) under our optimistic scenario, and 9·4 times (1·8–26·1) under our ideal scenario to keep the ICER of PrEP scale-up below 1 × GDP (table; appendix 2 p 55).

## Discussion

Our modelling suggests that the Cotonou PrEP demonstration project might have averted around one-fifth of HIV infections among participating MSM over 1 year. However, the project impact was hindered by low adherence levels observed among participants (13–21% taking at least four of seven prescribed doses). A 5-year PrEP scale-up reaching 30% coverage by 2027 could avert 2–5% of infections among MSM and their female partners over 20 years. Based on the median ICER, PrEP scale-up (at 10%, 30%, and 50% coverage by 2027) was cost-effective at a 1 × GDP ($1225) threshold, but the demonstration project was not. At a 0·29 × GDP ($355) threshold, PrEP scale-up was cost-effective in 46·2% of model runs under our main adherence scenario.

The potential impact of PrEP scale-up depends on coverage reached, which will partly depend on PrEP acceptability. In an acceptability study in Benin, 90% of surveyed MSM, once well informed, indicated that they would use PrEP.^24^ This figure is higher than the modelled PrEP acceptance probability (16–27%) required to reach 50% coverage when PrEP is offered at HIV testing. Higher coverage and greater population-level impact might be seen if more MSM can be enrolled onto PrEP by, for example, ensuring free PrEP access and using existing community networks for distribution.^24^ On the other hand, higher PrEP discontinuation rates than those observed during the demonstration project (as observed at scale-up in Cameroon^25^) would reduce coverage and the population-level impact of PrEP scale-up.

PrEP adherence was a key determinant of cost-effectiveness in this study. In an ideal—arguably unrealistic—adherence scenario (at no extra cost), both demonstration project and PrEP scale-up were cost-effective at both thresholds considered. Our threshold analysis suggested that even with substantially higher PrEP costs, PrEP could remain cost-effective (at a 1 × GDP threshold) with high adherence levels, highlighting the importance of understanding how to improve adherence. Our model assumed that non-adherent PrEP users receive no benefit, but assuming partial protection would increase the cost-effectiveness of PrEP.

Our results are similar to those of other PrEP modelling studies for key populations in west and central Africa. A study modelling oral PrEP for female sex workers in Cotonou also reported very minor long-term effects of a 2-year demonstration project in female sex workers due to low observed adherence. In that study, the PrEP scale-up scenarios considered were unlikely to be cost-effective, mainly due to relatively low HIV incidence^17^ (0–3 per 100 person-years in 2015^26^). A model of PrEP for MSM in Yaoundé and Douala, Cameroon (which are settings of higher prevalence than Cotonou) assumed higher PrEP effectiveness than this study, but showed similar changes in prevalence over 5 years.^27^

Models that represent population-specific context help refine our understanding of the effect of HIV interventions. Through calibration to MSM-specific and context-specific data, our model reflected plausible HIV trends among MSM in Grand Cotonou, with the wide range of modelled epidemiological trajectories reflecting the uncertainty in underlying data. Our PrEP impact estimates could apply to other settings with similar behavioural characteristics and adherence levels among MSM PrEP users. Consistent with previous studies,^8^ we observed a strong positive association between pre-intervention HIV incidence and PrEP cost-effectiveness. Although our model is cross-validated against demonstration project HIV incidence levels, higher levels were observed in a 2016–18 study among MSM in Benin.^28^ If HIV incidence rates were higher than those predicted by our model, PrEP might be more cost-effective than our results suggest.

Although the median ICER of PrEP scale-up did not fall below a 0·29 × GDP threshold under our main scenario evaluated over 20 years, the median ICER of both a 5-year and a 20-year PrEP scale-up fell below this threshold when evaluated over 50 years because more DALYs were averted and greater savings in HIV care costs were accrued, highlighting the large effect the intervention evaluation period can have on PrEP cost-effectiveness estimates.^9^ In contrast, PrEP scale-up was approximately three times less cost-effective when using an alternative DALY calculation,^23^ whereby YLL after the intervention evaluation period were not included.

This study focused on oral PrEP, the only modality currently available in Benin. Our study shows that PrEP scale-up is likely to be cost-effective (at a 1 × GDP threshold) if offered for a fixed period before long-acting injectable PrEP becomes available. It will be important for future studies to evaluate the relative impact and cost-effectiveness of long-acting PrEP compared with oral PrEP. Given the low PrEP adherence observed during the demonstration project, long-acting PrEP has the potential to improve impact. However, uncertainty remains over the costs of injectable modalities, which will be required to evaluate cost-effectiveness.

Our study has several limitations. First, behavioural parameters were based on survey responses of MSM recruited via respondent-driven sampling, which, despite being the gold standard to sample key populations,^29^ might not be fully representative of the wider MSM population. In particular, older MSM were likely to have been undersampled. Although homosexuality is legal in Benin, MSM face discrimination and stigmatisation, which could affect engagement with surveys differentially by age. Recall bias might also affect response accuracy. These factors could partly explain why HIV prevalence was higher among MSM aged 18–24 years compared with those aged 25–50 years in biobehavioural surveys—a pattern difficult to reconcile in our model. Future surveys could elucidate these trends. Second, our model does not capture factors such as underlying health conditions or education or income levels, which might affect HIV incidence, mortality, access to HIV care, and PrEP use. Our model also uses broad age categories. Given the size of the demonstration project, it was not feasible to obtain informative estimates of parameters by multiple factors or narrower age bands. Future studies using different stratifications could reveal heterogeneity in HIV prevalence and allow additional scenarios to be explored. Third, although we predominantly used Benin-specific cost data, lifetime HIV care costs were informed by an estimate for lower-middle-income countries.^21^ PrEP scale-up was cost-effective at a 0·29 × GDP threshold when higher lifetime costs were assumed. Therefore, Benin-specific estimates of HIV-related care costs would be valuable. Finally, our model did not distinguish between HIV-1 and HIV-2 due to insufficient available data. HIV-2 is endemic at low levels in west Africa,^30^ and its reduced transmissibility and slower disease progression might result in differences in treatment costs but would be unlikely to materially change cost-effectiveness results (which are largely determined by incidence).

In summary, our modelling suggests that low adherence to oral PrEP hampered the impact of the 1-year demonstration project. However, despite low adherence, a 5-year PrEP scale-up from 2022 would be expected to be cost-effective at a 1 × GDP cost-effectiveness threshold, and such a scale-up could fall below a 0·29 × GDP threshold if high adherence can be ensured without substantially increasing implementation costs. Our results highlight that oral PrEP can be a useful HIV intervention for MSM in Cotonou, particularly in the period before long-acting injectable PrEP is introduced.

## Supplementary Material

Supplementary Appendix 1

Supplementary Appendix 2

Supplementary Appendix 3

## Figures and Tables

**Table:** Key epidemiological and cost model parameters and fitting outcomes for the main analysis

	1-year demonstration project range	Source

**Demography**		
Annual population growth rate, %	2·3–4·3%	UN World Population Prospects^14^
New MSM aged 18–24 years, %	93–98%	ESDG MSM surveys (Grand Cotonou), 2017 and 2022^15,16^
New MSM at high risk, %	60–73%	Cotonou MSM PrEP demonstration project, 2020–21^10^
**Average number of male sexual partners per 6 months in 2022**		
MSM aged 18–24 years	1·9–5·5	Cotonou MSM PrEP demonstration project, 2020–21;^10^ ESDG MSM survey (Grand Cotonou), 2022^16^
MSM aged 25–50 years	1·6–7·2	Cotonou MSM PrEP demonstration project, 2020–21;^10^ ESDG MSM survey (Grand Cotonou), 2022^16^
**Relative number of male sexual partners, high risk:low risk**		
MSM aged 18–24 years	1·0–2·9	Cotonou MSM PrEP demonstration project, 2020–21^10^
MSM aged 25–50 years	0·9–2·5	Cotonou MSM PrEP demonstration project, 2020–21^10^
**MSM anal sex acts in which a condom is used from 2015 onwards, %**		
Low-risk MSM aged 18–24 years	84–100%	Cotonou MSM PrEP demonstration project, 2020–21;^10^ ESDG MSM surveys (Grand Cotonou), 2017 and 2022^15,16^
High-risk MSM aged 18–24 years	45–71%	Cotonou MSM PrEP demonstration project, 2020–21;^10^ ESDG MSM surveys (Grand Cotonou), 2017 and 2022^15,16^
Low-risk MSM aged 25–50 years	76–96%	Cotonou MSM PrEP demonstration project, 2020–21;^10^ ESDG MSM surveys (Grand Cotonou), 2017 and 2022^15,16^
High-risk MSM aged 25–50 years	46–64%	Cotonou MSM PrEP demonstration project, 2020–21;^10^ ESDG MSM surveys (Grand Cotonou), 2017 and 2022^15,16^
**Average number of stable female sexual partners per 6 months**		
Low-risk MSM aged 18–24 years	0·3–0·8	Cotonou MSM PrEP demonstration project, 2020–21^10^
High-risk MSM aged 18–24 years	0·3–0·6	Cotonou MSM PrEP demonstration project, 2020–21^10^
Low-risk MSM aged 25–50 years	0·5–0·9	Cotonou MSM PrEP demonstration project, 2020–21^10^
High-risk MSM aged 25–50 years	0·6–1·0	Cotonou MSM PrEP demonstration project, 2020–21^10^
Vaginal sex acts in which a condom is used with stable female partners, %	34–52%	Cotonou MSM PrEP demonstration project, 2020–21^10^
**PrEP behaviour**		
New PrEP users who choose a daily regimen, %	80% (fixed)*	Cotonou MSM PrEP demonstration project, 2020–21^10^
Yearly per-capita rate of switching regimens from daily to on-demand	0·02–0·23	Cotonou MSM PrEP demonstration project, 2020–21^10^
Yearly per-capita rate of switching regimens from on-demand to daily	0·17–0·35	Cotonou MSM PrEP demonstration project, 2020–21^10^
Yearly per-capita rate of PrEP dropout	0·17 (fixed)†	Cotonou MSM PrEP demonstration project, 2020–21^10^
Full adherence to daily PrEP‡ (≥4 doses per week), %	Main: 11–20%, optimistic: 11–20%, ideal: 100% (fixed)	Cotonou MSM PrEP demonstration project, 2020–21^10^
Partial adherence to daily PrEP (2–3 doses per week), %	Main: 3–10%, optimistic: 23–35%, ideal: 0% (fixed)	Cotonou MSM PrEP demonstration project, 2020–21^10^
Full adherence to on-demand PrEP‡ (≥4 of 7 expected doses given sexual activity), %	Main: 15–35%, optimistic: 36–55%, ideal: 100% (fixed)	Cotonou MSM PrEP demonstration project, 2020–21^10^
Partial adherence to on-demand PrEP (2–4 of 7 expected doses given sexual activity), %	Main: 2–15%, optimistic: 2–15%, ideal: 0% (fixed)	Cotonou MSM PrEP demonstration project, 2020–21^10^
**Intervention efficacies**		
Reduction in annual HIV acquisition rate due to correct condom use	58–79%	US MSM estimat§
Reduction in HIV transmission risk due to HIV-infected partner being on ART and fully suppressed	100% (fixed)	European MSM estimat§
**Reduction in annual HIV acquisition rate for daily PrEP users, %**		
≥4 doses per week	90–100%	MSM trial data, daily regimen^6^
2–3 doses per week	56–96%	MSM trial data, daily regimen^6^
<2 doses per week	0% (fixed)	MSM trial data, daily regimen^6^
Overall reduction in HIV acquisition rate for daily PrEP users, weighted by adherence	Main: 14–23%, optimistic: 27–40%, ideal: 91–99%	Based on dose-specific reductions (above) and observed adherence levels during the demonstration project
**Reduction in annual HIV acquisition rate for on-demand PrEP users, %**		
≥4 of 7 expected doses given sexual activity	90–100%	Same efficacy as for daily TDF/FTC for MSM, with efficacy assumed to depend on the proportion of expected doses taken (appendix pp 24–26)
2–4 of 7 expected doses given sexual activity	56–96%	Same efficacy as for daily TDF/FTC for MSM, with efficacy assumed to depend on the proportion of expected doses taken (appendix pp 24–26)
<2 of 7 expected doses given sexual activity	0% (fixed)	Same efficacy as for daily TDF/FTC for MSM, with efficacy assumed to depend on the proportion of expected doses taken (appendix pp 24–26)
Overall reduction in HIV acquisition rate for on-demand PrEP users, weighted by adherence	Main: 19–38%, optimistic: 38–56%, ideal: 91–99%	Based on dose specific reductions (above) and observed adherence levels during the demonstration project
**Cost on PrEP, $**		
Cost per PrEP initiation	72(fixed)¶	Cotonou MSM PrEP demonstration project, 2020–21; costs adjusted for scale-up given planned staffing and services (appendix 1 p 28)
Cost per person-year, daily PrEP users	300 (fixed)‖	Cotonou MSM PrEP demonstration project, 2020–21; costs adjusted for scale-up given planned staffing and services (appendix 1 p 28)
Cost per person-year, on-demand PrEP users	213 (fixed)**	Cotonou MSM PrEP demonstration project, 2020–21; costs adjusted for scale-up given planned staffing and services (appendix 1 p 28)
**HIV care costs, $**		
Cost per ART initiation	202 (fixed)	TaSP and PrEP demonstration project for FSW in Cotonou^17^
Cost per person-year on ART	307(fixed)	TaSP and PrEP demonstration project for FSW in Cotonou^17^
Cost per person-year, HIV infected but not on ART	0–267	Chosen to satisfy lifetime costs of HIV care ($3619) from a global review;^21^ costs of 0 used when lifetime ART costs exceeded $3619
Lifetime cost of HIV care (ART costs plus other health-care costs)	3619–5423	As above; higher values used when lifetime ART costs exceeded $3619
**Fitting outcomes**		
Sexually active MSM population in 2018	1696–5087	Estimate from Réseau Bénin Synergie Plus† with 50% error, ESDG MSM survey (Grand Cotonou)^15,16^
Sexually active MSM population in 2022	1770–5930	Estimate from Réseau Bénin Synergie Plus§ with 50% error, ESDG MSM survey (Grand Cotonou)^15,16^
HIV prevalence among sexually active MSM in 2013, %	15–26%	ESDG MSM surveys (Grand Cotonou)§^15,16^
HIV prevalence among sexually active MSM in 2017, %	5–14%	ESDG MSM surveys (Grand Cotonou)§^15,16^
HIV prevalence among sexually active MSM in 2022, %	9–18%	ESDG MSM surveys (Grand Cotonou)§^15,16^
MSM living with HIV but on ART in 2017, %	22–56%	ESDG MSM survey (Benin-wide)^15^
MSM on ART who are virally suppressed in 2017, %	19–74%	ESDG MSM survey (Benin-wide)^15^
**Cross-validation data**		
HIV incidence per person per year among demonstration project participants	0·003–0·045	Cotonou MSM PrEP demonstration project, 2020–21^10^

See appendix 2 for all model parameters (pp 10–17) and model fitting outcomes (p 32). All cost data are presented as US$ at 2021 values. MSM are defined as at high risk if reporting inconsistent condom use or a partner living with HIV in the previous 6 months, or with a recent positive chlamydia or gonorrhea test; and low risk otherwise. MSM=men who have sex with men. PrEP=pre-exposure prophylaxis. ART=antiretroviral therapy. TDF/FTC=tenofovir disoproxil fumarate and emtricitabine. TasP=treatment as prevention. FSW=female sex workers.

* 5-year scale-up value 74–85%.

† 5-year scale-up value 0·12–0·23.

‡ 13–21% of PrEP users in total (daily and on-demand users combined) take at least four of seven required doses (ie, ≥4 doses per week for daily users and ≥4 of 7 expected doses given sexual activity for on-demand users).

§ References appear in appendix 2 (p 62).

¶ 5-year scale-up value 5 (fixed).

‖ 5-year scale-up value 105 (fixed).

** 5-year scale-up value 71 (fixed).

## Data Availability

All modelling and cost parameters can be found in appendix 2 (pp 4–32). The model code is available from the authors on request (trystan.leng@imperial.ac.uk). The study protocol and anonymised individual-level data from the demonstration project are available from the authors on request (souleymane.diabate@crchudequebec.ulaval.ca).

## Research in context

### Evidence before this study

A 12020–21 demonstration project assessed the feasibility and health outcomes of offering oral pre-exposure prophylaxis (PrEP) to men who have sex with men (MSM) in Cotonou, Benin. We searched PubMed for articles estimating the population-level impact or cost-effectiveness of offering PrEP to MSM in west Africa between Jan 1, 2012, and Oct 1, 2024, using the following search terms for titles and abstracts: (“PrEP” OR “pre-exposure prophylaxis”) AND (“HIV” OR “AIDS”) AND (“Benin” OR “Burkina Faso” OR “Cabo Verde” OR “Cape Verde” OR “Cote D’Ivoire” OR “Ivory Coast” OR “Gambia” OR “Ghana” OR “Guinea” OR “Guinea-Bissau” OR “Liberia” OR “Mauritania” OR “Niger” OR “Nigeria” OR “Senegal” OR “Sierra Leone” OR “West Africa”) AND (“men who have sex with men” OR “MSM” OR “gay” OR “homosexual” OR “bisexual” OR “same-sex”). None of the 44 studies identified estimated the impact of PrEP for MSM in Benin or west Africa.

### Added value of this study

We used an HIV transmission-dynamic model, parametrised with relevant data collected during the 2020–21 Cotonou demonstration project on behaviour, PrEP adherence, regimen choice, and retention. With this model, we estimated the impact of the 2020–21 demonstration project itself, as well as the scale-up of oral PrEP to MSM in Cotonou and Abomey-Calavi, Benin. Given the low adherence levels, as observed during the demonstration project, and the short project duration, the long-term effect of the PrEP demonstration project on HIV incidence was low. However, scaling up PrEP for 5 years to reach 30% coverage by 2027 could avert 2–5% of all HIV infections among MSM and their female partners over 20 years. To our knowledge, our study provides the first cost-effectiveness estimates of oral PrEP for MSM in west Africa. At the low adherence levels observed during the demonstration project, PrEP scale-up has an 87% probability of being cost-effective at the WHO threshold for cost-effectiveness (1 × Benin gross domestic product [GDP] per capita, US$1225) but only has a 46% probability of being cost-effective at a Benin-specific threshold based on health opportunity costs (0·29 × GDP per capita, $355). However, PrEP would be likely to be cost-effective at this threshold if high levels of adherence (all PrEP users taking at least four out of seven required doses) could be reached.

### Implications of all the available evidence

At sufficient scale, PrEP could help to reduce new HIV infections among MSM in Benin. Scaling up PrEP is likely to be a cost-effective intervention for MSM in Benin (at 1 × GDP per capita threshold), and it could become more cost-effective if adherence can be considerably improved through tailored interventions without substantially increasing costs.
