# Supplementary material for: Potential impact and cost-effectiveness of oral HIV pre-exposure prophylaxis for men who have sex with men in Cotonou, Benin: a mathematical modelling study
Source: Lancet Glob Health. Author manuscript; Available in PMC 2025 Sep 30. (PMC12483189; doi:10.1016/S2214-109X(25)00098-1)
Supplement: Supplementary Appendix 1 [file NIHMS2109324-supplement-Supplementary_Appendix_1.pdf]

THE LANCET
Global Health

Supplementary appendix 1

This translation in French was submitted by the authors and we reproduce it as supplied. It has not been peer reviewed. *The Lancet*'s editorial processes have only been applied to the original in English, which should serve as reference for this manuscript.

Cette traduction en français a été proposée par les auteurs et nous l'avons reproduite telle quelle. Elle n'a pas été examinée par des pairs. Les processus éditoriaux du *Lancet* n'ont été appliqués qu'à l'original en anglais et c'est cette version qui doit servir de référence pour ce manuscrit.

Supplement to: Leng T, Kessou L, Heitner J, et al. Potential impact and cost-effectiveness of oral HIV pre-exposure prophylaxis for men who have sex with men in Cotonou, Benin: a mathematical modelling study. *Lancet Glob Health* 2025; **13:** e1111–21.

Contexte: Nous avons évalué l'impact épidémiologique et le rapport coût-efficacité d'un projet pilote d'un an et d'un potentiel passage à l'échelle de la prophylaxie pré-exposition (PrEP) orale du VIH pour les hommes ayant des rapports sexuels avec d'autres hommes (HSH) à Cotonou, au Bénin.

Méthodes: Nous avons calibré un modèle dynamique de transmission du VIH structuré par âge et par risque dans un cadre bayésien sur des données de prévalence et de traitement du VIH spécifiques aux HSH, paramétré avec des données de comportements sexuels et les coûts du projet (y compris les coûts des médicaments de la PrEP, de la mise en œuvre du projet et des soins VIH). Nous avons estimé l'impact et le rapport coût-efficacité a) du projet pilote à Cotonou 2020-21 (5%-10% de couverture de la PrEP parmi les HSH séronégatifs au sein du Grand Cotonou, adhérence : 13-21% des HSH prenant ≥4/7 des doses requises, c'est-à-dire ≥4 doses/semaine pour les utilisateurs de la PrEP en continu et ≥4/7 des doses attendues chez les utilisateurs de la PrEP à la demande, en tenant compte leur activité sexuelle déclarée), et b) un potentiel passage à l'échelle de l'utilisation de la PrEP sur 5 ans, de 2022 à 2027, atteignant une couverture de 30% des HSH séronégatifs vivant à Grand Cotonou, et avec les niveaux d'adhérence du projet de démonstration. Nous avons également modélisé un scenario d'adhésion parfaite à la PrEP (100% des HSH sous PrEP prenant ≥4/7 doses requises).

Nous avons estimé la proportion de nouvelles infections à VIH cumulées évitées parmi i) les HSH participant au projet pilote sur 1 an et ii) tous les HSH de Grand Cotonou et leurs partenaires féminins sur 20 ans, et le rapport coût-efficacité calculé comme coût pour une année d'espérance de vie corrigée de l'incapacité (DALY) évitée sur 20 ans. Les coûts et les DALYs ont été actualisés de 3 % par an.

Résultats: Le projet pilote a permis d'éviter environ 21,5 % (intervalle d'incertitude à 95 % : 16,6-26,2 %) des nouvelles infections par le VIH chez les participants sur une période d'un an. Avec une adhérence idéale, ce chiffre serait passé à 95,2 % (90,8-98,8 %).

Un passage à l'échelle de l'utilisation de la PrEP sur 5 ans pourrait permettre d'éviter 3,2 % (1,6-4,8 %) des nouvelles infections à VIH chez tous les HSH et leurs partenaires féminins sur 20 ans, au prix de US$388 ($36-$2 792) par DALY évitée. Dans le cas d'une adhésion parfaite, ce coût serait réduit à -$28(-$126-$589).

Interprétation: La faible adhésion à la PrEP lors du projet pilote a limité son impact épidémiologique. Un passage à l'échelle de l'utilisation de la PrEP serait rentable à un seuil de US$1225 (le PNB par habitant du Bénin) avec une probabilité de 86,6 % et pourrait devenir plus rentable si une adhésion élevée pouvait être obtenue sans augmentation substantielle des coûts.

.
