# Supplementary material for: Potential impact and cost-effectiveness of oral HIV pre-exposure prophylaxis for men who have sex with men in Cotonou, Benin: a mathematical modelling study
Source: Lancet Glob Health. Author manuscript; Available in PMC 2025 Sep 30. (PMC12483189; doi:10.1016/S2214-109X(25)00098-1)
Supplement: Supplementary Appendix 2 [file NIHMS2109324-supplement-Supplementary_Appendix_2.pdf]

THE LANCET
Global Health

Supplementary appendix 2

This appendix formed part of the original submission and has been peer reviewed. We post it as supplied by the authors.

Supplement to: Leng T, Kessou L, Heitner J, et al. Potential impact and cost-effectiveness of oral HIV pre-exposure prophylaxis for men who have sex with men in Cotonou, Benin: a mathematical modelling study. *Lancet Glob Health* 2025; **13:** e1111–21.

Supplementary Information: Potential impact and cost-effectiveness of oral HIV pre-exposure prophylaxis for men who have sex with men in Cotonou, Benin: a mathematical modelling study

Trystan Leng, Léon Kessou, Jesse Heitner, Fernand A Guédou, Luc Béhanzin, Marius Olodo
Souleymane Diabaté, Romain Silhol, Dobromir Dimitrov, Peter Vickerman, Michel Alary
Marie-Claude Boily, Kate M Mitchell

Contents

1 Model **4**
1.1 Overview and model adaptations . . . 4
1.2 Model schematics . . . 5
1.3 Model equations . . . 7
1.3.1 Susceptible equations . . . 7
1.3.2 Acute infection equations . . . 7
1.3.3 Chronic infection equations . . . 8
1.4 Model parameters . . . 10
1.4.1 Data sources . . . 10
1.5 Modelling details . . . 18
1.5.1 Demography . . . 18
1.5.2 Sexual behaviour - MSM partnership dynamics . . . 19
1.5.3 Sexual behaviour - condom use . . . 19
1.5.4 Sexual behaviour - mixing between age and risk groups . . . 21
1.5.5 Sexual behaviour - female partnerships . . . 21
1.5.6 Transmission within MSM partnerships . . . 22
1.5.7 Transmission to female partners . . . 23
1.5.8 HIV progression . . . 23
1.5.9 PrEP adherence . . . 24
1.5.10 HIV testing . . . 26
1.5.11 ART eligibility . . . 26
1.6 Cost-effectiveness details . . . 27
1.6.1 Costs associated with HIV infection . . . 27
1.6.2 Costs associated with PrEP . . . 27
1.6.3 Calculating DALYs . . . 29
1.6.4 DALYs and costs for female partners of MSM . . . 30
1.6.5 Threshold analysis . . . 30
1.7 Model outputs . . . 31
1.8 Sensitivity analysis scenarios . . . 32

2 Model fitting **33**
2.1 Fitting outcomes . . . 33
2.2 Fitting algorithm . . . 33
2.3 Likelihood function . . . 33
2.4 Model posteriors . . . 34

3 Supplementary Results **42**

4 CHEERS checklist **56**

List of Tables

S1 Demographic and initial condition parameters. . . . 10
S2 Sexual behaviour with MSM partners parameters. . . . 11
S3 Sexual behaviour with female partners parameters. . . . 12
S4 HIV transmission related parameters. . . . 13
S5 HIV progression related parameters. . . . 14
S6 PrEP behaviour parameters. . . . 15
S7 Testing and ART behaviour parameters. . . . 16
S8 Cost-effectiveness relevant parameters. . . . 17
S9 Differences between main and optimistic adherence scenarios. . . . 26
S10 Differences in costing assumptions for the 1-year PrEP demonstration project and PrEP scale-up. . . . 28
S11 Summary of model outputs. . . . 31
S12 Sensitivity scenarios considered. . . . 32
S13 Fitting outcomes. . . . 33
S14 Comparing main results from 500 parameter sets vs 1000 parameter sets. . . . 34

List of Figures

S1 Model population structure. . . . 5
S2 Disease progression for individuals in different care states. . . . 5
S3 Testing, PrEP, and care states within the model. . . . 6
S4 MCMC trace plots and diagnostics. . . . 35
S5 Posterior distribution density plots for demographic and initial condition parameters. . . . 36
S6 Posterior distribution density plots for sexual behaviour parameters (partnerships). . . . 37
S7 Posterior distribution density plots for sexual behaviour parameters (condom use). . . . 38
S8 Posterior distribution density plots for HIV transmission related parameters. . . . 38
S9 Posterior distribution density plots for HIV progression related parameters. . . . 39
S10 Posterior distribution density plots for PrEP behaviour related parameters. . . . 40
S11 Posterior distribution density plots for HIV testing and ART behavioural parameters. . . . 41
S12 Trends in MSM partners in the fitted model. . . . 42
S13 Trends in condom use in the fitted model. . . . 42
S14 Trends in HIV testing rates among routinely testing MSM in the fitted model. . . . 43
S15 Model fits to population size, age distribution, and testing. . . . 43
S16 Comparison of overall prevalence and incidence trends between models fitted to overall prevalence and models additionally fitted to age-specific prevalence. . . . 44
S17 Comparison of prevalence trends between models fitted to overall prevalence and models additionally fitted to age-specific prevalence. . . . 45
S18 Comparison of prevalence and incidence trends for the 1-year PrEP demonstration project under different adherence scenarios. . . . 46
S19 PrEP regimen choice for the 1-year demonstration project. . . . 46
S20 The breakdown of costs over 20 years for the 1-year PrEP demonstration project in 2020. . . . 47
S21 Acceptance, coverage, and PrEP regimen choice for a 5-year PrEP scale-up from 2022. . . . 47
S22 Trends in HIV prevalence and incidence among sexually active MSM, for a 5-year PrEP scale up from 2022. . . . 48
S23 The breakdown of infections averted and costs over 20 years for a 5-year PrEP scale-up from 2022 with 30% coverage by 2027. . . . 48
S24 Relationship between adherence, coverage, impact and cost-effectiveness of a 5-year PrEP scale-up to MSM in Grand Cotonou from 2022. . . . 49

S25 The relationship between incidence and cost-effectiveness of a 5-year PrEP scale-up from 2022. 50
S26 Sensitivity analysis of DALYs averted and excess costs from a 5-year PrEP scale-up from 2022, with 30% coverage by 2027. 51
S27 Sensitivity analysis of DALYs averted and excess costs from a 20-year PrEP scale-up from 2022, with 30% coverage by 2027. 52
S28 Sensitivity analysis of the cost-effectiveness of a 1-year PrEP demonstration project in 2020. 53
S29 Sensitivity analysis of DALYs averted and excess costs from a 1-year PrEP demonstration project in 2020. . . 54
S30 Threshold analysis for the cost-effectiveness of a 5-year PrEP scale-up from 2022. 55

1 Model

1.1 Overview and model adaptations

In this study, we adapted an age- and risk- structured ordinary differential equation (ODE) model of HIV transmission among men who have sex with men (MSM) from a US context[1] to the context of Cotonou and Abomey-Calavi (Grand Cotonou), Benin, to evaluate the impact of cost-effectiveness of oral PrEP. We adapted the model to Benin's context by making several structural changes, by parameterising the model based on data from Benin or Africa where available (described in detail in Supplementary Section 1.4) and by calibrating the model to all demographic, behavioural, biological, intervention data available for MSM in Cotonou (described in detail in Supplementary Section 2). Model adaptations are detailed below.

1. Behavioural parameters (partnership, condom use) were based on data collected during the 2020-21 MSM oral PrEP demonstration project in Cotonou[2] and during previous biobehavioural surveys of MSM in Cotonou and Abomey-Calavi[3–5]. We describe these data sources in detail in Supplementary Section 1.4.

2. As in the US model, we modelled two sexually active age groups (MSM aged 18-24 years and MSM aged 25-50 years). We extended the US model by including a third age group of 50+-year-old MSM, to track relevant quantities among MSM in later life needed for the cost-effectiveness analysis. The model population structure is shown in Figure S1.

3. Because a substantial proportion of MSM in Cotonou are bisexual (57% of demonstration project participants[2]), we added an estimation of infections to female partners to the model using a risk-equation approach[6], parameterised using behavioural data collected during the 2020-21 MSM oral PrEP demonstration project in Cotonou.

4. We modelled five stages of HIV infection in our model: acute HIV infection and four stages of chronic HIV infection by CD4 count. Unlike the US model, we did not further stratify chronic HIV infection by set point viral load. Disease progression for individuals in different care states is shown in Figure S2. All disease progression parameters were based on African data[7–15].

5. Within the model, individuals could transition between different behavioural stages. For uninfected individuals, behavioural stages related to testing for HIV or taking PrEP. PrEP regimens implemented in the model were chosen to match PrEP regimens from the 2020-21 MSM oral PrEP demonstration project in Cotonou. Additionally, we introduced a compartment to track HIV progression among demonstration project participants after the end of the project. PrEP adherence parameters were based on data collected during the 2020-21 MSM oral PrEP demonstration project in Cotonou.

6. For infected individuals, behavioural stages also included engagement with HIV care, from HIV diagnosis to being on ART to stopping ART. Unlike the US model, we did not distinguish between diagnosed MSM who are linked or not linked to care, because of a lack of data to calibrate this to for Benin. Instead, MSM diagnosed with HIV transitioned to starting ART at a certain rate (dependent on time-varying ART eligibility based on Benin national guidelines). The testing, PrEP and HIV care stages within the model are shown in Figure S3.

Only two model parameters were based on studies that do not draw upon African data. In the absence of Africa-specific data for MSM,the individual-level effectiveness for condom use and ART use are based on studies of MSM in the US[16] and Europe[17] respectively. However, our model reflects Cotonou-specific levels of condom use and adherence to PrEP (through model parameterisation) and adherence to ART (through model calibration), which means that effective HIV reduction rates in the model differ considerably from reduction rates in the US and Europe.

Our model was written in C++ and compiled on Visual Studio Code v1.78.2 with the GCC C++ compiler. ODEs were solved numerically using a variable step size eighth order Runge-Kutta method[18].

Data analysis and plotting were performed in R (version 4.4.2), using the R packages tidyverse (v2.0.0), boot (v3-28.1), and ggplot2 (v3.5.1). Figure S24 was produced in Matlab (version R2022a).

1.2 Model schematics

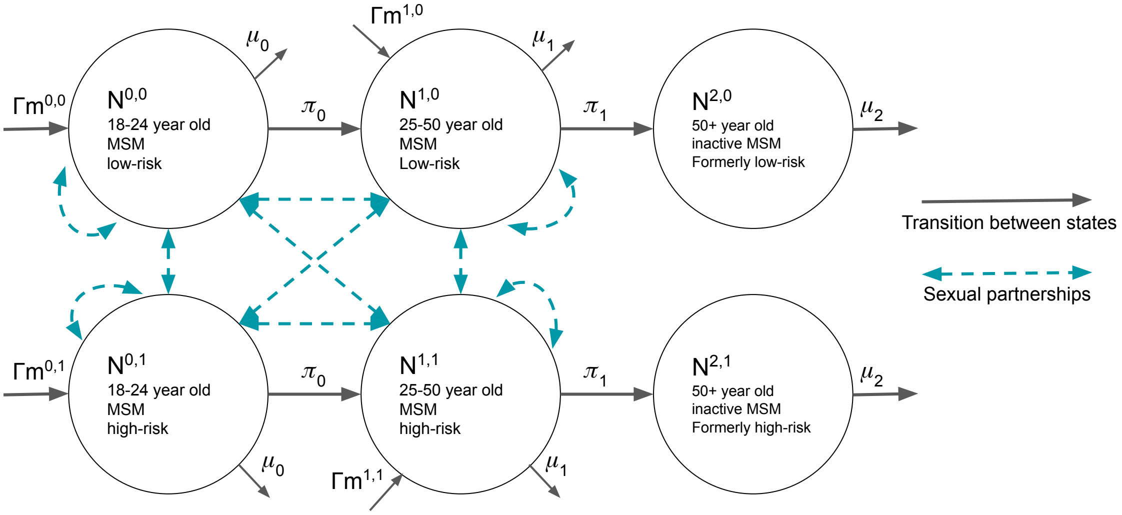

Figure S1: **Model population structure.** Age groups and risk groups within the model. Transitions between states are indicated by solid black arrows, and sexual partnerships between groups are indicated by blue dashed arrows.

Care state(s)	Explanation	Potential disease progression stages in care state(s)
Undiagnosed MSM<br><br>z ∈ {0,1,2,3,4}	Undiagnosed MSM may be susceptible, acutely infected, or chronically infected. If they become infected, they progress through different disease stages.	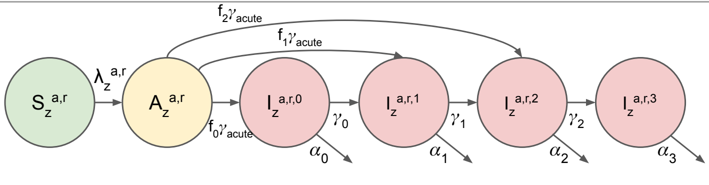
Diagnosed MSM who have not started ART<br><br>z = 5	Diagnosed MSM may be acutely infected or chronically infected. As they are not on treatment, they progress through different disease stages.	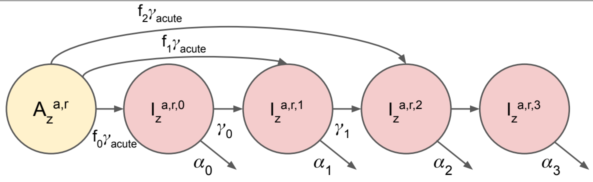
MSM on ART and who are adherent to ART<br><br>z ∈ {6,7}	MSM on ART are classed as chronically infected, with their initial category based on their CD4 count when initiating ART. Individuals do not transition between chronic stages, and have a reduced HIV related death rate.	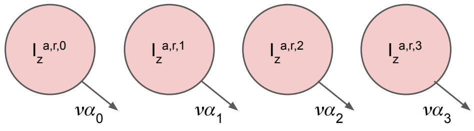
MSM on ART but are not adherent to ART or MSM who have stopped ART<br>z ∈ {8,9}	Those not adherent to treatment and those have stopped taking ART are classed as chronically infected. They progress through disease stages at the same rate as those who have not started treatment.	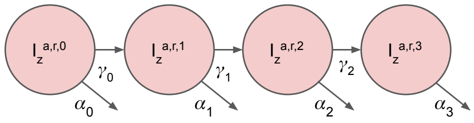

Figure S2: **Disease progression for individuals in different care states.** Possible transitions between different HIV disease stages for each of the 10 care states represented in the model. Model states are superscripted with a and r: a can take the value from 0 to 2 to specify a given age group, and r can take the value 0 or 1 to specify a risk group. Model states are subscripted with z, which can take a value from 0 to 9 the relevant care state.

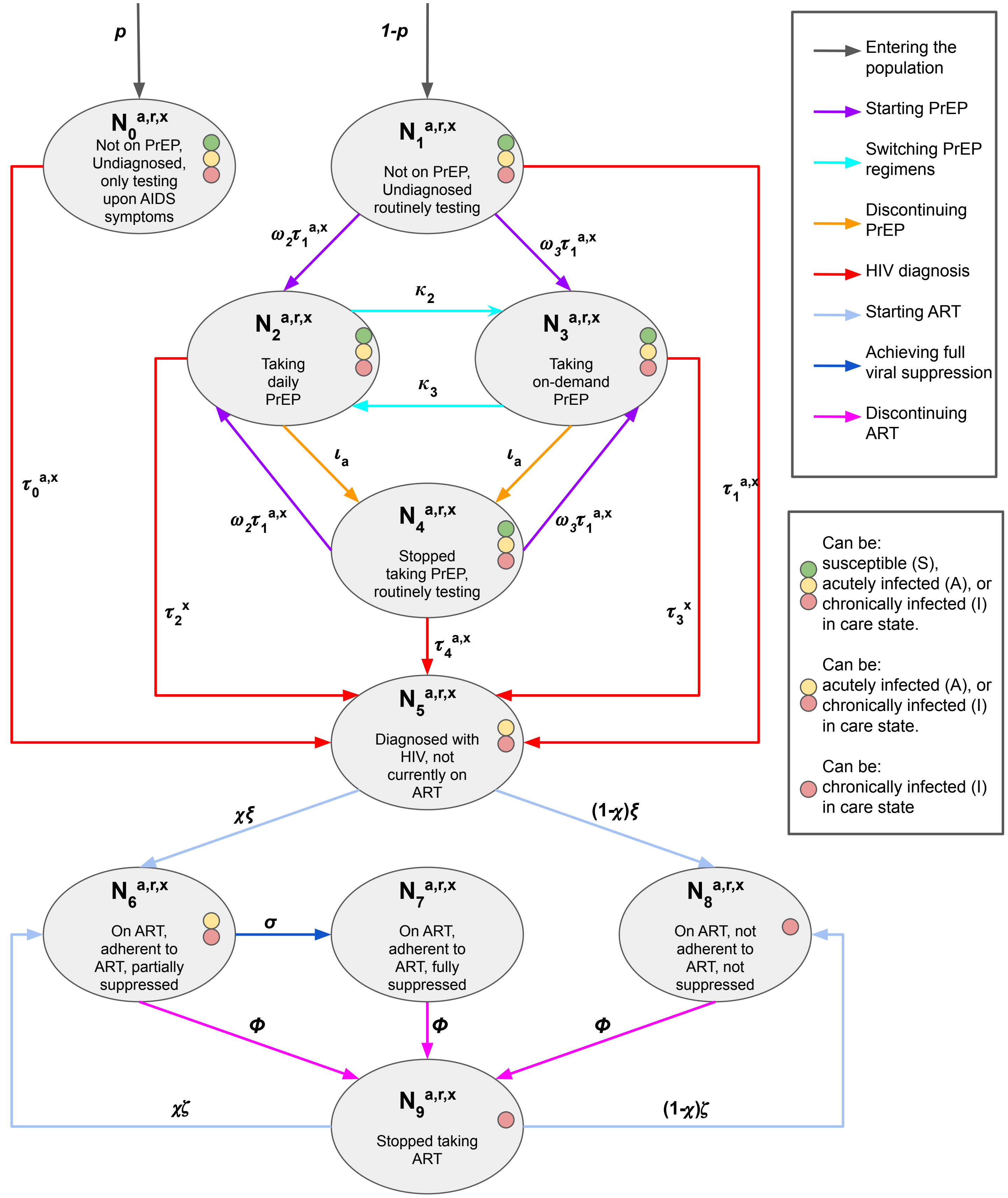

Figure S3: **Testing, PrEP, and care states within the model.** Transitions between different behavioural stages (testing for HIV, taking PrEP, HIV care) within the model. Model states are superscripted with a, r, and x: a can be replaced by a number from 0 to 2 to specify a given age group, r can be replaced by 0 or 1 to specify a risk group, and x can be replaced by 0,1,2, or 3 to specify a CD4 stage. Not all disease stages are available in every behavioural state - the disease states that are available are indicated by green, yellow, and red markers within each care state, and disease progression is given in detal in Figure S2. Arrows indicating different types of transitions are given different colours.

1.3 Model equations

1.3.1 Susceptible equations

We denote the number of susceptible individuals in age group a, risk group r, and care state z, as $S_z^{a,r}$. Within the model, MSM belong to one of three age groups, 18-24 years and sexually active ($a = 0$), 25-50 years and sexually active ($a = 1$), or 50+ years and not sexually active ($a = 2$). Within each age group, MSM belong to one of two 'risk' groups: low risk ($r = 0$) or high risk ($r = 1$). Susceptible individuals belong to five possible care state categories: not routinely testing ($z = 0$), testing but not on any PrEP regimen ($z = 1$), taking daily PrEP ($z = 2$), taking on-demand PrEP ($z = 3$), or having stopped taking PrEP ($z = 4$). Care states $z = 1$ and $z = 4$ are equivalent, but are modelled separately in order to quantify infections from individuals who have previously taken PrEP but have now stopped (in order to track the percentage of cumulative new HIV infections averted among demonstration project participants).

All rates are yearly rates. All MSM entering into the population are assumed to not be infected with HIV, with Γ denoting the entry rate into the MSM population. A proportion p of new entrants do not routinely test for HIV, and a proportion $m_{new}^{a,r}$ of new entrants enter into age group a and risk group r. Let π_a be the rate of moving from the age category a to age category $a+1$ (setting $\pi_{-1} = 0$ and $\pi_2 = 0$, for mathematical convenience) . Individuals have an age-specific non-HIV related death rate of μ_a. Let $\lambda_z^{a,r}$ be the force of infection to an individual in age group a, risk group r, and care state z. Individuals who are taking PrEP stop taking PrEP at an age-specific rate of ι_a. Routinely testing MSM and those who have stopped taking PrEP initiate onto a PrEP regimen z at a rate $\omega_z \tau_1^a = \omega_z \tau_4^a$, i.e. some proportion of MSM start taking PrEP upon testing for HIV. MSM who are taking PrEP can switch between regimens, with κ_2 the rate of switching from daily to on-demand PrEP, and κ_3 the rate of switching from on-demand to daily PrEP. After leaving the sexually active population, PrEP users are assumed to stop taking PrEP, moving to care state $z = 4$. For mathematical convenience, we define the states $S_z^{-1,r} = 0$. The equations for susceptible individuals are given below:

$$\frac{dS_0^{a,r}}{dt} = \Gamma p m_{new}^{a,r} + \pi_{a-1} S_0^{a-1,r} - S_0^{a,r}\left(\lambda_0^{a,r} + \mu_a + \pi_a\right) \tag{1}$$

$$\frac{dS_1^{a,r}}{dt} = \Gamma(1-p) m_{new}^{a,r} + \pi_{a-1} S_1^{a-1,r} - S_1^{a,r}\left(\lambda_1^{a,r} + \mu_a + \pi_a + \sum_{z=2}^{3} \omega_z \tau_1^a\right) \tag{2}$$

$$\frac{dS_2^{a,r}}{dt} = \begin{cases} \pi_{a-1} S_2^{a-1,r} + \omega_2(\tau_1^a S_1^{a,r} + \tau_4^a S_4^{a,r}) + \kappa_3 S_3^{a,r} - S_2^{a,r}\left(\lambda_2^{a,r} + \mu_a + \pi_a + \iota_a + \kappa_2\right) \text{ if } a < 2 \\ 0 \text{ if } a = 2 \end{cases} \tag{3}$$

$$\frac{dS_3^{a,r}}{dt} = \begin{cases} \pi_{a-1} S_3^{a-1,r} + \omega_3(\tau_1^a S_1^{a,r} + \tau_4^a S_4^{a,r}) + \kappa_2 S_2^{a,r} - S_3^{a,r}\left(\lambda_3^{a,r} + \mu_a + \pi_a + \iota_a + \kappa_3\right) \text{ if } a < 2 \\ 0 \text{ if } a = 2 \end{cases} \tag{4}$$

$$\frac{dS_4^{a,r}}{dt} = \begin{cases} \pi_{a-1} S_4^{a-1,r} + \iota_a \sum_{z=2}^{3} S_z^{a,r} - S_4^{a,r}\left(\lambda_4^{a,r} + \mu_a + \pi_a + \sum_{z=2}^{3} \omega_z \tau_4^a\right) \text{ if } a < 2 \\ \pi_{a-1} \sum_{z=2}^{4} S_z^{a-1,r} - S_4^{a,r}\left(\lambda_4^{a,r} + \mu_a + \pi_a\right) \text{ if } a = 2 \end{cases} \tag{5}$$

1.3.2 Acute infection equations

Acutely infected individuals $A_z^{a,r}$ can be in five undiagnosed care states analogous to the five states described above, alongside two additional care states: they can be diagnosed but not yet on ART ($z = 5$), or diagnosed, on ART, and partially virally suppressed ($z = 6$).

Let γ_{acute} be the rate of progression from acute to chronic HIV, let τ_z^a be the testing rate of an individual in age category a and care state z, let ξ_{acute} be the rate an acutely infected diagnosed individual starts ART, let σ denote the rate of achieving viral suppression after starting ART, let χ denote the proportion of new ART initiates who are adherent to ART, and let ϕ denote the rate of ART drop-out. For mathematical convenience, we define the categories $A_z^{-1,r} = 0$. Equations for acutely infected individuals are given by:

$$\frac{dA_0^{a,r}}{dt} = \pi_{a-1}A_0^{a-1,r} + \lambda_0^{a,r}S_0^{a,r} - A_0^{a,r}(\mu_a + \pi_a + \gamma_{acute}) \tag{6}$$

$$\frac{dA_1^{a,r}}{dt} = \pi_{a-1}A_1^{a-1,r} + \lambda_1^{a,r}S_1^{a,r} - A_1^{a,r}\left(\mu_a + \pi_a + \gamma_{acute} + \tau_1^a\right) \tag{7}$$

$$\frac{dA_2^{a,r}}{dt} = \begin{cases} \pi_{a-1}A_2^{a-1,r} + \lambda_2^{a,r}S_2^{a,r} + \kappa_3 A_3^{a,r} - A_2^{a,r}\left(\mu_a + \pi_a + \iota_a + \kappa_2 + \gamma_{acute} + \tau_2^a\right) \text{ if } a < 2 \\ 0 \text{ if } a = 2 \end{cases} \tag{8}$$

$$\frac{dA_3^{a,r}}{dt} = \begin{cases} \pi_{a-1}A_3^{a-1,r} + \lambda_3^{a,r}S_3^{a,r} + \kappa_2 A_2^{a,r} - A_3^{a,r}\left(\mu_a + \pi_a + \iota_a + \kappa_3 + \gamma_{acute} + \tau_3^a\right) \text{ if } a < 2 \\ 0 \text{ if } a = 2 \end{cases} \tag{9}$$

$$\frac{dA_4^{a,r}}{dt} = \begin{cases} \pi_{a-1}A_4^{a-1,r} + \lambda_4^{a,r}S_4^{a,r} + \iota_a \sum_{z=2}^{3} A_z^{a,r} - A_4^{a,r}\left(\mu_a + \pi_a + \gamma_{acute} + \tau_4^a\right) \text{ if } a < 2 \\ \pi_{a-1}\sum_{z=2}^{4} A_z^{a-1,r} + \lambda_4^{a,r}S_4^{a,r} - A_4^{a,r}\left(\mu_a + \pi_a + \gamma_{acute} + \tau_4^a\right) \text{ if } a = 2 \end{cases} \tag{10}$$

$$\frac{dA_5^{a,r}}{dt} = \pi_{a-1}A_5^{a-1,r} + \sum_{z=1}^{4} \tau_z^a A_z^{a,r} - A_5^{a,r}\left(\mu_a + \pi_a + \gamma_{acute} + \xi_{acute}\right) \tag{11}$$

$$\frac{dA_6^{a,r}}{dt} = \pi_{a-1}A_6^{a-1,r} + \chi\xi_{acute}A_5^{a,r} - A_6^{a,r}\left(\mu_a + \pi_a + \gamma_{acute} + \sigma + \phi\right) \tag{12}$$

1.3.3 Chronic infection equations

Chronically infected individuals $I_z^{a,r,x}$ can belong to one of four CD4 categories x: a CD4 count greater than 500cells/μl ($x = 0$), a CD4 count between 350 and 500 cells/μl ($x = 1$), a CD4 count between 200 and 350 cells/μl ($x = 2$), or a CD4 count less than 200 cells/μl ($x = 3$),

Chronically infected individuals can be in states corresponding to the seven states for acutely infected individuals. Alongside these, individuals can also become fully virally suppressed ($z = 7$), can be non-adherent to ART and not achieve viral suppression ($z = 8$), or can have dropped out of taking ART ($z = 9$). Let γ_x denote the rate of progression from CD4 category x to CD4 category $x + 1$, let α_x denote the HIV related death rate of those in CD4 category x, let χ denote the proportion of MSM initiating ART who are adherent, let ζ denote the re-initiation rate onto ART from drop-out, and let ν denote the relative rate of HIV-related death for individuals on ART. Let $\tau_z^{a,x}$ denote the testing rate of chronically individuals who are in CD4 category x, age category a, and care state z. Let f_x denote the proportion of individuals who, upon progressing from acute to chronic infection, have an initial CD4 count belonging to CD4 category x. For mathematical convenience, we define and set $\gamma_{-1} = \gamma_3 = 0$, and define the categories $I_z^{a,r,-1} = 0$ and $I_z^{-1,r,x} = 0$. Equations for chronically infected individuals are then given by:

$$\frac{dI_0^{a,r,x}}{dt} = \pi_{a-1}I_0^{a-1,r,x} + f_x\gamma_{acute}A_0^{a,r} + \gamma_{x-1}I_0^{a,r,x-1} - I_0^{a,r,x}\left(\mu_a + \pi_a + \gamma_x + \tau_0^{a,x} + \alpha_x\right) \tag{13}$$

$$\frac{dI_1^{a,r,x}}{dt} = \pi_{a-1}I_1^{a-1,r,x} + f_x\gamma_{acute}A_1^{a,r} + \gamma_{x-1}I_1^{a,r,x-1} - I_1^{a,r,x}\left(\mu_a + \pi_a + \gamma_x + \tau_1^{a,x} + \alpha_x\right) \tag{14}$$

$$\frac{dI_2^{a,r,x}}{dt} = \begin{cases} \pi_{a-1}I_2^{a-1,r,x} + f_x\gamma_{acute}A_2^{a,r} + \gamma_{x-1}I_2^{a,r,x-1} + \kappa_3 I_3^{a,r,x} - I_2^{a,r,x}\left(\mu_a + \pi_a + \iota_a + \kappa_2 + \gamma_x + \tau_2^{a,x} + \alpha_x\right) & \text{if } a < 2 \\ 0 \text{ if } a = 2 \end{cases} \tag{15}$$

$$\frac{dI_3^{a,r,x}}{dt} = \begin{cases} \pi_{a-1}I_3^{a-1,r,x} + f_x\gamma_{acute}A_3^{a,r} + \gamma_{x-1}I_3^{a,r,x-1} + \kappa_2 I_2^{a,r,x} - I_3^{a,r,x}\left(\mu_a + \pi_a + \iota_a + \kappa_3 + \gamma_x + \tau_3^{a,x} + \alpha_x\right) & \text{if } a < 2 \\ 0 \text{ if } a = 2 \end{cases} \tag{16}$$

$$\frac{dI_4^{a,r,x}}{dt} = \begin{cases} \pi_{a-1}I_4^{a-1,r,x} + f_x\gamma_{acute}A_4^{a,r} + \gamma_{x-1}I_4^{a,r,x-1} + \iota_a \sum_{z=2}^{3} I_z^{a,r,x} - I_4^{a,r,x}\left(\mu_a + \pi_a + \gamma_x + \tau_4^{a,x} + \alpha_x\right) & \text{if } a < 2 \\ \pi_{a-1}\sum_{z=2}^{4} I_z^{a-1,r,x} + f_x\gamma_{acute}A_4^{a,r} + \gamma_{x-1}I_4^{a,r,x-1} - I_4^{a,r,x}\left(\mu_a + \pi_a + \gamma_x + \tau_4^{a,x} + \alpha_x\right) & \text{if } a = 2 \end{cases} \tag{17}$$

$$\frac{dI_5^{a,r,x}}{dt} = \pi_{a-1}I_5^{a-1,r,x} + f_x\gamma_{acute}A_5^{a,r} + \gamma_{x-1}I_5^{a,r,x-1} + \left(\sum_{z=0}^{4}\tau_z^{a,x}I_z^{a,r,x}\right) - I_5^{a,r,x}\left(\mu_a + \pi_a + \gamma_x + \xi_x + \alpha_x\right) \tag{18}$$

$$\frac{dI_6^{a,r,x}}{dt} = \pi_{a-1}I_6^{a-1,r,x} + f_x\gamma_{acute}A_6^{a,r} + \chi\left(\xi_x I_5^{a,r,x} + \zeta I_9^{a,r,x}\right) - I_6^{a,r,x}\left(\mu_a + \pi_a + \sigma + \phi + \nu\alpha_x\right) \tag{19}$$

$$\frac{dI_7^{a,r,x}}{dt} = \pi_{a-1}I_7^{a-1,r,x} + \sigma I_6^{a,r,x} - I_7^{a,r,x}\left(\mu_a + \pi_a + \phi + \nu\alpha_x\right) \tag{20}$$

$$\frac{dI_8^{a,r,x}}{dt} = \pi_{a-1}I_8^{a-1,r,x} + (1-\chi)\left(\xi_{acute}f_x A_5^{a,r} + \xi_x I_5^{a,r,x} + \zeta I_9^{a,r,x}\right) - I_8^{a,r,x}\left(\mu_a + \pi_a + \gamma_x + \phi + \alpha_x\right) \tag{21}$$

$$\frac{dI_9^{a,r,x}}{dt} = \pi_{a-1}I_9^{a-1,r,x} + \phi\left(f_x A_6^{a,r} + \sum_{z=5}^{8} I_z^{a,r,x}\right) - I_9^{a,r,x}\left(\mu_a + \pi_a + \gamma_x + \alpha_x + \zeta\right) \tag{22}$$

1.4 Model parameters

1.4.1 Data sources

The behavioural parameters of men who have sex with men (MSM) within this study were primarily informed from secondary analysis of anonymised individual level data of survey responses collected during the 2020-21 MSM oral PrEP demonstration project in Cotonou[2] (where 204 participants were recruited via random walk sampling), and from responses given by participants from Cotonou and Abomey-Calavi who participated in 2017 and 2022 Enquete de Surveillance de Deuxieme Generation (ESDG) surveys[4,5] (164 participants in 2017, 286 participants in 2022, recruited via respondent-driven sampling). Participant weightings were not included in the data provided for the ESDG 2017 survey, but were available for 2022. The proportion of infected MSM on antiretroviral therapy (ART), and the proportion of MSM on ART who were virally suppressed, was informed by novel analyses of biological samples taken from HIV positive individuals in the ESDG 2017 survey. In the Benin-wide survey, 27 individuals were HIV positive for whom relevant data were obtained. In this instance, we used Benin-wide rather than Cotonou-specific data, due to the low numbers of individuals in the sample.

95% confidence intervals of data expressed as a proportion (e.g. the percentage of participants who have taken an HIV test in the last 12 months) were calculated assuming the data was drawn from a binomial distribution. Otherwise, 95% confidence intervals were calculated via bootstrapping, calculating weighted confidence intervals where this data was available (i.e. for ESDG 2022).

Table S1: **Demographic and initial condition parameters.**

**Description**	**Symbol**	**Prior**	**Source/notes**
Initial conditions			
Starting year of model runs	t_{init}	1980 (fixed)	Chosen to reflect epidemic in early stages in 1980s[19,20]
Initial size of sexually active MSM population	N_{init}	$U(550, 1815)$	Annual growth rate 18-50-year-old male Benin population 1980-2020 = 0.0331[21]. Grand Cotonou 15-50-year-old adult male population 2013 ≈ 330,000. Assume $0.5 - 1.65\%$ are MSM, in line with neighbouring countries[22]
HIV prevalence, 1980 (%)	-	$U(0.1, 2)$	Chosen to reflect epidemic in early stages in 1980s[19,20] and to capture uncertainty in prevalence given lack of Benin MSM prevalence data before 2013[3]
Demography			
Annual growth rate of MSM population (in the absence of HIV-related deaths)	G	$U(0.0231, 0.0431)$	Annual growth rate 18-50-year-old male Benin population 1980-2020 ± 0.01[21]
Yearly non-HIV related death rate			
- for 18-24-year-old MSM	$\mu(0)$	0.005 (fixed)	$\mu(0)$ and $\mu(1)$: central death rate estimate for 20-year-old men and 40-year-old men from 2000[23]
- for 25-50-year-old MSM	$\mu(1)$	0.0077 (fixed)	
- for 50+-year-old (sexually inactive) MSM	$\mu(2)$	0.044 (fixed)	$\mu(2)$: inverse of life expectancy for 50-year-old men in 2020
Yearly rate of moving to older age group			
- from 18-24 years to 25-50 years	$\pi(0)$	1/7 (fixed)	$\pi(0)$: mean age of sexual debut below 18 years old in Grand Cotonou in ESDG surveys[4,5],
- from 25-50 years to 50+years	$\pi(1)$	$U(0.04, 0.12)$	$\pi(1)$: Lower bound, leaving rate from ageing (1/25). Upper bound, adjusted to match age distribution of sexually active MSM.
% of new sexually active MSM who are 18-24 years old	m_{age}	$U(92.5, 97.7)$	ESDG MSM surveys 2017 and 2022 (Grand Cotonou specific data)[4,5]
% of new and initial sexually active MSM who are high risk	m_{risk}	$U(60, 73)$	Cotonou MSM PrEP demo[2]

Abbreviations. PrEP: pre-exposure prophylaxis, MSM: men who have sex with men

Table S2: **Sexual behaviour with MSM partners parameters.**

Description	Symbol	Prior	Source/notes
Sexual behaviour with MSM partners			
Mean no. of MSM partners per six months, prior to 2017:			
- 18-24-year-old MSM	$c_M^0(2017)$	$U(1.87, 3.96)$	Lower bound of 95% CI (for both age-groups) Cotonou MSM PrEP demo[2].
- 25-50-year-old MSM	$c_M^1(2017)$	$U(1.62, 4.27)$	Upper bound of 95% CI (for both age-groups) ESDG 2017 (Grand Cotonou specific data)[4]
Mean no. of MSM partners per six months, 2022 onwards:			
- 18-24-year-old MSM	$c_M^0(2022)$	$U(1.87, 5.51)$	Lower bound of 95% CI (for both age-groups): Cotonou MSM PrEP demo[2].
- 25-50-year-old MSM	$c_M^1(2022)$	$U(1.62, 7.24)$	Upper bound of 95% CI (for both age-groups): ESDG 2022 (Grand Cotonou specific data)[5]
Relative no. of MSM partners, high-risk: low-risk			
- 18-24-year-old MSM	k_0	$U(0.99, 2.86)$	95% CI Cotonou MSM PrEP demo[2].
- 25-50-year-old MSM	k_1	$U(0.89, 2.48)$	
% of MSM partnerships that are commercial:			
- 18-24-year-old MSM, low risk	$q_{0,0}$	$U(3, 6)$	95% CI combined sample from Cotonou MSM PrEP demo and ESDG 2022 (Grand Cotonou specific data)[5]
- 18-24-year-old MSM, high risk	$q_{0,1}$	$U(11, 17)$	
- 25-50-year-old MSM, low risk	$q_{1,0}$	$U(1, 2)$	
- 25-50-year-old MSM, high risk	$q_{1,1}$	$U(7, 10)$	
Mean no. of sex acts per six month non-commercial MSM partnership	n_M^{non}	$U(9.1, 12.9)$	95% CI Cotonou MSM PrEP demo[2].
Mean no. of sex acts per six month commercial MSM partnership	n_M^{comm}	$U(8.7, 15.4)$	95% CI Cotonou MSM PrEP demo[2].
% of sex acts in which a condom is used, prior to 1990	$s_M^{a,r}(1990)$	1 (fixed)	heterosexual population estimate Cameroon[24]. Assume same in Benin MSM population.
Year condom use among MSM increases from	Y_{condom}	$U(1990, 2000)$	Condom use among FSW increases during decade, condom use among general population remains low until 2000[25]. Assume MSM lie somewhere between
% of total sex acts in which a condom is used, 2015 onwards			
- 18-24-year-old MSM, low risk	$s_M^{0,0}(2015)$	$U(84, 100)$	95% CI Cotonou MSM PrEP demo[2]. Upper bounds increased to match condom use reported in ESDG 2017 and 2022[4,5]
- 18-24-year-old MSM, high risk	$s_M^{0,1}(2015)$	$U(45, 71)$	
- 25-50-year-old MSM, low risk	$s_M^{1,0}(2015)$	$U(76, 96)$	
- 25-50-year-old MSM, high-risk	$s_M^{1,1}(2015)$	$U(46, 64)$	
% of commercial sex acts in which a condom is used, 2015 onwards			
- 18-24-year-old MSM, low risk	$s_{comm}^{0,0}(2015)$	$U(52, 100)$	95% CI Cotonou MSM PrEP demo[2].
- 18-24-year-old MSM, high risk	$s_{comm}^{0,1}(2015)$	$U(53, 84)$	
- 25-50-year-old MSM, low risk	$s_{comm}^{1,0}(2015)$	$U(79, 98)$	
- 25-50-year-old MSM, high risk	$s_{comm}^{1,1}(2015)$	$U(41, 72)$	
Age mixing parameter, MSM partnerships	ϵ_{age}	$U(0, 0.7)$	From age of partners reported in ESDG 2017 and 2022[4,5]
Risk mixing parameter, MSM partnerships	ϵ_{risk}	$U(0, 1)$	Full range explored

Abbreviations. PrEP: pre-exposure prophylaxis, MSM: men who have sex with men

Table S3: **Sexual behaviour with female partners parameters.**

Description	Symbol	Prior	Source/notes
Sexual behaviour with female partners			
Mean no. of stable female partners per six months			
- 18-24-year-old MSM, low risk	$c_F^{0,0}(0)$	$U(0.33, 0.83)$	95% CI Cotonou MSM PrEP demo[2], spouses or regular female partners.
- 18-24-year-old MSM, high risk	$c_F^{0,1}(0)$	$U(0.34, 0.64)$	
- 25-50-year-old MSM, low risk	$c_F^{1,0}(0)$	$U(0.48, 0.90)$	
- 25-50-year-old MSM, high risk	$c_F^{1,1}(0)$	$U(0.59, 0.96)$	
Mean no. of sex acts per six months per stable female partnership	$n_F(0)$	$U(4.5, 19)$	Lower bound, half as frequent as MSM partners from Cotonou MSM PrEP demo[2]. Upper bound estimate for heterosexual partnerships used in modelling for FSW in Benin[25].
% of sex acts in which a condom is used with stable female partners	$s_F(0)$	$U(34, 52)$	95% CI Cotonou MSM PrEP demo[2], spouses or regular female partners from data at last sex act.
Mean no. of casual female partners per six months			
- 18-24-year-old MSM, low risk	$c_F^{0,0}(1)$	$U(0.08, 1.08)$	95% CI Cotonou MSM PrEP demo[2], occasional female partners or sex workers.
- 18-24-year-old MSM, high risk	$c_F^{0,1}(1)$	$U(0.28, 1.32)$	
- 25-50-year-old MSM, low risk	$c_F^{1,0}(1)$	$U(0.18, 1.06)$	
- 25-50-year-old MSM, high risk	$c_F^{1,1}(1)$	$U(0.48, 1.13)$	
Mean no. of sex acts per six months per casual female partnership	$n_F(1)$	$U(1, 3)$	Estimates for heterosexual partnerships used in modelling for FSW in Benin[25–27].
% of sex acts in which a condom is used with casual female partners	$s_F(1)$	$U(64, 86)$	95% CI Cotonou MSM PrEP demo[2], occasional female partners or sex workers from data at last sex act.

Abbreviations. PrEP: pre-exposure prophylaxis, MSM: men who have sex with men, FSW: female sex workers

Table S4: **HIV transmission related parameters.**

Description	Symbol	Prior	Source/notes
Transmission probability			
Average probability of HIV transmission per anal sex act between MSM, chronic stage	β_M	$U(0.0006, 0.0248)$	[28], adjusted assuming all MSM are circumcised and circumcision 13-21% effective, as in[29]
Average probability of HIV transmission per vaginal sex act to the female partner, chronic stage	β_F	$U(0.0009, 0.0043)$	[30], systematic review of heterosexual transmission risk, using estimate for developing countries.
Excess hazard months attributable to acute phase of infection*	$\frac{d_1-1}{\gamma_{acute}}$	$Triangular(-0.27, 64, 8.4)$	[31], estimate from heterosexual population in Uganda
Relative transmissibility of individuals with CD4 < 200cells/μl	d_2	$U(2, 8)$	[32], estimate from heterosexual population
Intervention efficacy			
Reduction (%) in HIV acquisition rate due to correct condom use, MSM partnerships	η^M_{condom}	$U(58, 79)$	[16], estimate from US MSM population
Reduction (%) in HIV acquisition rate due to correct condom use, heterosexual partnerships	η^F_{condom}	$U(58, 88)$	[33], meta-analyses of heterosexual couples in Africa
Reduction (%) in HIV transmission risk due to HIV-infected partner being on ART<br>- when fully virally suppressed<br>- when partially virally suppressed		100 (fixed)<br>50 (fixed)	Fully suppressed - estimates from European serodiscordant MSM couples with one virally suppressed[17]<br>Partially suppressed - assumption
Reduction (%) in HIV acquisition rate for daily or on-demand PrEP users with 'good adherence' (4 or more pills a week for daily users, $\geq 4/7$ expected pills taken given sexual activity for on-demand users)	$\eta_{PrEP}(0)$	$Triangular(90, 100, 96)$	Efficacy estimated from iPrEx and STRAND trial data analysis for 4 doses/week[34]
Relative efficacy of oral PrEP when a user is only 'partially adherent' (2-4 pills a week for daily users, 2/7 - 4/7 expected pills taken given sexual activity for on-demand users)	$\frac{\eta_{PrEP}(1)}{\eta_{PrEP}(0)}$	$Triangular(62, 96, 79)$	Chosen to obtain 56-96% efficacy for partial adherence[34]
Reduction in HIV acquisition rate for daily or on-demand PrEP when a user is non-adherent (< 2 pills a week for daily users < 2/7 expected pills taken given sexual activity for on-demand users)	$\eta_{PrEP}(2)$	0 (fixed)	consistent with available evidence from PrEP studies of limited benefit with low adherence[35–37]

* values constrained such that the relative infectiousness of acutely infected individuals, compared to chronically infected individuals, is between 4.47 and 18.81, the range of values obtained in a meta-analysis of heterosexual populations[30].

Abbreviations. PrEP: pre-exposure prophylaxis, MSM: men who have sex with men

Table S5: **HIV progression related parameters.**

Description	Symbol	Prior	Source/notes
Progression off ART			
HIV related yearly death rate for acutely infected individuals off ART or not adherent to ART	-	0	Assumption due to a lack of data. Consistent with low HIV-related death rates for individuals with CD4 > 500cells/μl[7,8]
HIV related yearly death rates* for chronically infected individuals off ART or not adherent to ART			data from African sources[7–9] with non-HIV related death rate of 35-year-old men in 2000 subtracted[23]
- CD4 > 500cells/μl	α_0	$U(0, 0.009)$	
- CD4 $350 - 500$cells/μl	α_1	$U(0, 0.034)$	$\alpha_0, \alpha_1, \alpha_2$: Combined range from[7,8]
- CD4 $200 - 350$cells/μl	α_2	$U(0, 0.114)$	
- CD4 < 200cells/μl	α_3	$U(0.193, 0.493)$	α_3: Combined range from[8,9]
Mean duration of acute HIV stage* (months)	$1/\gamma_{acute}$	$U(2, 6)$	Systematic review and analysis of data from Uganda[10,30]
Mean duration* (years), for those not on ART, from			
- CD4 > 500cells/μl to CD4 $350 - 500$cells/μl	$1/\gamma_0$	$U(3.2, 7.9)$	
- CD4 $350 - 500$cells/μl to CD4 $200 - 350$cells/μl	$1/\gamma_1$	$U(1.6, 5.6)$	from analysis of African data[8]
- CD4 $200 - 350$cells/μl to CD4 < 200cells/μl	$1/\gamma_2$	$U(3.6, 9.0)$	
% of chronically infected individuals with initial CD4 count of:			
- CD4 > 500cells/μl	f_0	55 (fixed)	
- CD4 $350 - 500$cells/μl	f_1	22 (fixed)	from analysis of
- CD4 $200 - 350$cells/μl	f_2	23 (fixed)	African data[8]
- CD4 < 200cells/μl	f_3	0 (fixed)	
Progression on ART			
Relative mortality for individuals on ART	ν	$U(0.16, 0.38)$	from South African data[11], by comparing life expectancy of suppressed and unsuppressed males.
Yearly rate from ART initiation to viral suppression	σ	$U(1.6, 3.2)$	South African data report $\approx 80\%$ virally suppressed after 6 months[12] African data report $\approx 80\%$ virally suppressed after 12 months[13,14]
Rate of moving to lower CD4 category when adherent to ART	-	0 (fixed)	Assumption

* Values constrained such that an unsuppressed individual will live for 8.7 - 14.2 years without treatment, based on East African data[15].

Abbreviations. ART: antiretroviral therapy

Table S6: **PrEP behaviour parameters.**

Description	Symbol	Prior	Source/notes
PrEP behaviour			
PrEP dropout rate per year	ι	demonstration: 0.165 (fixed)<br>scale-up: $U(0.12, 0.23)$	95% CI Cotonou MSM PrEP demo[2]
% of daily PrEP users with 'good adherence' i.e. taking 4 or more pills a week	$\theta_2(0)$	main: $U(11, 20)$<br>optimistic: $U(11, 20)$	95% CI Cotonou MSM PrEP demo[2], from TFV-DP measured M6 and M12, adherence scenarios explained in Section 1.5.9
% of on-demand PrEP users with 'good adherence' i.e. taking $\geq 4/7$ expected pills given their sexual activity	$\theta_3(0)$	main: $U(15, 35)$<br>optimistic: $U(36, 55)$	95% CI Cotonou MSM PrEP demo[2], from TFV-DP measured M6 and M12, adherence scenarios explained in Section 1.5.9
% of daily users without 'good adherence' who have 'partial adherence' i.e. taking 2 or 3 pills a week	$\theta_2(1)$	main: $U(3, 10)$<br>optimistic: $U(23, 35)$	95% CI Cotonou MSM PrEP demo[2], from TFV-DP measured M6 and M12, adherence scenarios explained in Section 1.5.9
% of on-demand users without 'good' adherence who have 'partial adherence' i.e. taking $2/7 - 4/7$ expected pills given sexual activity	$\theta_3(1)$	main: $U(2, 15)$<br>optimistic: $U(2, 15)$	95% CI Cotonou MSM PrEP demo[2], from TFV-DP measured M6 and M12, adherence scenarios explained in Section 1.5.9
Yearly rate of moving from daily PrEP to on-demand PrEP	κ_2	$U(2, 23)$	95% CI Cotonou MSM PrEP demo[2]
Yearly rate of moving from on-demand PrEP to daily PrEP	κ_3	$U(17, 35)$	95% CI Cotonou MSM PrEP demo[2]
% of new PrEP users who initially choose a daily regimen	$\omega_2/(\omega_2 + \omega_3)$	demonstration: 79.9 (fixed)<br>scale-up: $U(74, 85)$	95% CI Cotonou MSM PrEP demo[2]
# of MSM recruited onto 1-year demonstration project	-	204 (fixed)	# of participants on Cotonou MSM PrEP demo
% of MSM accepting PrEP at testing for HIV for scale-up	$\omega_2 + \omega_3$	Matched to obtain coverage levels by 2027	Range of coverage levels explored.

Abbreviations. PrEP: pre-exposure prophylaxis, MSM: men who have sex with men, TFV-DP: Tenofovir-diphosphate

Table S7: **Testing and ART behaviour parameters.**

Description	Symbol	Prior	Source/notes
Testing behaviour			
% of new MSM who do not routinely test for HIV, 2002	$p(2002)$	$U(8.6, 30)$	Lower bound from 95% CI of ESDG 2017[4]. Upper bound increased to explore wider range of changes between 2002 and 2015.
% of new MSM who do not routinely test for HIV, 2015 onwards	$p(2015)$	$U(0, 7)$	Upper bound from 95% CI of ESDG 2022[5]. Lower bound decreased to explore wider range of changes between 2002 and 2015.
Yearly HIV testing rate prior to 2002	$\tau_1^0(2002)$<br>$\tau_1^1(2002)$	0 (fixed)	Testing uncommon before introduction of ART, modelled similarly in[25]
% of MSM tested in last 12 months, 2015 onwards<br>- for 18-24-year-old MSM<br>- for 25+-year-old MSM	 <br>$1 - \exp(\tau_1^0(2015))$<br>$1 - \exp(\tau_1^1(2015))$	 <br>$U(67, 85)$<br>$U(78.5, 91)$	95% CI Cotonou MSM PrEP demo[2]
Relative rate of testing for individuals with CD4 < 200cells/μl	g	$U(1, 5.4)$	Estimate used in modelling for FSW in Benin[25,38]
Yearly HIV testing rate while on daily or on-demand PrEP	τ_{PrEP}	4 (fixed)	Testing every 3 months part of regimen
ART behaviour			
Yearly rate of initiation onto ART (when CD4 category is eligible)	ξ_x	$U(0, 1.5)$	Assumption, calibrated to match observed ART coverage in ESDG 2017[4]
% of those on ART who are adherent and will achieve viral suppression	χ	$U(0, 100)$	Full range explored, calibrated to ESDG 2017 data[4]
Yearly drop-out rate from ART	ϕ	$U(0.023, 0.11)$	general HIV infected population in West Africa[38] and Benin[39]
Yearly rate of re-initiation onto ART	ζ	$U(0.25, 1.5)$	Assumed same as female sex workers in Cotonou[25]

Abbreviations. MSM: men who have sex with men, ART: antiretroviral therapy

Table S8: **Cost-effectiveness relevant parameters.**

**Description**	**Symbol**	**Value or range**	**Source/notes**
Costs			
PrEP initiation costs per participant (US2021$)	$C_{PrEP}^{initiation}$	demonstration - 72 (fixed)<br>scale-up - 5.4 (fixed)	PrEP scale-up does not include Hepatitis B vaccination, STI screening, lab tests for transminases, has reduced staffing costs, and includes training costs in ongoing costs
Ongoing PrEP non-drug costs per participant per year (US2021$)	$C_{PrEP}^{ongoing}$	demonstration - 178 (fixed)<br>scale-up - 58 (fixed)	PrEP scale-up does not include lab tests for transaminases, has reduced staffing costs, but includes staff training costs annualised over 2 years
PrEP drug costs per participant per year for daily PrEP users (US2021$)	C_{PrEP}^{daily}	demonstration - 122 (fixed)<br>scale-up - 46.5 (fixed)	Scale-up costs reduced to reflect changes in prices of TDF/FTC, $3 per box of 30 doses in 2022.
PrEP drug costs per participant per year for on-demand PrEP users (US2021$)	$C_{PrEP}^{ondemand}$	demonstration - 35 (fixed)<br>scale-up - 13.4 (fixed)	Scale-up costs reduced to reflect changes in prices of TDF/FTC, $3 per box of 30 doses in 2022.
Cost per confirmatory HIV test for PrEP users who acquire HIV (US2021$)	$C_{conftest}$	4.8 (fixed)	Unit price of confirmatory HIV test
ART initiation costs per person (US2021$)	$C_{ART}^{initiation}$	202 (fixed)	Based on costing from TasP/PrEP demo for FSW in Cotonou[40].
Ongoing ART costs per person per year (US2021$)	$C_{ART}^{ongoing}$	307 (fixed)	Based on costing from TasP/PrEP demo for FSW in Cotonou[40].
Lifetime costs of care for HIV (US2021$)		3619–5423	Includes ART initiation costs, ongoing ART costs, and 'other' healthcare costs per person per year infected but not on ART over the course of a typical HIV infected individual's lifetime in 2020. Minimum value set at $3619[41] Higher values when ART costs exceed $3619.
Other healthcare costs per person year infected but not on ART (US2021$)	$C_{other}^{ongoing}$	0–267	Chosen to satisfy Lifetime costs of care for HIV $\geq$ $3619
DALY weights			
DALY weight of Life years uninfected	W_{uninf}	0 (fixed)	
DALY weight, Life years infected while on ART and adherent	W_{ART}	$U(0.052, 0.111)$	Global Burden of Disease estimates 2019[42]
DALY weight, Life years infected while not on ART or not adherent to ART<br>- (acute or CD4 $>$ 350cells/μl<br>- CD4 $200-350$cells/μl<br>- CD4 $<$ 200cells/μl	 <br>W_{early}<br>W_{mid}<br>W_{late}	 <br>$U(0.052, 0.111)$<br>$U(0.184, 0.377)$<br>$U(0.406, 0.703)$	Global Burden of Disease estimates 2019[42]
Discount rates			
Annual DALY discount rate (%)	D_{DALY}	3 (fixed)	[43]
Annual cost discount rate (%)	D_{cost}	3 (fixed)	Discounted at same rate as DALYs
Life expectancy			
Life expectancy in age category (years)<br>- for 18-24-year-old MSM<br>- for 25-50-year-old MSM	 $l(0)$<br>$l(1)$	 46 (fixed)<br>33 (fixed)	$l(0)$ and $l(1)$: life expectancy at midpoint of age group male Benin population, 2020[23]
- for 50+-year-old (sexually inactive) MSM	$l(2)$	15 (fixed)	$l(2)$: life expectancy at 60 years old, male Benin population, 2020[23]

Abbreviations. PrEP: pre-exposure prophylaxis, STI: sexually transmitted infection, ART: antiretroviral therapy, TDF/FTC: Tenofovir/emtricitabine, FSW: female sex workers, TasP: treatment as prevention, DALY: disability adjusted life year

1.5 Modelling details

1.5.1 Demography

The modelled proportion of MSM in each risk group was informed by participant survey responses in the 2020-21 Cotonou PrEP MSM demonstration project at initiation. Our criteria for 'high risk' was based upon the CDC eligibility criteria for PrEP[44]. Specifically, we considered a participant to be high risk if they reported having anal sex with a man in the past 6 months AND:

- EITHER having a known HIV positive partner in the past six months.
- OR inconsistent condom use over the past six months, i.e. having reported not always using a condom in the last six months during insertive or receptive anal sex.
- OR a positive test for chlamydia or gonorrhoea from their urine sample or anal swab at recruitment.

Within the model, individuals remained within the same risk-group for the entirety of their sexually active life. We assumed the majority of men entered the sexually active population in the 18-24-year-old category, with a small minority (2.3.-7.5%) at 25-50 years, consistent with reported ages of sexual debut from the ESDG behavioural surveys of MSM in Grand Cotonou[4,5]. MSM who entered the sexually active population in the 18-24-year-old category were assumed to enter at 18 years old, as the mean age of sexual debut for such individuals was below 18 years of age in the aforementioned reports. Accordingly, we assumed that 18-24-year-old MSM move to the 25-50 years category at a yearly rate of 1/7.

Population growth rates were chosen such that the sexually active MSM population would grow at a rate G in the absence of HIV-related deaths. Letting $N^{a,r}$ denote the total number of individuals in the population belonging to age group a and risk group r , i.e. $N^{a,r} = \left[\sum_{z=0}^{4} S_z^{a,r} + \sum_{z=0}^{6} A_z^{a,r} + \sum_{z=0}^{9}\sum_{x=0}^{3} I_z^{a,r,x}\right]$, N_a denote the total number of individuals in age group a, i.e. $N_a = \sum_{r=0}^{1} N^{a,r}$, N denote the total size of the sexually active population of MSM , i.e. $N = \sum_{a=0}^{1} N_a$, $m^{a,r}$ denote the proportion of sexually active MSM in age group a and risk group r, i.e. $m^{a,r} = N^{a,r}/N$, and $m_a = \sum_{r=0}^{1} m^{a,r}$

For a population to grow at a yearly rate G, the entry rate into the population must account for the rate that individuals leave the population, i.e. if $\mu_0 N_0 + (\mu_1 + \pi_1)N_1$ MSM leave the sexually active population in a year, $GN + \mu_0 N_0 + (\mu_1 + \pi_1)N_1$ must enter the population. In this instance, the proportion of 18-24-year-old MSM (m_0) is governed by the following ODE:

$$\frac{dm_0}{dt} = G[m^0_{new} m_1 - m^1_{new} m_0] + m^0_{new}(\pi_1 + \mu_1)m_1 - (\pi_0 + m^1_{new}\mu_0)m_0 \tag{23}$$

Assuming the HIV-related death rate is negligible prior to 1980, the starting year of model runs, we choose the initial proportion of MSM who are 18-24 years old to be the equilibrium proportion of 18-24-year-old MSM in this system, i.e. we set:

$$m_0(t_{init}) = \frac{m^0_{new}(G + \pi_1 + \mu_1)}{G + \pi_0 + m^1_{new}\mu_0 + m^0_{new}\pi_1 + m^0_{new}\mu_1} \tag{24}$$

The adjusted growth-rate, Γ, satisfies the MSM population growing at a rate G in the absence of HIV-related deaths:

$$\Gamma = (GN(t_{init}) + \mu_0 N_0(t_{init}) + (\mu_1 + \pi_1)N_1(t_{init})) \times e^{G(t-t_{init})} \tag{25}$$

The proportion of new entries who joined the MSM population in each age and risk group combination, $m^{a,r}_{new}$, is given by:

$$m^{0,0}_{new} = m_{age}(1 - m_{risk}) \tag{26}$$
$$m^{0,1}_{new} = m_{age} m_{risk} \tag{27}$$
$$m^{1,0}_{new} = (1 - m_{age})(1 - m_{risk}) \tag{28}$$
$$m^{1,1}_{new} = (1 - m_{age}) m_{risk} \tag{29}$$

1.5.2 Sexual behaviour - MSM partnership dynamics

We combined data from the 2020-21 Cotonou MSM PrEP demonstration project with data from ESDG surveys in 2017 and 2022 respectively, to estimate the mean number of MSM partners an individual from age group a has every six months ($c_M^a(2017)$ and $c_M^a(2022)$) prior to 2017 and from 2022 onwards, respectively. $c_M^a(t)$ varies linearly between these two time points. On the basis of the structure of the data collected, we assumed that an individual in age group a and risk group r 'forms' a partnership lasting six months at a yearly rate of $2c_M^{a,r}$. In reality, many of these newly 'formed' six-month partnerships will be continuations of existing partnerships. However, as a high proportion (67%) of demonstration project participants recorded being involved in a concurrent partnership at initiation, our approach captures the possibility of infection from a partnership in which each partner was initially susceptible but may have become infected at a later time. Given the lack of data on MSM aged 50+ years in Cotonou, Benin, we do not model the sexual activity of this age group, i.e. we set $c_M^{2,r} = 0$.

Based on the number of partners reported among low-risk MSM and high-risk MSM in the 2020-21 Cotonou MSM PrEP demonstration project, we assumed that the rate of partnership change varies between risk groups, with the ratio between the number of partners between low-risk and high-risk MSM in age group a given by k_a. We assume a proportion of partnerships $q^{a,r}$ formed by individuals in age group a and risk group r are commercial, based on data from the 2020-21 Cotonou MSM PrEP demonstration project and ESDG 2022. The proportion of MSM reporting commercial partnerships differed considerably between these two data sources (27% of demonstration project participants reporting a commercial partnership, 1% of ESDG 2022 participants reporting a commercial partnership). To reconcile this difference, we estimated 95% confidence intervals from combining the two data sources into one sample, using reported condom use as a proxy for an individual's risk group for ESDG 2022. Specifically, letting $c_M^{a,r}(j)$ denote the mean number of partners of type j ($j = 0$: non-commercial, $j = 1$: commercial) for an individual in age group a and risk group r every six months, we assume:

$$c_M^{a,0}(0) = (1 - q^{a,0}) c_M^a \frac{N_a}{N^{a,0} + k_a N^{a,1}} \tag{30}$$

$$c_M^{a,0}(1) = q^{a,0} c_M^a \frac{N_a}{N^{a,0} + k_a N^{a,1}} \tag{31}$$

$$c_M^{a,1}(0) = (1 - q^{a,1}) k_a c_M^a \frac{N_a}{N^{a,0} + k_a N^{a,1}} \tag{32}$$

$$c_M^{a,1}(1) = q^{a,1} k_a c_M^a \frac{N_a}{N^{a,0} + k_a N^{a,1}} \tag{33}$$

such that $\sum_{j=0}^{1} \sum_{r=0}^{1} c_M^{a,r}(j) N_M^{a,r} / N_M^a = c_M^a$.

During the 2020-21 Cotonou MSM PrEP demonstration project, an individual's number of MSM partners in the last six months were recorded at initiation, alongside the average number of monthly sex acts that participant has had over those six months. Over the course of each (six-month) partnership, sex acts occur at a rate of n_M^{non} and n_M^{comm} (dependent on partnership type), based on the average number of monthly sex acts reported by participants reporting exclusively non-commercial and exclusively commercial partnerships respectively, i.e. the number of sex acts per non-commercial (commercial) partnership per six months are drawn from a Poisson distribution with mean n_M^{non} (n_M^{comm}).

1.5.3 Sexual behaviour - condom use

Consistent with levels of condom use among heterosexuals in neighbouring Cameroon[24], we assumed that 1% of sex acts between MSM are protected by a condom until 1990. Among the general heterosexual population in Benin, condom use remained low until 2000; in contrast, condom use among female sex workers (FSWs) increased throughout the decade[25]. We assumed that MSM lie somewhere between these extremes, with condom use increasing from $Y_{condom} \sim U(1990, 2000)$. This proportion is assumed to increase until 2015, the year in which the highest national level of condom use among MSM was reported in MSM ESDG surveys nationwide. After this, condom use is assumed to remain constant. Within the model, the level of condom use varies by age, risk, and partnership type:

$$s_j^{a,r}(t) = \begin{cases} 0.01 \text{ if } t < Y_{condom} \\ \frac{(s_j^{a,r}(2015) - 0.01)(t - Y_{condom})}{2015 - Y_{condom}} + 0.01 \text{ if } Y_{condom} < t < 2015 \\ (s_j^{a,r}(2015), \text{ otherwise} \end{cases} \tag{34}$$

Let $s^{a,r,a',r'}(j)$ denote condom use in a partnership of type $j, j \in \{0 = \text{non-commercial}, 1 = \text{commercial}\}$ between an individual of age group a and risk group r with their partner of risk group a' and risk group r', and $\rho^{a,r,a',r'}(j)$ the proportion of an individual of age group a and risk group r's partnerships of type j that are with an individual of age group a' and risk group r'. We have the following equation for commercial partnerships:

$$s_{comm}^{a,r} = \sum_{a'=0}^{1} \sum_{r'=0}^{1} \left(s^{a,r,a',r'}(1) \times \rho^{a,r,a',r'}(1) \right) \tag{35}$$

For non-commercial partnerships, our estimates for $s_M^{a,r}$ and $s_{comm}^{a,r}$ must be taken into account:

$$s_M^{a,r} = q^{a,r} s_{comm}^{a,r} + (1 - q^{a,r}) \sum_{a'=0}^{1} \sum_{r'=0}^{1} \left(s^{a,r,a',r'}(0) \times \rho^{a,r,a',r'}(0) \right) \tag{36}$$

$$\iff \frac{s_M^{a,r} - q^{a,r} s_{comm}^{a,r}}{1 - q^{a,r}} = \sum_{a'=0}^{1} \sum_{r'=0}^{1} \left(s^{a,r,a',r'}(0) \times \rho^{a,r,a',r'}(0) \right) \tag{37}$$

By assuming that $s^{a,r,a',r'}(j)$ terms are linear functions of terms $b_j^{a,r}$ and $b_j^{a',r'}$, these equations can be solved via Gaussian elimination[18] to obtain target levels of condom use for each age and risk group. For partnerships with individuals from the same risk-group, condom use is averaged over $b_j^{a,r}$ values. For partnerships containing low and high-risk individuals, condom use is determined by the lower risk individual within a partnership, i.e.:

$$s^{a,r,a',r'}(j) = b^{a,r}(j), \text{ if } a = 0 \text{ and } r' = 1 \tag{38}$$

$$s^{a,r,a',r'}(j) = b^{a',r'}(j), \text{ if } r = 1 \text{ and } r' = 0 \tag{39}$$

$$s^{a,r,a',r'}(j) = (b^{a,r}(j) + b^{a',r'}(j))/2, \text{ if } r = r' \tag{40}$$

Finally, we constrain $s^{a,r,a',r'}(j)$ values to be between 0 and 1.

We used demonstration project participants' answers to questions regarding condom use at enrollment, month 6 and month 12 to inform the proportion of total sex acts with an MSM partner in which a condom is used ($s_M^{a,r}$), and the proportion of sex acts during a commercial partnership in which a condom is used ($s_{comm}^{a,r}$). We used responses about condom use at last sex act to inform $s_M^{a,r}$, assuming that these were a random sample of sex acts within an age and risk group. Participants were not asked about condom use at last sex act with commercial partners, but were asked whether they always (10 out of 10 times), often (5-9 out of 10 times), sometimes (1-4 out of 10 times) or never (0 out of 10 times) used a condom during all partnerships and commercial partnerships specifically. For data on all partnerships, we calculated the proportion of MSM who used a condom at their last sex act for each response. We then inferred the proportion who used a condom during their last commercial sex act, with $s_{comm}^{a,r}$ calculated assuming that these were a random sample of sex acts within an age and risk group. Upper bounds of the priors of $s_M^{a,r}$ and $s_{comm}^{a,r}$ were increased to reflect the higher level of condom use reported in ESDG 2017 and 2022.

1.5.4 Sexual behaviour - mixing between age and risk groups

The proportion of an individual in age group a and risk group r's partners of type j who are in age group a' and risk group r', defined by $\rho^{a,r,a',r'}(j)$, is determined by the number of partnerships offered by individuals in each age group and risk group, alongside two mixing parameters (ϵ_{age} and ϵ_{risk}). At $\epsilon_{age} = \epsilon_{risk} = 0$, there is proportional mixing between age and risk groups ($\rho^{a,r,a',r'}(j)$ is determined completely by the proportion of all partnerships offered by individuals in age group a' and risk group r'), while at $\epsilon_{age} = \epsilon_{risk} = 1$ there is assortative mixing between age and risk groups (individuals form partnerships exclusively with others in the same age and risk group). In the absence of relevant data, we set the prior ϵ_{risk} to be $U(0, 1)$. The prior range for ϵ_{age} was informed from participants' responses to whether they had partners of different ages (in 5-year age categories) in the previous year, from ESDG 2017 and 2022. While these data could tell us whether participants had partners in different age groups, it could not tell us how many partners of different ages individuals had. However, in some cases this could be inferred from this data together with reported partnership numbers. To account for this uncertainty, we calculated empirical age mixing matrices under the extreme assumptions that i) all unaccounted for partners were from the same age group and ii) all unaccounted for partners were from the other age group. We then found numerically the value of ϵ_{age} that minimised the distance between the modelled mixing matrix and empirical mixing matrix. Doing so, we set the prior to be $U(0, 0.7)$, i.e. we excluded the possibility of very high levels of assortativity by age. We assumed that mixing by age and risk are independent of one another; accordingly $\rho^{a,r,a',r'}$ is given by :

$$
\begin{aligned}
\rho^{a,r,a',r'}(j) =& \epsilon_{age}\epsilon_{risk}\delta_a(a')\delta_r(r') + \delta_a(a')\epsilon_{age}(1-\epsilon_{risk})\frac{N^{a',r'}c_M^{a',r'}(j)}{\sum_{r''=0}^{1} N^{a',r''}c_M^{a',r''}(j)} \\
&+ \delta_r(r')(1-\epsilon_{age})\epsilon_{risk}\frac{N^{a',r'}c_M^{a',r'}(j)}{\sum_{a''=0}^{1} N^{a'',r'}c_M^{a'',r'}(j)} + (1-\epsilon_{age})(1-\epsilon_{risk})\frac{N^{a',r'}c_M^{a',r'}(j)}{\sum_{a''=0}^{1}\sum_{r''=0}^{1} N^{a'',r''}c_M^{a'',r''}(j)}
\end{aligned} \tag{41}
$$

1.5.5 Sexual behaviour - female partnerships

In the 2020-21 demonstration project, 57% of participants identified as bisexual. While female partners are not included dynamically in our model, we estimated the cumulative number of HIV infections to female partners from a partnership with an MSM using a risk equation, an approach used by previous studies[6,45]. During the 2020-21 MSM PrEP demonstration project, participants were asked if they had spousal, regular, casual, or commercial female partners, over the last six months. This information, combined with the number of female partners reported in the last six months, could be used to infer the number of female partners that were stable or casual.

Given the small fraction of MSM participants (only 6%) who reported having sex with commercial female partners compared to participants who reported having sex with commercial or casual female partners (26%), we categorised the data on female partnerships recorded as either stable (spouses or regular partners) or casual (occasional or commercial partners). Delineating heterosexual behaviour by commercial/non-commercial sex would not have allowed us to capture as much information from the data to inform the model as the stable/casual distinction used. As MSM were not asked an analogous question for male partners, it was not possible to infer the number of partners for each type in the same way.

We used the following approach to infer the number of female partners of each type: consider a participant who records X female partners in the last 6 months, and let Y_{spouse}, $Y_{regular}$, Y_{casual}, and $Y_{commercial}$ denote indicator functions for Yes responses to having had a female partner of each type

Let X_{stable} and X_{casual} denote their number of stable (casual) female partners:

1. If $X = Y_{spouse} + Y_{regular} + Y_{casual} + Y_{commercial}$, then $X_{stable} = Y_{spouse} + Y_{regular}$ and $X_{casual} = Y_{casual} + Y_{commercial}$
2. else if $Y_{spouse} + Y_{regular} > 0$ and $Y_{casual} + Y_{commercial} = 0$, then $X_{stable} = X$, $X_{casual} = 0$
3. else if $Y_{casual} + Y_{commercial} > 0$ and $Y_{spouse} + Y_{regular} = 0$, then $X_{stable} = 0$, $X_{casual} = X$
4. else if $Y_{spouse} + Y_{regular} + Y_{casual} + Y_{commercial} = 0$, then $X_{stable} = X_{casual} = X/2$.
5. otherwise, $C_{stable} = Y_{spouse} + Y_{regular}$ and $C_{casual} = Y_{casual} + Y_{commercial} + \max[0, C - (Y_{spouse} + Y_{regular} + Y_{casual} + Y_{commercial})]$, i.e. all unaccounted for partnerships are classed as casual.

We used data on female partnerships to estimate the mean number of female partners of type j ($c_F^{a,r}(j)$, $j=0$: stable, $j=1$: casual) an MSM from age group a and risk group r has per six months. ESDG data was not used in our estimates of female partnership numbers. Data on the number of female partnerships per six months was not available for ESDG 2017 or ESDG 2022. Female partnerships per 12 months were recorded in ESDG 2022; in the survey 18-24-year-old MSM reported 0.7 (95% UI: 0.4–1.0) female partners per 12 months, while 25-50-year-old MSM reported 1.3 (95% UI: 0.6–1.9) female partners per 12 months. While these estimates were not directly used to inform parameter ranges, these partnership numbers are broadly consistent with partnerships per six months observed during the demonstration project. The mean number of sex acts per female partnership per six months depended on the type of partnership. In the absence of survey data from MSM on the frequency of sex acts with female partners, we considered a wide range of rates of sexual activity in stable female partnerships, from assuming that stable female partnerships have half the number of sex acts per partnership per six months as non-commercial MSM partnerships, to assuming that MSM partnership with female partners have sex as often as the general heterosexual population[25,46]. We assumed that the mean number of sex acts per casual female partnership is the same as observed between heterosexual men and FSW[25–27].

1.5.6 Transmission within MSM partnerships

Letting $d_A(z)$ be the relative infectiousness of an acutely infected individual in care state z, relative to an untreated chronically infected individual with CD4 count $\geq 200\text{cells}/\mu l$, we set:

$$d_A(z) = \begin{cases} d_1 \text{ if } z \in \{0,1,2,3,4,5\} \\ 0.5 \times d_1 \text{ if } z = 6 \end{cases} \tag{42}$$

Letting $d_I(z,x)$ be the relative infectiousness of a chronically infected individual in care state z with CD4 count x, relative to an untreated chronically infected individual with CD4 count $\geq 200\text{cells}/\mu l$, we set:

$$d_I(z,x) = \begin{cases} 1 \text{ if } z \in \{0,1,2,3,4,5,8,9\} \text{ and } x \in \{0,1,2\} \\ 0.5 \text{ if } z = 6 \text{ and } x \in \{0,1,2\} \\ d_2 \text{ if } z \in \{0,1,2,3,4,5,8,9\} \text{ and } x = 3 \\ 0.5 \times d_2 \text{ if } z = 6 \text{ and } x = 3 \\ 0 \text{ if } z = 7 \end{cases} \tag{43}$$

We let $\theta_z(h)$ denote the proportion of individuals in care state $z \in \{2,3\}$ who have PrEP adherence level $h, h \in \{0 = \text{good adherence}, 1 = \text{partial adherence}, 2 = \text{non-adherence}\}$, and let $\eta_{PrEP}(h)$ denote the efficacy of PrEP given adherence level h. We let η^M_{condom} denote the efficacy of condom use for anal sex acts between MSM. Within the model, interventions η^M_{condom} and η_{PrEP} scale the probability of infection per partnership rather than per sex act, which aligns more closely with the efficacies presented in their respective sources[16,33,34], which describe efficacies at an individual rather than a per sex act level.

Assuming that over the course of a partnership of type j, sex acts occur at a constant rate n^j_M per partnership, the probability of infection to an uninfected MSM in age group a, risk group r, and care state z from an **acutely infected** partner from age group a', risk group r', and care state z' is given by $T_A^{a,r,z,a',r',z'}(j)$:

$$T_A^{a,r,z,a',r',z'}(j) = \begin{cases} (1-\eta^M_{condom} s^{a,r,a',r'}(j)) \times \left(1 - \exp\left[-\beta_M \times d_A(z') \times n^j_M\right]\right), \text{ if } z \in \{0,1,4\} \\ (1-\eta^M_{condom} s^{a,r,a',r'}(j)) \times \sum_{h=0}^{2}\left[\theta_z(h)(1-\eta_{PrEP}(h)) \times \left(1 - \exp\left[-\beta_M \times d_A(z') \times n^j_M\right]\right)\right], \text{ if } z \in \{2,3\} \end{cases} \tag{44}$$

Similarly, the probability of infection to an uninfected MSM in age group a, risk group r, and care state z infected partners from a **chronically infected** partner from age group a', risk group r', CD4 count category x', and care state z', is given by

$T_I^{a,r,z,a',r',x',z'}(j)$:

$$T_I^{a,r,z,a',r',x',z'}(j) = \begin{cases} (1-\eta_{condom}^{M} s^{a,r,a',r'}(j)) \times \left(1-\exp\left[-\beta_M \times d_I(z',x') \times n_M^j\right]\right), & \text{if } z \in \{0,1,4\} \\ (1-\eta_{condom}^{M} s^{a,r,a',r'}(j)) \times \sum_{h=0}^{2} \left[\theta_z(h)(1-\eta_{PrEP}(h)) \times \left(1-\exp\left[-\beta_M \times d_I(z',x') \times n_M^j\right]\right)\right], & \text{if } z \in \{2,3\} \end{cases} \tag{45}$$

The rate of infection to a susceptible individual in age group a, risk group r, and care state z from partnerships of type j with (acutely/chronically) infected individuals in age group a' and risk group r' is given by multiplying the yearly rate of partnership (given by $2c_M^{a,r}(j)\rho^{a,r,a',r'}(j)$) with the probability that their partner is acutely/chronically infected, multiplied by the probability of transmission over the course of the partnership given that their partner is acutely/chronically infected. The force of infection, $\lambda_z^{a,r}$, to an individual in age group a and risk group r is given by summing over all of these rates:

$$\lambda_z^{a,r} = \sum_{j=\text{type of partner}} 2c_M^{a,r}(j) \left[\sum_{a'=0}^{1}\sum_{r'=0}^{1} \rho^{a,r,a',r'}(j) \times \left(\sum_{z'=0}^{6} \frac{A_{z'}^{a',r'}}{N^{a',r'}} \times T_A^{a,r,z,a',r',z'}(j) + \sum_{z'=0}^{9}\sum_{x'=0}^{3} \frac{I_{z'}^{a',r',x'}}{N^{a',r'}} \times T_I^{a,r,z,a',r',x',z'}(j)\right)\right] \tag{46}$$

1.5.7 Transmission to female partners

We let β_F denote the per sex-act probability of HIV transmission and η_{condom}^F denote the efficacy of condom use for vaginal sex acts. Letting $T_A^{F,a',r',z'}(j)$ denote the probability (per partnership per six months) of infection to a female partner of type j from an acutely infected MSM partner from age group a', risk group r', and care state z':

$$T_A^{F,a',r',z'}(j) = (1-\eta_{condom}^F s_F(j)) \times (1-\exp\left[-\beta_F \times d_A(z') \times n_F(j)\right]) \tag{47}$$

and letting $T_I^{F,a',r',x',z'}(j)$ denote the probability per partnership per six months of infection to a female partner of type j from a chronically infected MSM partner from age group a', risk group r', CD4 count x' and care state z':

$$T_I^{F,a',r',x',z'}(j) = (1-\eta_{condom}^F s_F(j)) \times (1-\exp\left[-\beta_F \times d_I(z',x') \times n_F(j)\right]) \tag{48}$$

Then, assuming that female partners are not infected with HIV, the cumulative number of infections to female partners in year t is given by:

$$\frac{d(\text{Cumulative new HIV infections, female partners})}{dt} = \sum_{j=0}^{1}\sum_{a'=0}^{1}\sum_{r'=0}^{1} 2c_F^{a,r}(j) \left(\sum_{z'=0}^{6} A_z'^{a',r'} \times T_A^{F,a',r',z'}(j) + \sum_{z'=0}^{9}\sum_{x'=0}^{3} I_{z'}^{a',r',x'} \times T_I^{F,a',r',x',z'}(j)\right) \tag{49}$$

1.5.8 HIV progression

Within the model, acutely infected individuals not on ART progress to chronic infection at a rate γ_{acute}. A proportion f_x of new entrants to a chronically infected category start in CD4 group x. The CD4 count of chronically infected individuals who are not on ART or are not adherent to ART decreases over time, and without treatment will transition from their initial CD4 category to eventually having a CD4 count of < 200 cells/μl (unless they die before reaching this CD4 category).

Previous studies have shown that higher mortality rates are observed among individuals who initiate ART at lower CD4 counts[47]. Accordingly, we assumed that on ART, an individual's death rate is proportional to the death rate of their CD4 category prior to starting ART, multiplied by a constant scaling factor ν. CD4 progression of individuals on ART is not

modelled explicitly (i.e. they do not progress through CD4 stages), and we make the assumption that individuals stopping ART will quickly return to their CD4 level at their time of ART initiation. Previous modelling studies have shown that the specific assumptions about CD4 progression make little difference to modelled impact of interventions when models are calibrated to prevalence and ART coverage data[48].

Only sampled progression and HIV-related death parameters that give a mean life expectancy without ART of 8.7 – 14.2 years were considered[15]. Letting $\psi_x = \gamma_x/(\gamma_x + \alpha_x + \mu)$, $\psi_{acute} = \gamma_{acute}/(\gamma_{acute} + \mu)$, $\Delta_x = \frac{\alpha_x+\mu}{\alpha_x+\mu+\gamma_x}$, $\Delta_{acute} = \frac{\mu}{\mu+\gamma_{acute}}$ this life expectancy is calculated as:

$$
\begin{aligned}
&\text{Life expectancy without ART} = \\
&f_0 \left\{ \psi_2\psi_1\psi_0\psi_{acute}\left(\frac{1}{\gamma_{acute}} + \sum_{y=0}^{2}\frac{1}{\gamma_y} + \frac{1}{\alpha_3}\right) + (1-\psi_2)\psi_1\psi_0\psi_{acute}\left(\frac{1}{\gamma_{acute}} + \sum_{y=0}^{1}\frac{1}{\gamma_y} + \Delta_2\right) + (1-\psi_1)\psi_0\psi_{acute}\left(\frac{1}{\gamma_{acute}} + \frac{1}{\gamma_0} + \Delta_1\right) \right. \\
&\left. + (1-\psi_0)\psi_{acute}\left(\frac{1}{\gamma_{acute}} + \Delta_0\right) + (1-\psi_{acute})\Delta_{acute} \right\} \\
&+ f_1 \left\{ \psi_2\psi_1\psi_{acute}\left(\frac{1}{\gamma_{acute}} + \sum_{y=1}^{2}\frac{1}{\gamma_y} + \frac{1}{\alpha_3}\right) + (1-\psi_2)\psi_1\psi_{acute}\left(\frac{1}{\gamma_{acute}} + \frac{1}{\gamma_1} + \Delta_2\right) + (1-\psi_1)\psi_{acute}\left(\frac{1}{\gamma_{acute}} + \Delta_1\right) + (1-\psi_{acute})\Delta_{acute} \right\} \\
&+ f_2 \left\{ \psi_2\psi_{acute}\left(\frac{1}{\gamma_{acute}} + \frac{1}{\gamma_2} + \frac{1}{\alpha_3}\right) + (1-\psi_2)\psi_{acute}\left(\frac{1}{\gamma_{acute}} + \Delta_2\right) + (1-\psi_{acute})\Delta_{acute} \right\} \\
&+ f_3 \left\{ \psi_{acute}\left(\frac{1}{\gamma_{acute}} + \frac{1}{\alpha_3}\right) + (1-\psi_{acute})\Delta_{acute} \right\}
\end{aligned}
\tag{50}
$$

1.5.9 PrEP adherence

During the demonstration project, participants could choose either a daily or an on-demand PrEP regimen (specifically a 2-1-1 schedule: two pills the day before a sexual episode, one pill per day during a sexual episode, one pill the day after a sexual episode). At month 6 and month 12 of the demonstration project, blood samples of participants were taken, and levels of Tenofovir Diphosphate (TFV-DP) were measured. The number of pills a participant had taken in the previous week were estimated from the level of TFV-DP present in a sample[49,50]. Accordingly, adherence to PrEP was inferred from an individual's level of TFV-DP.

For our main adherence scenario, daily PrEP users with a level of TFV-DP consistent with 4 or more pills per week ($>$ 800 fmol/punch) were classified as having 'good adherence'. Those with levels of TFV-DP consistent with 2 or 3 pills per week ($400 - 800$ fmol/punch) were classified as having 'partial adherence'. Individuals with a TFV-DP below this level were classified as non-adherent.

For on-demand users, we accounted for the number of pills we would expect that participant to have taken, given their recent sexual activity. This number was calculated from participants' responses to the two questions asked of on-demand PrEP users in the Cotonou demonstration project:

- How many sexual episodes have you had in the past month?
- How many days did each sexual episode last (for the last four sexual episodes)?

We assumed that sexual episodes were distinct (i.e. 2 pills should be taken prior to each sexual episode and 1 pill should be taken after every sexual episode) and that pills were taken evenly throughout the month. Doing so may slightly overestimate the number of pills an on-demand user is required to take, as their first sexual episode may start prior to the beginning of the month, and they may not wait 2 full days between sexual episodes. Letting n denote the number of sexual episodes a participant has had in the past month, and letting h_i denote the number of days episode i lasted for, the number of pills in the past week a participant would be expected to take assuming perfect compliance, P, is calculated as:

$$P = \min\left(\frac{\sum_{i=1}^{n}(2 + h_i + 1)}{4.345}, 7\right) \tag{51}$$

In our main analysis, daily users were classified as having 'good adherence' if they took more than 4/7 of the pills expected

under their regimen (i.e. 4 or more pills a week), and having 'partial adherence' if they took more than 2/7 but less than 4/7 of the pills expected under their regimen (i.e. 2 or 3 pills a week). We made the same assumption for on-demand users: if an on-demand PrEP user had a TFV-DP level consistent with $4/7 \times P$ or more pills per week, we classified an individual as having 'good adherence'. As on-demand users who report few sexual episodes would be expected to take fewer pills than daily users, they may have 'good adherence' without taking 4 pills a week.

If they had a levels of TFV-DP consistent with $2/7 \times P$ to $4/7 \times P$ pills per week, they were classified as having 'partial adherence'. On-demand users with levels of TFV-DP below $2/7 \times P$ were classified as being non-adherent. On-demand users with a TFV-DP measure below the limit of quantification were classified as non-adherent. Individuals who reported no sexual activity over the past month were omitted from adherence estimates.

We made the assumption PrEP is as effective for on-demand users who take 4/7 of their pills expected for full coverage as it is for daily users who take 4/7 pills a week[51]. Under this assumption, for daily and on-demand PrEP users classified as having 'good adherence', PrEP reduces the probability of transmission per partnership by 90-100%. For individuals classified as having 'partial adherence' PrEP reduces probability of transmission per partnership by 56-96%, based on analyses of efficacy for daily PrEP[34].

There are different plausible interpretations of biological adherence levels observed during the demonstration project, regarding whether to classify PrEP users with low but detectable levels of TFV-DP as partially adherent, as they may use PrEP around specific periods of sexual activity, and how to classify on-demand users with low/undetectable levels of TFV-DP but who report no sexual activity. Accordingly, we considered an alternative 'optimistic' adherence scenario, in wich we classified those with low but detectable levels of TFV-DP as 'partially adherent' (on the basis that in practice they may be using PrEP around periods of sexual activity), and we also assumed that all on-demand users who reported no sexual activity in the past month had good adherence. The differences between scenarios in who is categorised as having good adherence, partial adherence, or non-adherence is summarised in Table S9.

To explore uncertainty surrounding whether on-demand PrEP efficacy scales with the proportion of doses taken or the absolute number, we also defined a 'pessimistic' adherence scenario. Under this scenario, we required an on-demand user to have levels of TFV-DP consistent with $7/7 \times P$ pills per week, or have TFV-DP levels consistent with 4 or more pills per week, to be classified as having good adherence. However, inferred adherence levels were very similar to our main scenario, resulting in similar on-demand PrEP effectiveness estimates ($18\% - 34\%$ effectiveness in with pessimistic adherence vs $19\% - 38\%$ in our main scenario). Accordingly, we did not consider the population-level impact or cost-effectiveness of this pessimistic scenario.

Yearly rates of switching between PrEP regimens, and rates of dropping out of PrEP, were informed from the 2020 MSM Cotonou PrEP demonstration project. Participants who continued to attend community and clinical visits were recorded as continuing PrEP, irrespective of their levels of TFV-DP recorded. Participants were only recorded as discontinuing PrEP if they stopped attending demonstration project quarterly visits.

Within the model, individuals may stop taking PrEP if they die for non-HIV related reasons or if they leave the sexually active population. In order to match participant retention within the model for the modelled demonstration project, age-specific drop-out rates, ι_a were adjusted to account for this:

$$\iota_0(\text{demonstration}) = \iota - \mu_0 \tag{52}$$

$$\iota_1(\text{demonstration}) = \iota - \mu_1 - \pi_1 \tag{53}$$

For the PrEP scale-up, we did not adjust for MSM leaving the sexually active population:

$$\iota_0(\text{scale-up}) = \iota - \mu_0 \tag{54}$$

$$\iota_1(\text{scale-up}) = \iota - \mu_1 \tag{55}$$

Table S9: **Differences between main and optimistic adherence scenarios.**

Biological estimate of dosing in demonstration project	Main scenario	Optimistic adherence scenario
For daily PrEP users		
≥ 4 doses a week	Good adherence	Good adherence
2 or 3 doses a week	Partial adherence	Partial adherence
< 2 doses a week	Non-adherence	Partial adherence
TFV-DP below limit of quantification	Non-adherence	Non-adherence
For on-demand PrEP users		
$\geq 4/7$ expected doses taken	Good adherence	Good adherence
Between 2/7 and 4/7 expected doses taken	Partial adherence	Partial adherence
$< 2/7$ expected doses taken	Non-adherence	Partial adherence
TFV-DP below limit of quantification and reported sexual activity in past month	Non-adherence	Non-adherence
No reported sexual activity in past month	Omitted from estimate	Good adherence

1.5.10 HIV testing

We assumed that prior to the introduction of ART in 2002, no MSM test routinely for HIV, including those in care state $z = 1$. Within the model, age-specific testing rates for those testing routinely (i.e. τ_1^a) increased linearly from 2002 until 2015, the year in which the highest access to testing was reported in Benin-wide ESDG MSM surveys 2013-2017[3,4,52]. Similarly, to capture trends in the proportion of 25-50-year-old MSM who never routinely test for HIV, we assumed that the proportion of new MSM who never routinely test through time decreased linearly from 2002 to 2015. 50+year old MSM ($a = 2$) were assumed to test at the same rate as 25-50-year-old MSM. Individuals with CD4 counts less than 200 cells/μl tested at an increased relative rate to the rest of the infected population, $g\tau_1^a$ as a consequence of increased testing due to the onset of AIDS-related symptoms. Accordingly, individuals who do not routinely test with $CD4 < 200$ cells/μl (i.e. those in a states $I_0^{a,r,3}$) also tested at a rate of $g\tau_1^a$. Those enrolled on either a daily or on-demand PrEP regimen are assumed to test for HIV at a rate of $\tau_{PrEP} = 4$ times per year, as part of their regimen. Specifically, yearly testing rates for undiagnosed susceptible or acutely infected individuals are given by:

$$\tau_z^a = \begin{cases} 0 \text{ if } z = 0 \\ \tau_1^a \text{ if } z \in \{1, 4\} \\ \tau_{PrEP} \text{ if } z \in \{2, 3\} \end{cases} \tag{56}$$

While yearly testing rates for chronically infected individuals are given by:

$$\tau_z^{a,x} = \begin{cases} 0 \text{ if } z = 0 \text{ and } x \in \{0, 1, 2\} \\ \tau_1^a \text{ if } z \in \{1, 4\} \text{ and } x \in \{0, 1, 2\} \\ g\tau_1^a \text{ if } z \in \{0, 1, 4\} \text{ and } x = 3 \\ \tau_{PrEP} \text{ if } z \in \{2, 3\} \end{cases} \tag{57}$$

1.5.11 ART eligibility

National guidelines for who is offered ART in Benin have changed through time, with ART being offered to those with CD4 counts ≤ 200 since 2002, ≤ 350 since 2012, ≤ 500 since 2015, and for all CD4 counts since 2016. These guidelines are captured within our model. Prior to 2002, $\xi_x = 0$ for $x \in \{\text{acute}, 0, 1, 2, 3\}$. From 2002 onwards $\xi_3 = \xi$, from 2012 onwards $\xi_2 = \xi$, from 2015 onwards $\xi_1 = \xi$, and from 2016 onwards $\xi_{acute} = \xi$ and $\xi_0 = \xi$.

1.6 Cost-effectiveness details

1.6.1 Costs associated with HIV infection

Our cost-effectiveness analysis incorporated three costs associated with HIV infection: i) the cost of individuals initiating ART, ii) the ongoing cost of ART, and iii) other costs associated with HIV infection for individuals not on ART. ART costs i) and ii) were informed by the costs estimated from an analysis of ART costs during a demonstration project of treatment as prevention (TaSP) for FSW in Cotonou, Benin[40]. The cost of ART initiations in year t, $K_{ART}^{initiation}(t)$, is given by:

$$K_{ART}^{initiation}(t) = C_{ART}^{initiation} \int_{t-1}^{t} \sum_{a=0}^{2} \sum_{r=0}^{1} (\xi N_5^{a,r} + \zeta N_9^{a,r})\, dt \tag{58}$$

while the ongoing cost of ART treatment in year t, $K_{ART}^{ongoing}$ is given by:

$$K_{ART}^{ongoing} = C_{ART}^{ongoing} \int_{t-1}^{t} \sum_{a=0}^{2} \sum_{r=0}^{1} \sum_{z=5}^{8} N_z^{a,r}(t) dt \tag{59}$$

We calculate the 'other' healthcare cost per person year infected but not on ART, ($C_{other}^{ongoing}$), which satisfy a lifetime cost of HIV care of at least \$3619[41], for a typical infected individual in each realisation of the model. To do so , we run our model from 2020, with one initial acutely infected individual, no susceptible individuals, and no entry into the population, for 100 years for each parameter set. Doing so, we obtain an estimate of an individual's expected number of life years not on ART (L_{notART}), an individual's expected number of initiations onto ART ($n_{ART}^{initiation}$), and an individual's expected number of life years on ART (L_{ART}). The lifetime cost of HIV care is then given by:

$$C_{lifetime} = C_{other}^{ongoing} L_{notART} + C_{ART}^{initiation} n_{ART}^{initiation} + C_{ART}^{ongoing} L_{ART} \tag{60}$$

To obtain a lifetime cost of HIV care at least \$3619, we set:

$$C_{other}^{ongoing} = \max\left(0, \frac{3619 - C_{ART}^{initiation} n_{ART}^{initiation} - C_{ART}^{ongoing} L_{ART}}{L_{notART}}\right) \tag{61}$$

Doing so, other costs of HIV care ranged from 0 per person per year (when lifetime costs of ART alone cost above \$3619) to \$267 per person per year infected but not on ART.

1.6.2 Costs associated with PrEP

Our cost-effectiveness analysis incorporated four costs associated with PrEP use: i) costs of initiation onto PrEP, ii) ongoing PrEP drug costs, iii) ongoing non-drug costs associated with PrEP use, and iv) confirmatory HIV tests for individuals on a PrEP regimen who acquire HIV. The cost of PrEP initiations in year t, $K_{PrEP}^{initiation}$ is given by:

$$K_{PrEP}^{initiation} = C_{PrEP}^{initiation} \int_{t-1}^{t} \sum_{a=0}^{1} \sum_{r=0}^{1} (\omega_2 + \omega_3)\,(\tau_1^a(t) S_1^{a,r}(t) + \tau_4^a(t) S_4^{a,r}(t))\, dt \tag{62}$$

the ongoing non-drug cost of PrEP in year t, $K_{PrEP}^{nondrug}$ is given by:

Table S10: **Differences in costing assumptions for the 1-year PrEP demonstration project and PrEP scale-up.** Costing assumptions for the 1-year PrEP demonstration project are based upon the services provided and costs incurred during the 2020-21 Cotonou MSM PrEP demonstration project. Costing assumptions for the PrEP scale-up are based on differences between the services provided between the demonstration project and national PrEP scale-up programs from 2022 (Personal Communication, Carin Ahouada, Plan International Bénin), and differences in PrEP drug costs between 2020 and 2022.

Cost-relevant quantity	Unit cost ($US2021)	1-year demonstration project	PrEP scale-up
Hepatitis B vaccination	10.49	Yes, for all participants:<br>- at initiation	No
Screening and treatment of other STIs	6.96	Yes, for all participants:<br>- at initiation	No
Laboratory test, transaminases	5.53 per test<br>(0.38 per participant)	Yes for $\sim 7\%$ of participants:<br>- at initiation<br>- ongoing (every six months)	No
Laboratory test, creatinine	2.77 per test	Yes, for all participants:<br>- at initiation<br>- ongoing (every six months)	Yes, for all participants:<br>- at initiation<br>- ongoing (every six months)
HIV self-tests	3.95 per test	Yes, for all participants<br>- three months after initiation<br>- ongoing (every six months), alternating with standard tests to test participants every three months)	Yes, for all participants<br>- three months after initiation<br>- ongoing (every six months), alternating with standard tests to test participants every three months)
Standard HIV screening tests	2.64 per test	Yes, for all participants<br>- at initiation<br>- ongoing (every six months, alternating with self-tests to test participants every three months)	Yes, for all participants<br>- at initiation<br>- ongoing (every six months, alternating with self-tests to test participants every three months)
Confirmatory HIV tests	4.80 per test	Yes, for all participants who test positive to a self-test or standard HIV test	Yes, for all participants who test positive to a self-test or standard HIV test
Boxes of TDF/FTC (30 doses per box)	1-year demonstration: 7.90 per box<br>PrEP scale-up: 3.00 per box	Yes,<br>Daily users - 16.1 boxes per participant per year<br>On-demand users - 4.1 boxes per participant per year	Yes,<br>Daily users - 16.1 boxes per participant per year<br>On-demand users - 4.1 boxes per participant per year
Community facilitator annual salary	2213.10 per facilitator per year	Yes, included in ongoing costs<br>10 community facilitators per 204 participants	Yes, included in ongoing costs<br>4 community facilitators per 325 participants (in line with national programmatic targets, Carin Ahouada, Plan International Bénin, personal communication).
Travel costs per facilitator	476.10 per facilitator per year	Yes, included in ongoing costs	Yes, included in ongoing costs
Training costs per facilitator	990.30 per facilitator	Yes, included in initiation costs	Yes, included in ongoing costs, annualised over two years

Abbreviations. PrEP: pre-exposure prophylaxis, STI: sexually transmitted infection

$$K_{PrEP}^{nondrug} = C_{PrEP}^{nondrug} \int_{t-1}^{t} \sum_{a=0}^{1} \sum_{r=0}^{1} \sum_{z=2}^{3} N_z^{a,r}(t)dt \tag{63}$$

The ongoing drug costs of PrEP in year t, taking into account different numbers of doses given to daily and on-demand users, is given by:

$$K_{PrEP}^{drug} = \int_{t-1}^{t} \sum_{a=0}^{1} \sum_{r=0}^{1} \left(C_{PrEP}^{daily} N_2^{a,r} + C_{PrEP}^{on-demand} N_3^{a,r} \right) dt \tag{64}$$

The costs of confirmatory HIV tests for individuals on PrEP who have tested positive for HIV in year t is given by:

$$K_{conftest} = C_{conftest} \int_{t-1}^{t} \sum_{a=0}^{1} \sum_{r=0}^{1} \sum_{z=2}^{3} \left(\tau_z^a A_z^{a,r} + \sum_{x=0}^{3} \tau_z^{a,x} I_z^{a,r,x} \right) dt \tag{65}$$

The excess costs incurred by a PrEP intervention until year t, discounted at a yearly rate of D_{cost} from the year the intervention begins ($t_{intervention}$) is given by:

$$\begin{aligned} &\text{Excess costs until year t} = \qquad (66) \\ &\sum_{t_{intervention}}^{t} (1 - D_{cost})^{t - t_{intervention}} \times \Big[\Big(K_{ART}^{initiation}(t|\text{intervention}) + K_{ART}^{ongoing}(t|\text{intervention}) + K_{other}^{ongoing}(t|\text{intervention}) \\ &+ K_{PrEP}^{initiation}(t|\text{intervention}) + K_{PrEP}^{nondrug}(t|\text{intervention}) + K_{PrEP}^{drug}(t|\text{intervention} + K_{conftest}(t|\text{intervention}) \Big) \\ &- \Big(K_{ART}^{initiation}(t|\text{baseline}) + K_{ART}^{ongoing}(t|\text{baseline}) + K_{other}^{ongoing}(t|\text{baseline}) \Big) \Big] \end{aligned}$$

1.6.3 Calculating DALYs

To estimate the number of Disability Adjusted Life Years (DALYs) averted by a PrEP intervention, we compared both the years of life lost (YLL) from early death and the years of life lived with disability (YLD) that result from a PrEP based intervention to a scenario where PrEP is not introduced.

YLL from HIV-related deaths in year t can be calculated by:

$$YLL_\alpha = \int_{t-1}^{t} \sum_{a=0}^{2} \sum_{r=0}^{1} \sum_{x=0}^{3} \left(\sum_{z=0}^{5} \alpha_x l_a I_z^{a,r,x}(t) + \sum_{z=8}^{9} \alpha_x l_a I_z^{a,r,x}(t) + \nu \sum_{z=6}^{7} \alpha_x l_a I_z^{a,r,x}(t) \right) dt \tag{67}$$

However, if an intervention reduces the number of HIV-related deaths, then the number of non-HIV related deaths will increase (by a small amount). Without taking this into account, we would overestimate the YLL associated with a HIV intervention. Accordingly, we also calculated YLL associated with non-HIV related deaths:

$$YLL_\mu = \int_{t-1}^{t} \sum_{a=0}^{2} l_a \mu_a N_a(t) dt \tag{68}$$

The YLL averted by a PrEP intervention in year t is then given by:

$$\text{YLL averted in year } t = (YLL_\alpha(t|\text{baseline}) + YLL_\mu(t|\text{baseline})) - (YLL_\alpha(t|\text{intervention}) + YLL_\mu(t|\text{intervention})) \tag{69}$$

YLD are calculated by multiplying life years lived with HIV with disability weightings corresponding to different stages of HIV[42]:

$$
\begin{aligned}
YLD(t) = W_{early} \int_{t-1}^{t} \sum_{a=0}^{2} \sum_{r=0}^{1} \left(\sum_{z=0}^{5} A_z^{a,r}(t) + \sum_{z=0}^{5} I_z^{a,r,0}(t) + \sum_{z=8}^{9} I_z^{a,r,0}(t) \right) dt \\
+ W_{mid} \int_{t-1}^{t} \sum_{a=0}^{2} \sum_{r=0}^{1} \sum_{x=1}^{2} \left(\sum_{z=0}^{5} I_z^{a,r,x}(t) + \sum_{z=8}^{9} I_z^{a,r,x}(t) \right) dt \\
+ W_{late} \int_{t-1}^{t} \sum_{a=0}^{2} \sum_{r=0}^{1} \left(\sum_{z=0}^{5} I_z^{a,r,3}(t) + \sum_{z=8}^{9} I_z^{a,r,3}(t) \right) dt \\
+ W_{ART} \int_{t-1}^{t} \sum_{a=0}^{2} \sum_{r=0}^{1} \left(A_6^{a,r}(t) + \sum_{z=6}^{7} \sum_{x=0}^{3} I_z^{a,r,x}(t) \right) dt
\end{aligned} \tag{70}
$$

The number of DALYs averted until year t, discounted at a yearly rate of D_{DALY} from the year the intervention begins ($t_{intervention}$) is then given by:

$$
\begin{aligned}
\text{DALYs averted until year t} = \sum_{t_{intervention}}^{t} (1 - D_{DALY})^{t - t_{intervention}} \times [(YLL_\alpha(t|\text{baseline}) + YLL_\mu(t|\text{baseline}) + YLD(t|\text{baseline}) \\
-(YLL_\alpha(t|\text{intervention}) + YLL_\mu(t|\text{intervention}) + YLD(t|\text{intervention}))]
\end{aligned} \tag{71}
$$

1.6.4 DALYs and costs for female partners of MSM

By calculating i) the average care cost averted per infection averted among MSM, and ii) the average number of DALYs averted per infection averted among MSM, and extrapolating these ratios to the infections averted among female partners of MSM, we obtained estimates of care costs averted and DALYs averted from averting HIV infections to female partners of MSM.

1.6.5 Threshold analysis

We conducted a cost effectiveness threshold analyses, estimating the percentage change in PrEP costs (scaling $C_{PrEP}^{initiation}$, $C_{PrEP}^{ongoing}$ C_{PrEP}^{daily}, and $C_{PrEP}^{ondemand}$ by a constant factor) such that the cost per DALY averted over 20 years from a PrEP scale-up would be $\leq$ \$1225.

1.7 Model outputs

Table S11: **Summary of model outputs.**

Model output	Symbol	Formula
Fitting outcomes		
Size of sexually active MSM population in year t	$N(t)$	$N(t) = \sum_{a=0}^{1} \sum_{r=0}^{1} \left(\sum_{z=0}^{4} S_z^{a,r}(t) + \sum_{z=0}^{6} A_z^{a,r}(t) + \sum_{z=0}^{9} \sum_{x=0}^{3} I_z^{a,r,x}(t) \right)$
Proportion of sexually active MSM 18-24 years in year t	$m_0(t)$	$N_0(t)/N(t)$
HIV prevalence MSM in age group a in year t	$m_{prev}^a(t)$	$\frac{\sum_{r=0}^{1}\left(\sum_{z=0}^{6} A_z^{a,r}(t)+\sum_{z=0}^{9}\sum_{x=0}^{3} I_z^{a,r,x}(t)\right)}{\sum_{r=0}^{1}\left(\sum_{z=0}^{4} S_z^{a,r}(t)+\sum_{z=0}^{6} A_z^{a,r}(t)+\sum_{z=0}^{9}\sum_{x=0}^{3} I_z^{a,r,x}(t)\right)}$
Cumulative HIV infections among MSM in year t		$\int_{t-1}^{t} \sum_{a=0}^{1} \sum_{r=0}^{1} \sum_{z=0}^{4} \lambda_z^{a,r}(t) S_z^{a,r}(t) dt$
HIV incidence in year t	$m_{inc}(t)$	$\frac{\text{Cumulative HIV infections in year } t}{\int_{t-1}^{t} N(t)dt}$
Proportion of sexually active HIV positive MSM who are on ART	$m_{ART}(t)$	$\frac{\sum_{a=0}^{1}\sum_{r=0}^{1}\left(A_6^{a,r}(t)+\sum_{z=6}^{9}\sum_{x=0}^{3} I_z^{a,r,x}(t)\right)}{\sum_{a=0}^{1}\sum_{r=0}^{1}\left(\sum_{z=0}^{6} A_z^{a,r}(t)+\sum_{z=0}^{9}\sum_{x=0}^{3} I_z^{a,r,x}(t)\right)}$
Proportion of those on ART who are virally suppressed	$m_{supp}(t)$	$\frac{\sum_{a=0}^{1}\sum_{r=0}^{1}\left(\sum_{x=0}^{3} I_7^{a,r,x}(t)\right)}{\sum_{a=0}^{1}\sum_{r=0}^{1}\left(A_6^{a,r}(t)+\sum_{z=6}^{9}\sum_{x=0}^{3} I_z^{a,r,x}(t)\right)}$
Proportion of 25-50-year-old MSM not routinely testing for HIV	$m_{nevertest}(t)$	$\frac{\sum_{r=0}^{1}\left(S_0^{1,r}(t)+A_0^{1,r}(t)+\sum_{x=0}^{3} I_0^{1,r,x}(t)\right)}{\sum_{r=0}^{1}\left(\sum_{z=0}^{4} S_z^{1,r}(t)+\sum_{z=0}^{6} A_z^{1,r}(t)+\sum_{z=0}^{9}\sum_{x=0}^{3} I_z^{1,r,x}(t)\right)}$
DALYs		
Years lost to disease in year t	$YLD(t)$	$W_{early} \int_{t-1}^{t} \sum_{a=0}^{2} \sum_{r=0}^{1} \left(A_5^{a,r}(t) + \sum_{z=0}^{5} I_z^{a,r,0}(t) + \sum_{z=8}^{9} I_z^{a,r,0}(t) \right) dt$ $+W_{mid} \int_{t-1}^{t} \sum_{a=0}^{2} \sum_{r=0}^{1} \sum_{x=1}^{2} \left(\sum_{z=0}^{5} I_z^{a,r,x}(t) + \sum_{z=8}^{9} I_z^{a,r,x}(t) \right) dt$ $+W_{late} \int_{t-1}^{t} \sum_{a=0}^{2} \sum_{r=0}^{1} \left(\sum_{z=0}^{5} I_z^{a,r,3}(t) + \sum_{z=8}^{9} I_z^{a,r,3}(t) \right) dt$ $+W_{ART} \int_{t-1}^{t} \sum_{a=0}^{2} \sum_{r=0}^{1} \left(A_6^{a,r}(t) + \sum_{z=6}^{7} \sum_{x=0}^{3} I_z^{a,r,x}(t) \right) dt$
Years life lost from HIV related deaths in year t	$YLL_\alpha(t)$	$\int_{t-1}^{t} \sum_{a=0}^{2} \sum_{r=0}^{1} \sum_{x=0}^{3} \left(\sum_{z=0}^{5} \alpha_x l_a I_z^{a,r,x}(t) + \sum_{z=8}^{9} \alpha_x l_a I_z^{a,r,x}(t) \right) dt$ $+\nu \int_{t-1}^{t} \sum_{a=0}^{2} \sum_{r=0}^{1} \sum_{x=0}^{3} \sum_{z=6}^{7} \alpha_x l_a I_z^{a,r,x}(t) dt$
Years life lost from non-HIV related deaths in year t*	$YLL_\mu(t)$	$\int_{t-1}^{t} \sum_{a=0}^{2} l_a \mu_a N_a(t) dt$
Costs		
PrEP initiation costs in year t	$K_{initiation}^{PrEP}$	$C_{PrEP}^{initiation} \int_{t-1}^{t} \sum_{a=0}^{1} \sum_{r=0}^{1} (\omega_2 + \omega_3) \tau_1^a(t) \left(S_1^{a,r}(t) + S_4^{a,r}(t)\right) dt$
Ongoing (non-drug) PrEP costs in year t	$K_{PrEP}^{nondrug}$	$C_{PrEP}^{nondrug} \int_{t-1}^{t} \sum_{a=0}^{1} \sum_{r=0}^{1} \sum_{z=2}^{3} N_z^{a,r}(t) dt$
Ongoing (drug) PrEP costs in year t	K_{PrEP}^{drug}	$\int_{t-1}^{t} \sum_{a=0}^{1} \sum_{r=0}^{1} \left(C_{PrEP}^{daily} N_2^{a,r} + C_{PrEP}^{on-demand} N_3^{a,r} \right) dt$
ART initiation costs in year t	$K_{ART}^{initiation}$	$C_{ART}^{initiation} \int_{t-1}^{t} \sum_{a=0}^{2} \sum_{r=0}^{1} \left(\xi N_5^{a,r} + \zeta N_9^{a,r}\right) dt$
Ongoing ART costs in year t	$K_{ART}^{ongoing}$	$C_{ART}^{ongoing} \int_{t-1}^{t} \sum_{a=0}^{2} \sum_{r=0}^{1} \sum_{z=5}^{8} N_z^{a,r}(t) dt$
Ongoing non-ART costs associated with HIV infection in year t	$K_{other}^{ongoing}$	$C_{other}^{ongoing} \int_{t-1}^{t} \sum_{a=0}^{2} \sum_{r=0}^{1} \left(\sum_{z=0}^{4} A_z^{a,r}(t) + \sum_{z=0}^{4} \sum_{x=0}^{3} I_z^{a,r,x}(t) + \sum_{x=0}^{3} I_9^{a,r,z}(t) \right) dt$
HIV confirmary test costs in year t	$K_{conftest}$	$C_{conftest} \int_{t-1}^{t} \sum_{a=0}^{1} \sum_{r=0}^{1} \sum_{z=2}^{3} \left(\tau_z^a A_z^{a,r} + \sum_{x=0}^{3} \tau_z^{a,x} I_z^{a,r,x} \right) dt$

* Calculated to account for slightly higher non-HIV related deaths in intervention scenario than baseline scenario, to avoid overestimating Years life lost from HIV-related deaths.

Abbreviations. PrEP: pre-exposure prophylaxis, MSM: men who have sex with men, ART: antiretroviral therapy, DALY: disability adjusted life year

1.8 Sensitivity analysis scenarios

Table S12 details sensitivity analysis scenarios for a 5-year and a 20-year PrEP scale-up from 2022, with 30% coverage by 2027, which is presented in Figure 4 of the main paper. the same scenarios are considered for the demonstration, but in this instance demonstration project costs are the main scenario, and scale-up costs are the sensitivity analysis.

Table S12: **Sensitivity scenarios considered** for A) a 5-year and B) a 20-year PrEP scale-up with 30% coverage by 2027.

Modelling assumption	Main scenario	Sensitivity analyses
PrEP adherence	PrEP effectiveness:<br>Daily: 14-23%, On-demand: 19-38%	a) Optimistic adherence, PrEP effectiveness:<br>Daily: 27-40%, On-demand: 38-56%<br>b) Ideal adherence, PrEP effectiveness:<br>Daily: 91-99%, On-demand: 91-99%
PrEP prioritisation	All MSM offered PrEP at testing for HIV	d) Only high-risk MSM offered PrEP at testing
HIV infections among female partners	Included in impact and cost-effectiveness calculations	d) Not included in impact and cost-effectiveness calculations
Intervention evaluation period	20 years (until 2042)	e) 50 years (until 2072)
DALY calculation	As described in methods	g) YLL after the intervention evaluation period not included, as in[53]
Discounting	Costs: 3%, DALYs: 3%	h) Costs: 3%, DALYs: 0%<br>i) Costs: 0%, DALYs: 0%
Lifetime cost of HIV care ($US2021\$$)	3619–5423<br>The lifetime median cost of HIV care from LMICs= \$3619. Higher costs occurred when lifetime ART costs alone exceeded \$3619.	j) Lower lifetime cost of HIV care: 2240 – 5423<br>The lowest lifetime cost of HIV care from LMICs = \$1454. In all model runs, higher lifetime ART costs alone exceeded \$1454, so in this sensitivity analysis we set non-ART costs of HIV care to 0.<br><br>k) Higher lifetime cost of HIV care: 13967<br>The highest lifetime cost of HIV care from LMICs = \$13967
PrEP related costs	Scale-up costs (Table S8)	Demonstration project costs (Table S8)
Fitting outcomes	Calibrated to main fitting outcomes (Table S13)	c) Additionally calibrated to age-specific prevalence (Table S13)

Abbreviations. PrEP: pre-exposure prophylaxis; DALY: disability-adjusted life year; YLL: Years of life lost; LMIC; Lower-middle income country

Separately, we also consider scenarios where we vary coverage and adherence, varying coverage by 2027 from 5 to 50% in 5% intervals, and varying the proportion of PrEP users with good adherence from 10 to 100% in 10% intervals. In these cases, we assume that adherence is the same for both PrEP regimens, and 0% of PrEP users have partial adherence. Results from this are shown in Figure S24.

2 Model fitting

2.1 Fitting outcomes

Table S13: **Fitting outcomes.**

Fitting outcome	Range	Source
Main scenario		
Size of sexually active MSM population	2018: 1696 - 5087	BeSYP (network of Benin LGBTIQ+ associations) mapping ±50% (Previous estimate[54])
	2022: 1770 - 5930	ESDG 2022 (Grand Cotonou), 95% CI (Previous estimate[5])
Proportion of sexually active MSM population who are 18-24 years old	2017: 0.33 - 0.47	ESDG 2017 (Grand Cotonou), 95% CI (Novel analysis)
	2020: 0.37 - 0.51	Cotonou MSM PrEP demo, 95% CI (Novel analysis)
	2022: 0.42 - 0.54	ESDG 2022 (Grand Cotonou), 95% CI (Novel analysis)
HIV prevalence (sexually active MSM)	2013: 0.15 - 0.26	ESDG 2013 (Grand Cotonou), 95% CI (from prevalence in Cotonou and Abomey-Calavi in[3])
	2017: 0.05 - 0.14	ESDG 2017 (Grand Cotonou), 95% CI (Novel analysis)
	2022: 0.09 - 0.18	ESDG 2022 (Grand Cotonou), 95% CI (Novel analysis)
Proportion of sexually active HIV positive MSM who are on ART	2017: 0.215 - 0.56	ESDG 2017 (Benin-wide), 95% CI (Novel analysis)
Proportion of sexually active MSM on ART who are virally suppressed	2017: 0.19 - 0.74	ESDG 2017 (Benin-wide), 95% CI (Novel analysis)
Proportion of 25-50-year-old MSM who have never taken a HIV test	2017: 0.09 - 0.22	ESDG 2017 (Grand Cotonou), 95% CI (Novel analysis)
	2022: 0.01 - 0.07	ESDG 2022 (Grand Cotonou), 95% CI (Novel analysis)
Alternative scenarios		
a) HIV prevalence among 18-24-year-old MSM	2017: 0.065 - 0.25	ESDG 2017 (Grand Cotonou), 95% CI (Novel analysis)
	2022: 0.13 - 0.27	ESDG 2022 (Grand Cotonou), 95% CI (Novel analysis)
b) HIV prevalence among 25-50-year-old MSM	2017: 0.03 - 0.14	ESDG 2017 (Grand Cotonou), 95% CI (Novel analysis)
	2022: 0.03 - 0.10	ESDG 2022 (Grand Cotonou), 95% CI (Novel analysis)

Abbreviations. PrEP: pre-exposure prophylaxis, MSM: men who have sex with men, ART: antiretroviral therapy

2.2 Fitting algorithm

We fitted the model using a Markov Chain Monte Carlo (MCMC) approach. We ran 200,000 'burn-in' iterations of a Metropolis-Hastings algorithm[55], before running 1,300,000 further iterations to generate a sample from the posterior distribution of the model. At each iteration, the model proposes an update to the joint distribution of parameters, with the new candidate parameter set sampled from multivariate Gaussian distribution centred on the previous accepted parameter set and with the variance of each parameter set at 4% of the prior range for each parameter (apart from β_M, which we set at 1%). If any parameter within the parameter set fell outside of the prior range, that specific parameter was resampled until it fell within the prior range. Once a proposal parameter set was obtained with all parameters falling within prior ranges, the model was run and the log likelihood of observing our set of fitting outcomes given the model was evaluated, with acceptance or rejection of a parameter set dependent on this.

The 1,300,000 parameter sets were then thinned to obtain a sample of 1000 posterior parameter sets from the posterior distribution. Convergence of the MCMC chain was assessed by visually inspecting trace plots of each parameter, comparing density plots of the first vs the last 250 sampled parameter sets, and using the Geweke diagnostic[56]. MCMC diagnostics are shown in Section 2.2. Table S14 summarises main impact and cost-effectiveness results with a sample of 500 vs 1000 parameter sets, demonstrating the stability of model outcomes.

Female partnership parameters and cost-effectiveness relevant parameters did not impact the model log likelihood. Accordingly, these parameters were sampled directly from uniformly distributed priors.

2.3 Likelihood function

Where data was available, we compared the observed yearly estimates of HIV prevalence ($X_{prev}(t)/Z_{prev}(t)$), the number of the sexually active MSM population in Grand Cotonou ($Z_{pop}(t)$), the proportion of HIV-positive MSM on ART

Table S14: **Comparing main results from 500 parameter sets vs 1000 parameter sets.**

Intervention	Outcome	Estimate with 500 parameter sets	Estimate with 1000 parameter sets
Demonstration project	% of new HIV infections averted over 1 year among project participants.	21.6% (95%UI: 16.7-26.2%)	21.5% (95%UI: 16.6-26.2%)
Demonstration project	% of new HIV infections averted over 20 years among all MSM and female partners	0.2% (95%UI: 0.1-0.3%)	0.2% (95%UI: 0.1-0.4%)
Demonstration project	Cost per DALY averted over 20 years	\$1761 (95%UI: 646-6147)	\$1772 (95%UI: 680-6156)
PrEP scale-up with 30% coverage by 2027	% of new HIV infections averted over 20 years among all MSM and female partners	3.2% (95%UI: 1.7-4.9%)	3.2% (95% UI: 1.6-4.8%)
PrEP scale-up with 30% coverage by 2027	Cost per DALY averted over 20 years	\$398 (95%UI: 27-2923)	\$388 (95%UI: 36-2792)

$(X_{ART}(t)/Z_{ART}(t))$, the proportion on MSM on ART who are virally suppressed $(X_{supp}(t)/Z_{supp}(t))$, the proportion of 25-50-year-old MSM who have never tested for HIV $X_{nevertest}(t)/Z_{nevertest}(t)$, and the proportion of sexually active MSM who are 18-24 years old $(X_{young}(t)/Z_{young}(t))$, against modelled values of each from our model $(m_{prev}(t), m_{inc}(t), m_{ART}(t), m_{supp}(t), m_{nevertest}(t), N(t)$, and $m_0(t))$. Letting $LL_{Binom}(X|Z,m)$ denote the log of the binomial probability mass function, and $LL_{Norm}(Z|N,\sigma^2)$ denote the log of the normal probability density function, the log likelihood of the model is given by:

$$\begin{aligned} LL(\text{parameter set}) = &\sum_{t\in\{2020,2022\}} LL_{Norm}(Z_{pop}(t)|N(t),\sigma^2_{pop}(t)) + \sum_{t\in\{2013,2017,2022\}} LL_{Binom}(X_{prev}(t)|Z_{prev}(t),m_{prev}(t)) \\ &+ \sum_{t\in\{2017,2020,2022\}} LL_{Binom}(X_{young}(t)|Z_{young}(t),m_0(t)) \\ &+ \sum_{t\in\{2017,2022\}} LL_{Binom}(X_{nevertest}(t)|Z_{nevertest}(t),m_{nevertest}(t)) \\ &+ LL_{Binom}(X_{ART}(2017)|Z_{ART}(2017),m_{ART}(2017)) + LL_{Binom}(X_{supp}(2017)|Z_{supp}(2017),m_{supp}(2017)) \end{aligned} \tag{72}$$

2.4 Model posteriors

Supplementary Figures S5 to S11 display density plots of the 1000 posterior parameter sets obtained from the posterior distribution of the model. For the majority of model parameters, the model fitting process returns the prior distribution of those parameters, i.e. our fitting outcomes data was uninformative over the priors for the majority of parameters. However, our model was informative over a number of parameters, in particular: initial population size (N_{init}), the yearly rate of moving from 25-50 year population into the 50+-year-old sexually inactive population (π_1), the proportion of MSM never routinely testing in 2002 ($p(2002)$) and after 2015 ($p(2015)$), probability of transmission per anal sex act (β_M), the yearly rate of initiating ART when eligible (ϕ), the proportion of those starting ART who are adherent, and condom efficacy (η_M^{condom}).

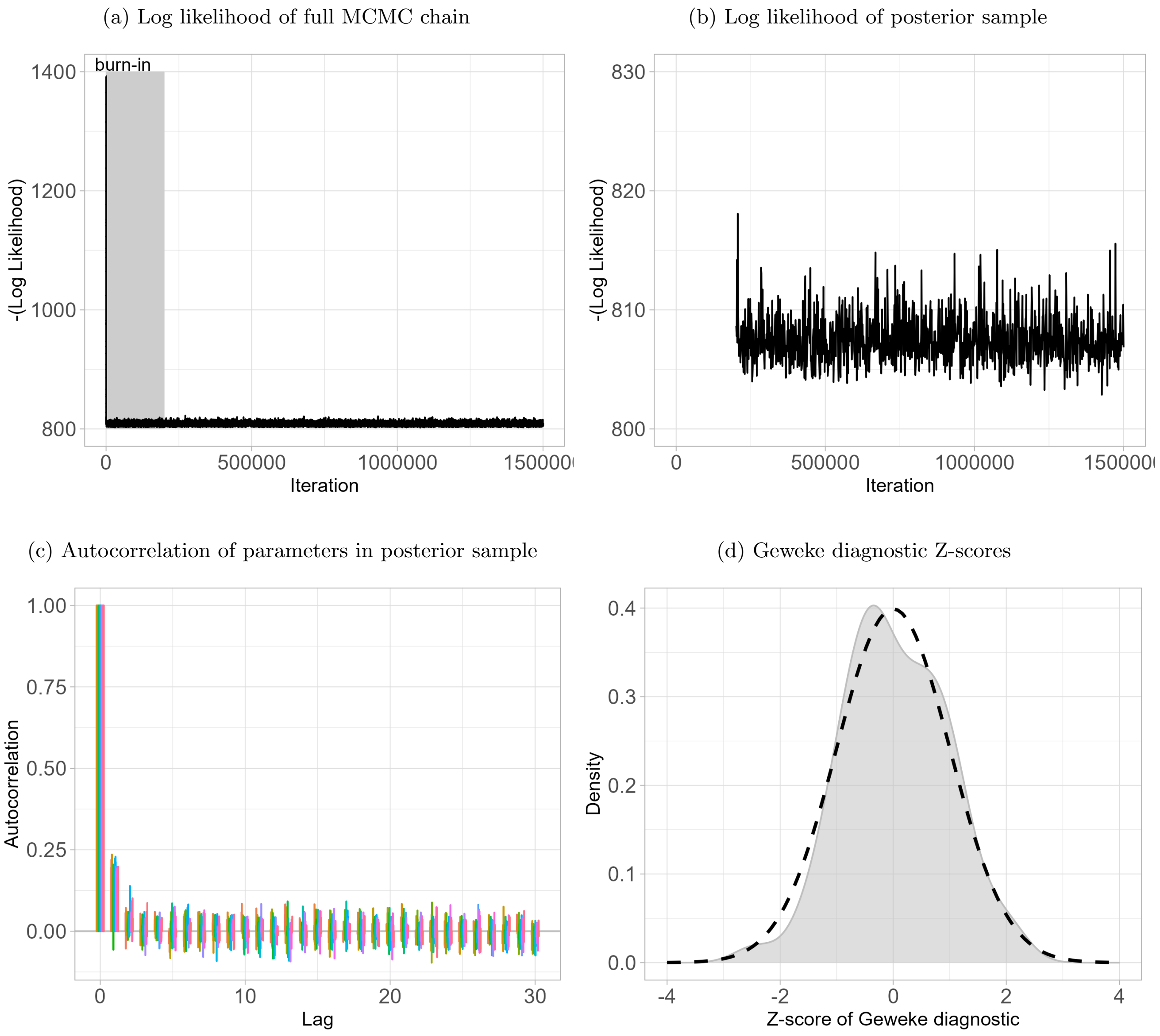

Figure S4: **MCMC trace plots and diagnostics.** a) and b): trace plots of $-1\times$ the model log-likelihood, for a) 1,500,000 iterations of a Metropolis-Hastings algorithm, and b) the 1,000 posterior parameter sets used to generate model results. c) Auto-correlation plots of model parameters from the sample of 1,000 posterior parameter sets - each colour represents a different parameter. d) The density plot of z-score of the Geweke diagnostic for each model parameter from the sample of 1,000 posterior parameter sets, from comparing the first and last 250 sampled parameter sets. For an MCMC chain that has converged, the density plot of Geweke diagnostic Z-scores should approximately resemble the standard normal distribution (shown by the dotted line).

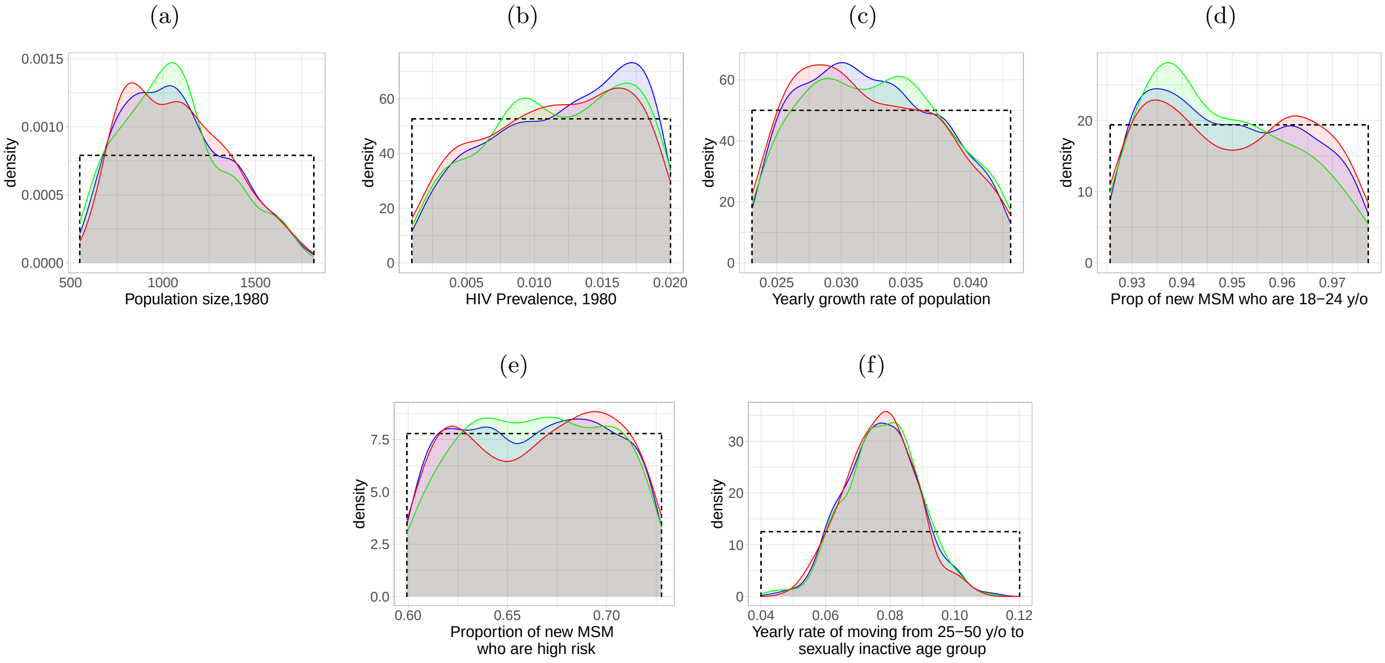

Figure S5: **Posterior distribution density plots for demographic and initial condition parameters.** Posterior parameter density plots of 1000 sampled parameter sets (blue), for parameters relevant to demography and initial conditions. Density plots of the first and last 250 parameter sets are shown in red and green respectively, and dotted lines show model priors.

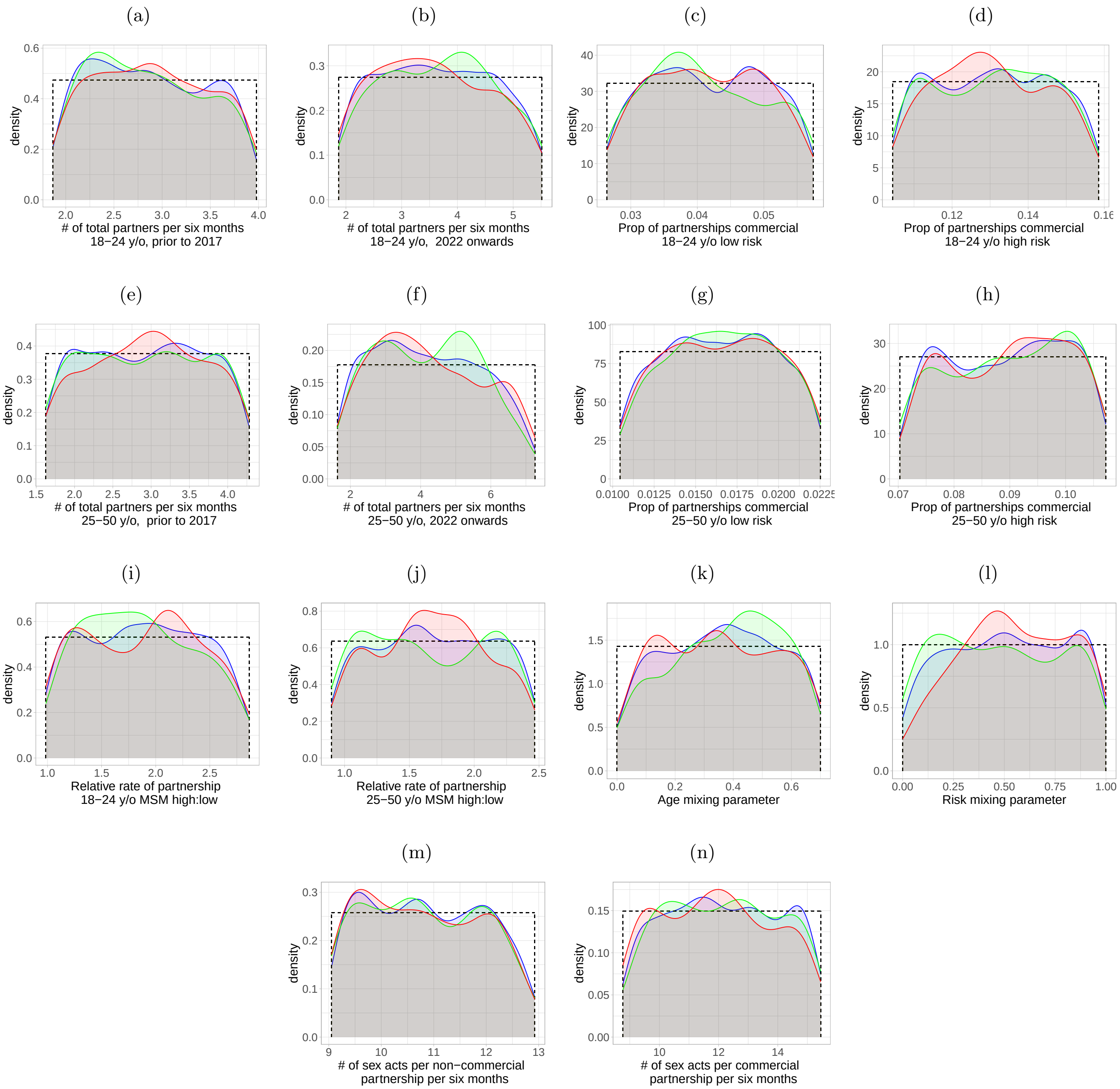

Figure S6: **Posterior distribution density plots for sexual behaviour parameters (partnerships).** Posterior parameter density plots of 1000 sampled parameter sets (blue), for parameters relevant to sexual behaviour with MSM partners (number of partnerships and number of sex acts). Density plots of the first and last 250 parameter sets are shown in red and green respectively, and dotted lines show model priors.

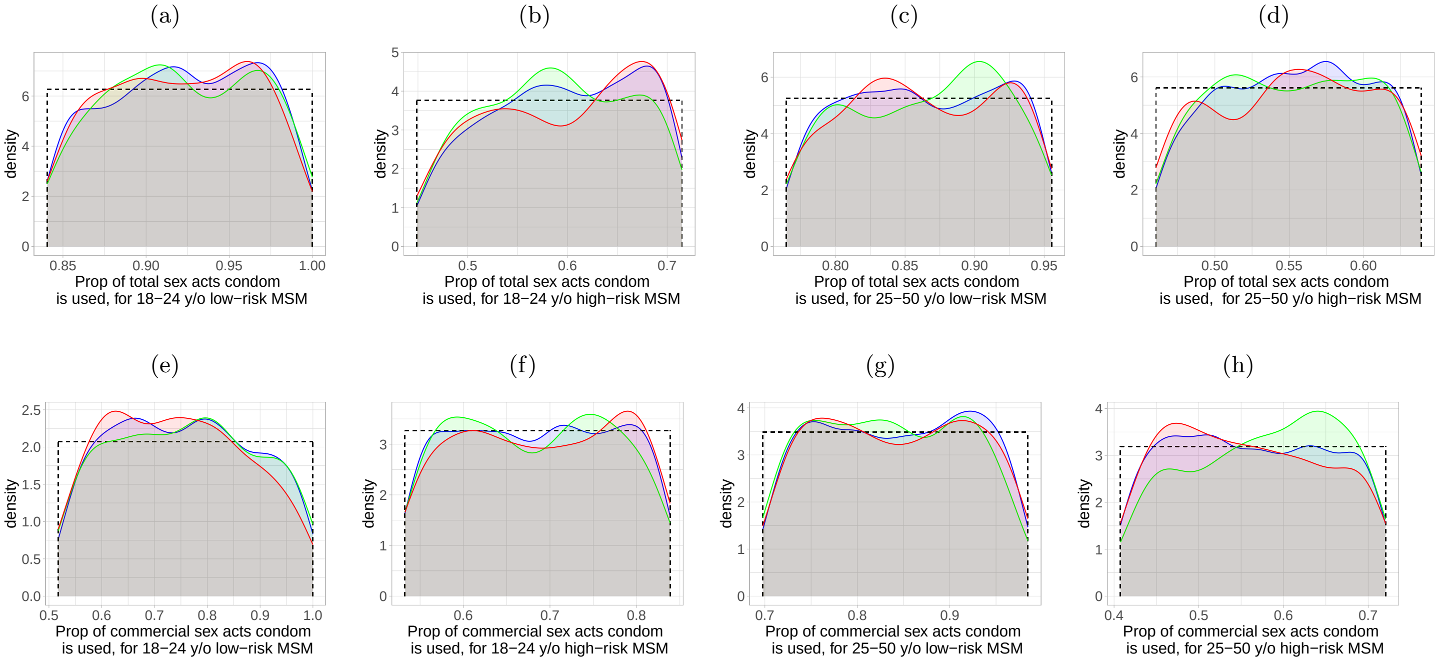

Figure S7: **Posterior distribution density plots for sexual behaviour parameters (condom use).** Posterior parameter density plots of 1000 sampled parameter sets (blue), for parameters relevant to condom use with MSM partners. Density plots of the first and last 250 parameter sets are shown in red and green respectively, and dotted lines show model priors.

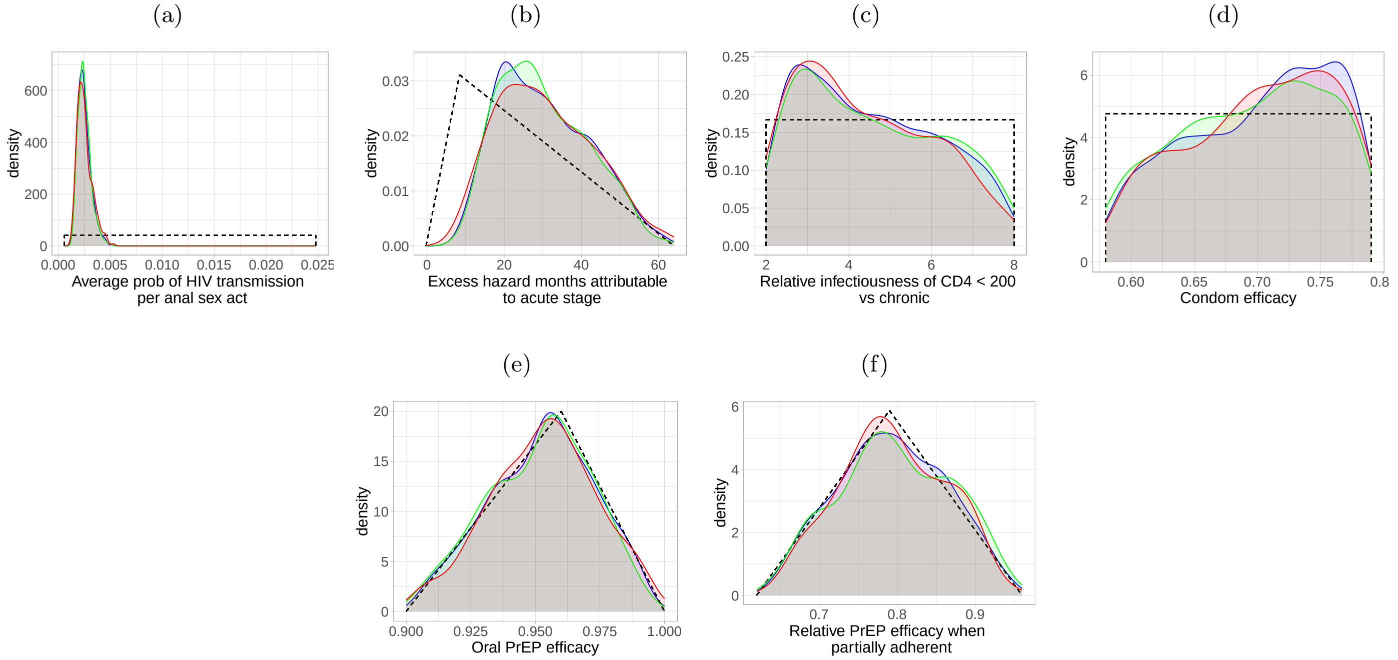

Figure S8: **Posterior distribution density plots for HIV transmission related parameters.** Posterior parameter density plots of 1000 sampled parameter sets (blue), for parameters relevant to HIV transmission. Density plots of the first and last 250 parameter sets are shown in red and green respectively, and dotted lines show model priors.

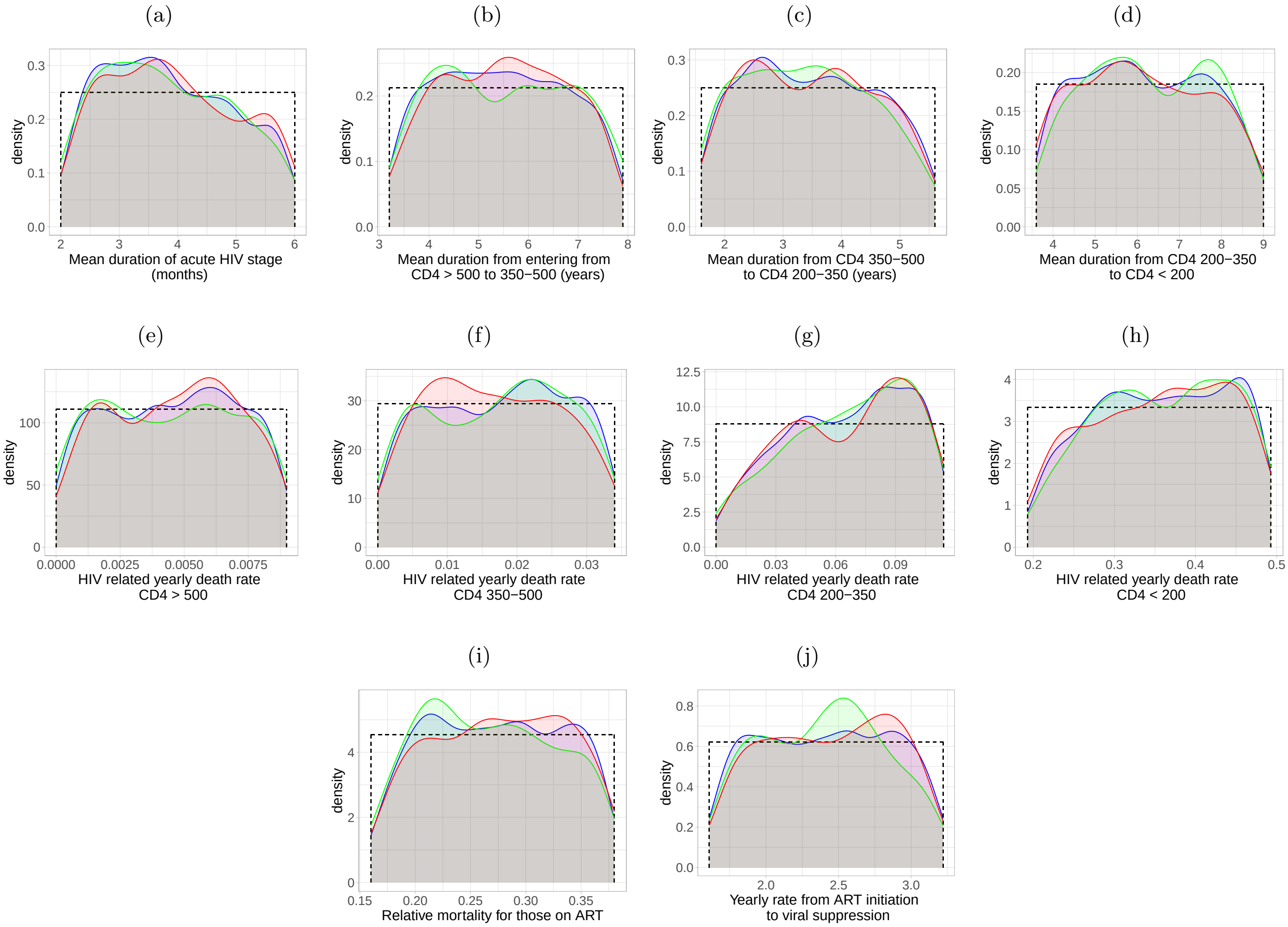

Figure S9: **Posterior distribution density plots for HIV progression related parameters.** Posterior parameter density plots of 1000 sampled parameter sets (blue), for parameters relevant to HIV progression. Density plots of the first and last 250 parameter sets are shown in red and green respectively, and dotted lines show model priors.

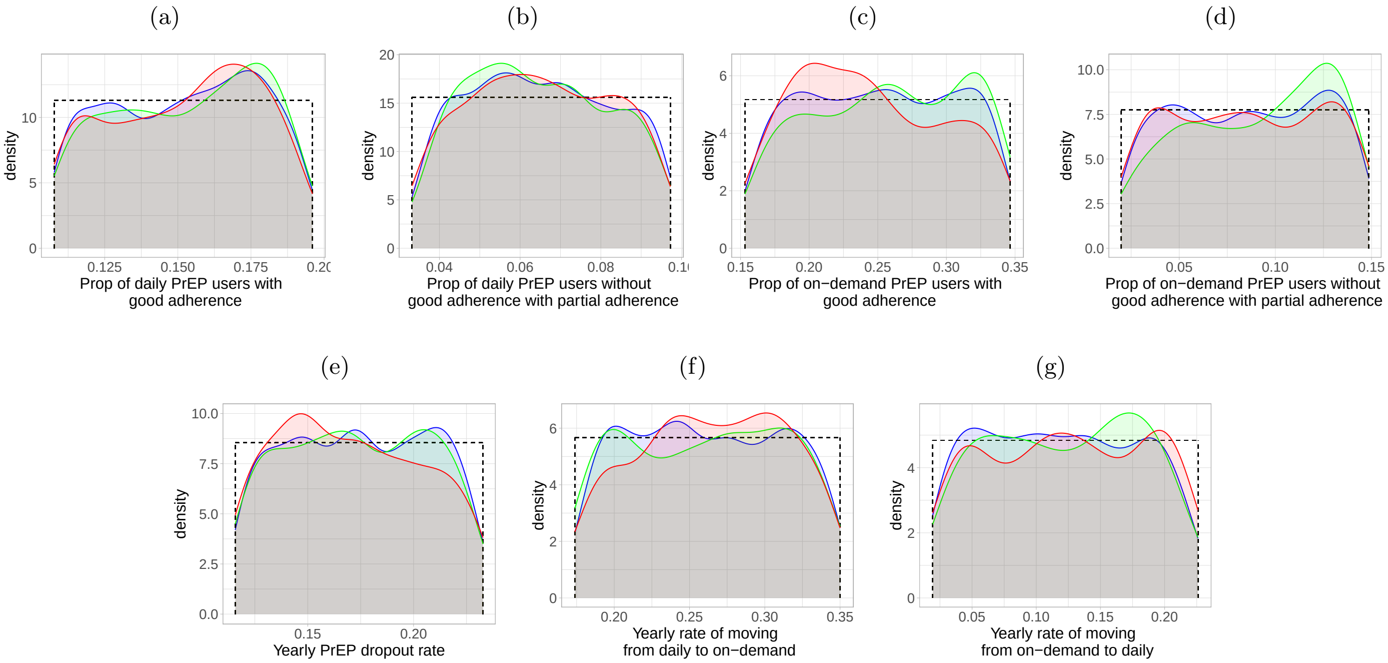

Figure S10: **Posterior distribution density plots for PrEP behaviour related parameters.** Posterior parameter density plots of 1000 sampled parameter sets (blue), for behavioural parameters relevant to PrEP. Density plots of the first and last 250 parameter sets are shown in red and green respectively, and dotted lines show model priors.

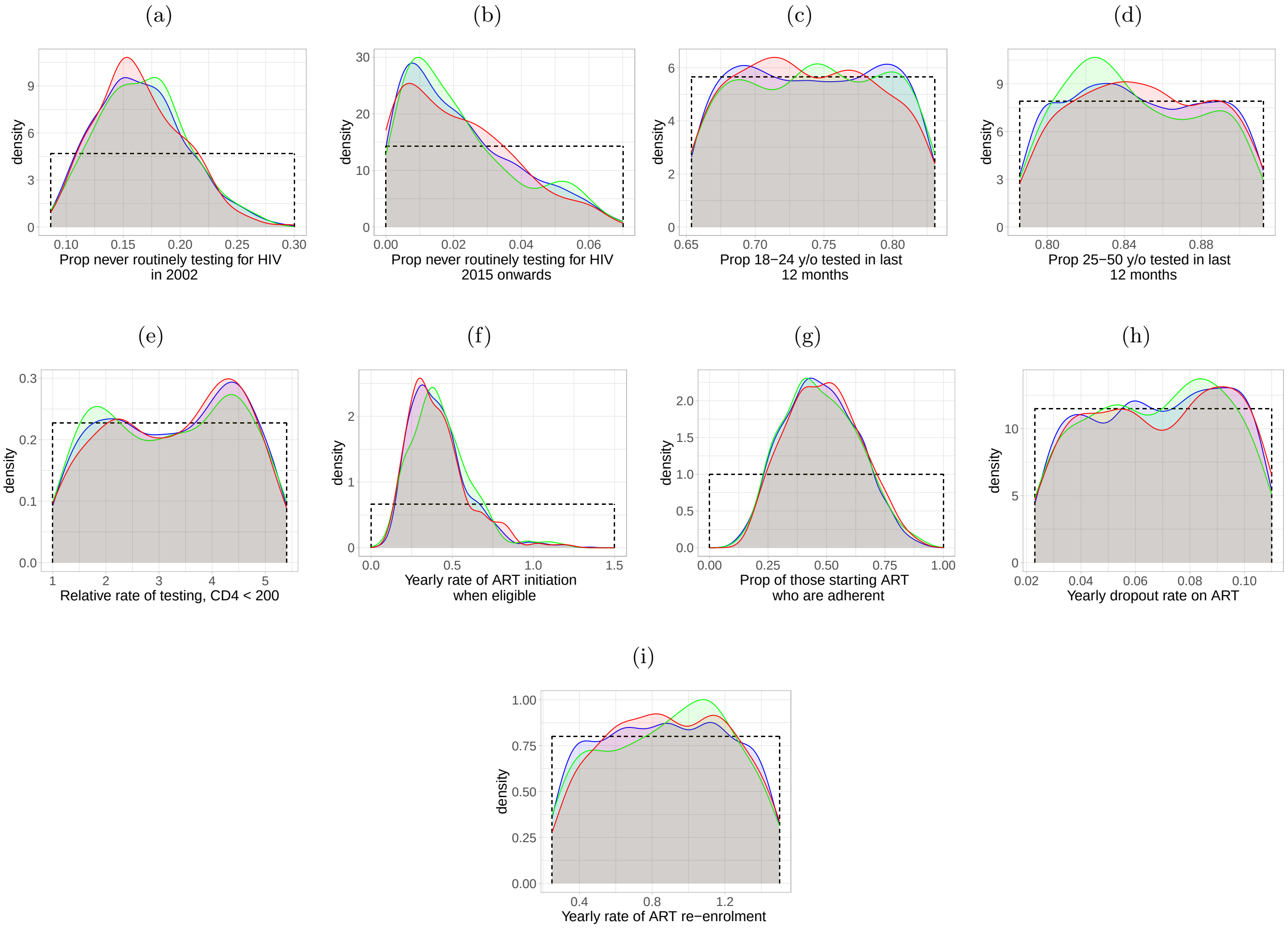

Figure S11: **Posterior distribution density plots for HIV testing and ART behavioural parameters.** Posterior parameter density plots of 1000 sampled parameter sets (blue), for parameters relevant to HIV testing and ART. Density plots of the first and last 250 parameter sets are shown in red and green respectively, and dotted lines show model priors.

3 Supplementary Results

(a) 18-24-year-old MSM (b) 25-50-year-old MSM

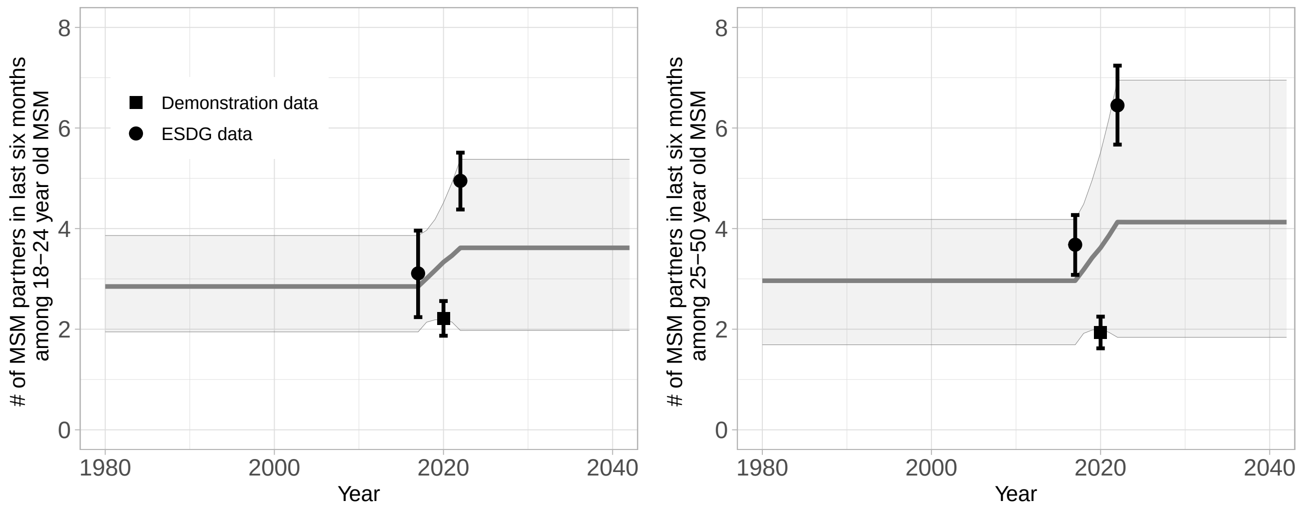

Figure S12: **Trends in MSM partners in the fitted model.** Trends in the modelled average number of MSM partners per 6 months for a) 18–24-year-old MSM and b) 25–50-year-old MSM. Markers (circles for ESDG data, squares for demonstration project data) denote point estimates of the number of MSM partners survey respondents reported in the last 6 months. Cross-bars show 95% confidence intervals. In both panels, solid lines and shaded ribbons represent median model predictions and 95% uncertainty intervals from 1000 posterior parameter sets.

(a) Overall trends (b) Trends by risk

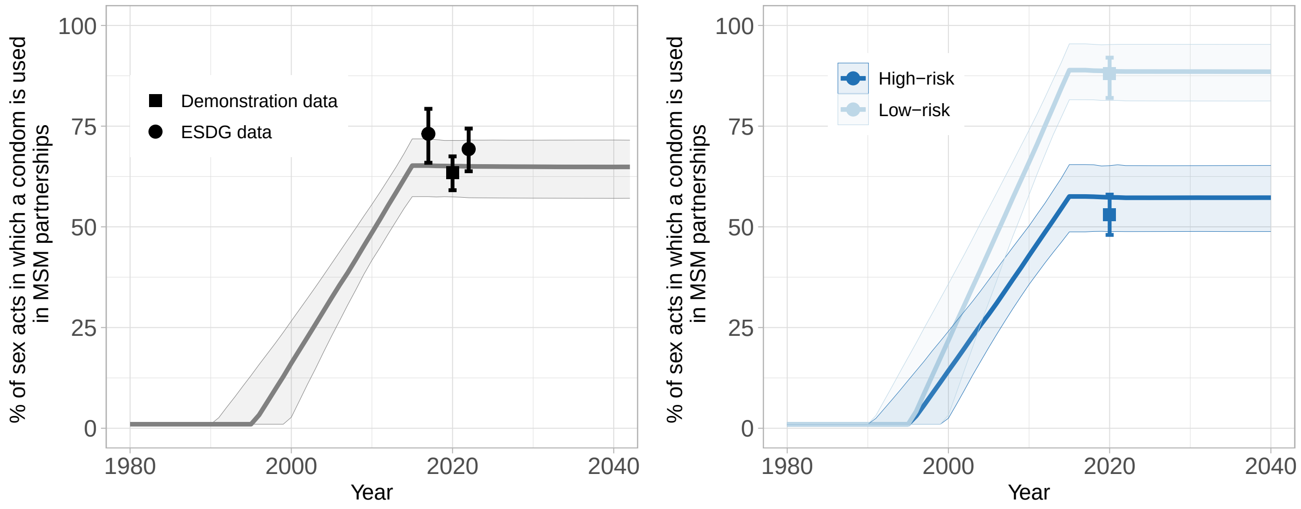

Figure S13: **Trends in condom use in the fitted model.** Trends in the modelled percentage of anal sex acts between MSM in which a condom is used, a) all sexually active MSM and b) sexually active MSM by risk (dark blue - high-risk, light blue - low-risk). Markers (circles for ESDG data, squares for demonstration project data) denote point estimates of the percentage of MSM reporting having used a condom at the last anal sex act. Cross-bars show 95% confidence intervals. In both panels, solid lines and shaded ribbons represent median model predictions and 95% uncertainty intervals from 1000 posterior parameter sets.

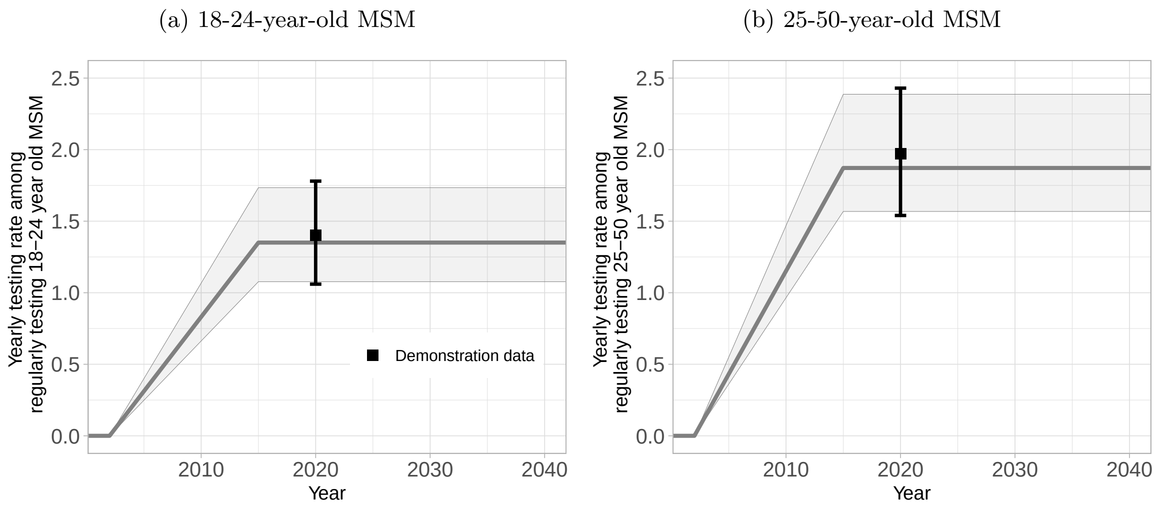

Figure S14: **Trends in HIV testing rates among routinely testing MSM in the fitted model.** Trends in the modelled yearly HIV testing rates for routinely testing MSM for a) 18–24-year-old MSM and b) 25-50-year-old MSM. Square markers denote point estimates from demonstration project data, inferred from the proportion of MSM reporting having tested for HIV in the previous 12 months, and cross-bars show 95% confidence intervals. In both panels, solid lines and shaded ribbons represent median model predictions and 95% uncertainty intervals from 1000 posterior parameter sets.

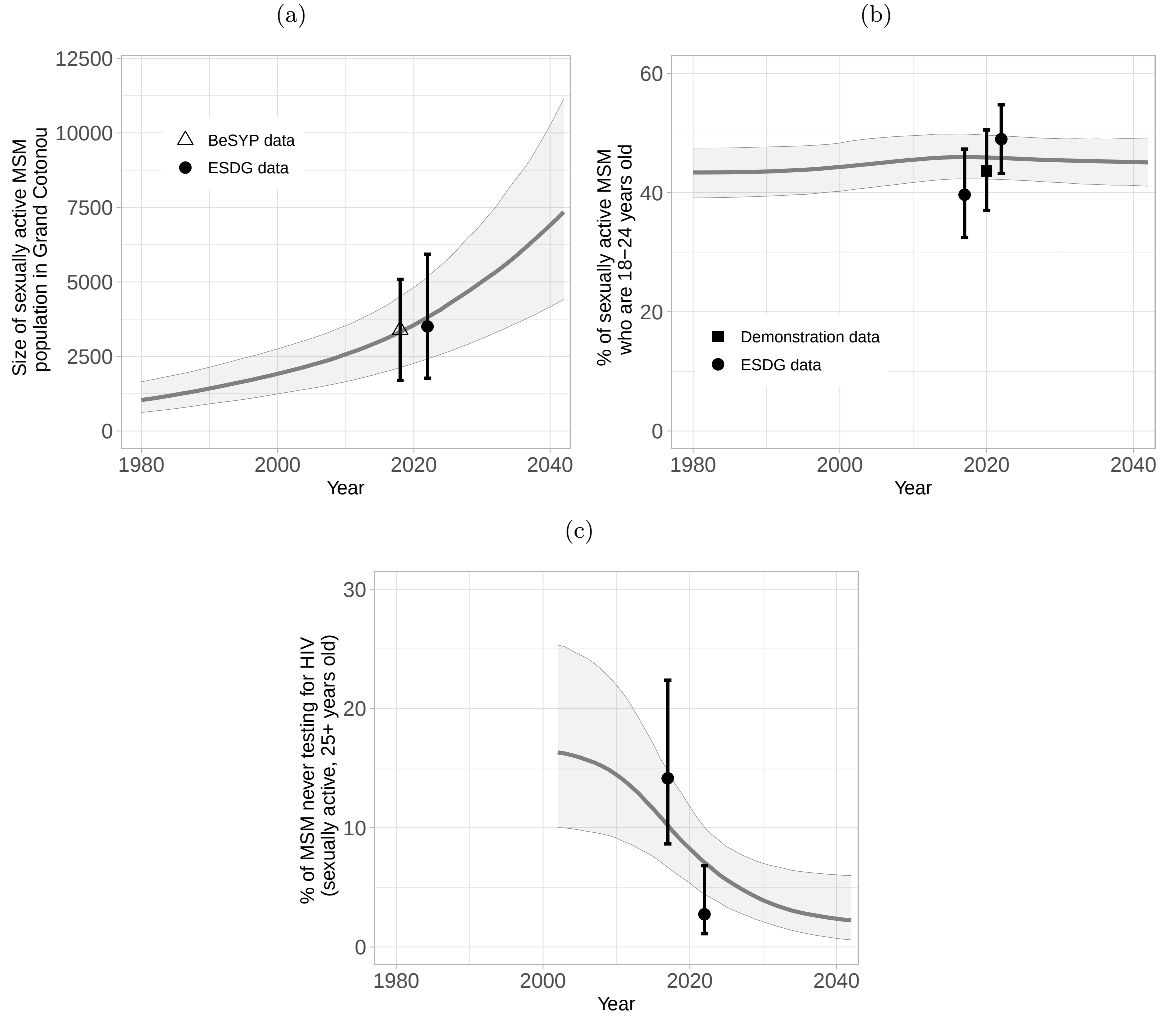

Figure S15: **Model fits to population size, age distribution, and testing.** a) the size of the sexually MSM population in Grand Cotonou, b) the percentage of sexually active MSM who are 18–24-years old, c) the percentage of sexually active 25–50-year-old MSM who have never taken a HIV test, taken as a proxy for the proportion of 25–50-year-old sexually active MSM who never routinely test for HIV. Circle markers represent estimates from data from ESDG surveys, while triangular points represent population size estimates from the BeSYP network of LGBTIQ associations in Benin. Cross-bars show 95% confidence interval estimates from data. In all panels, solid lines and shaded ribbons represent median model predictions and 95% uncertainty intervals from 1000 posterior parameter sets.

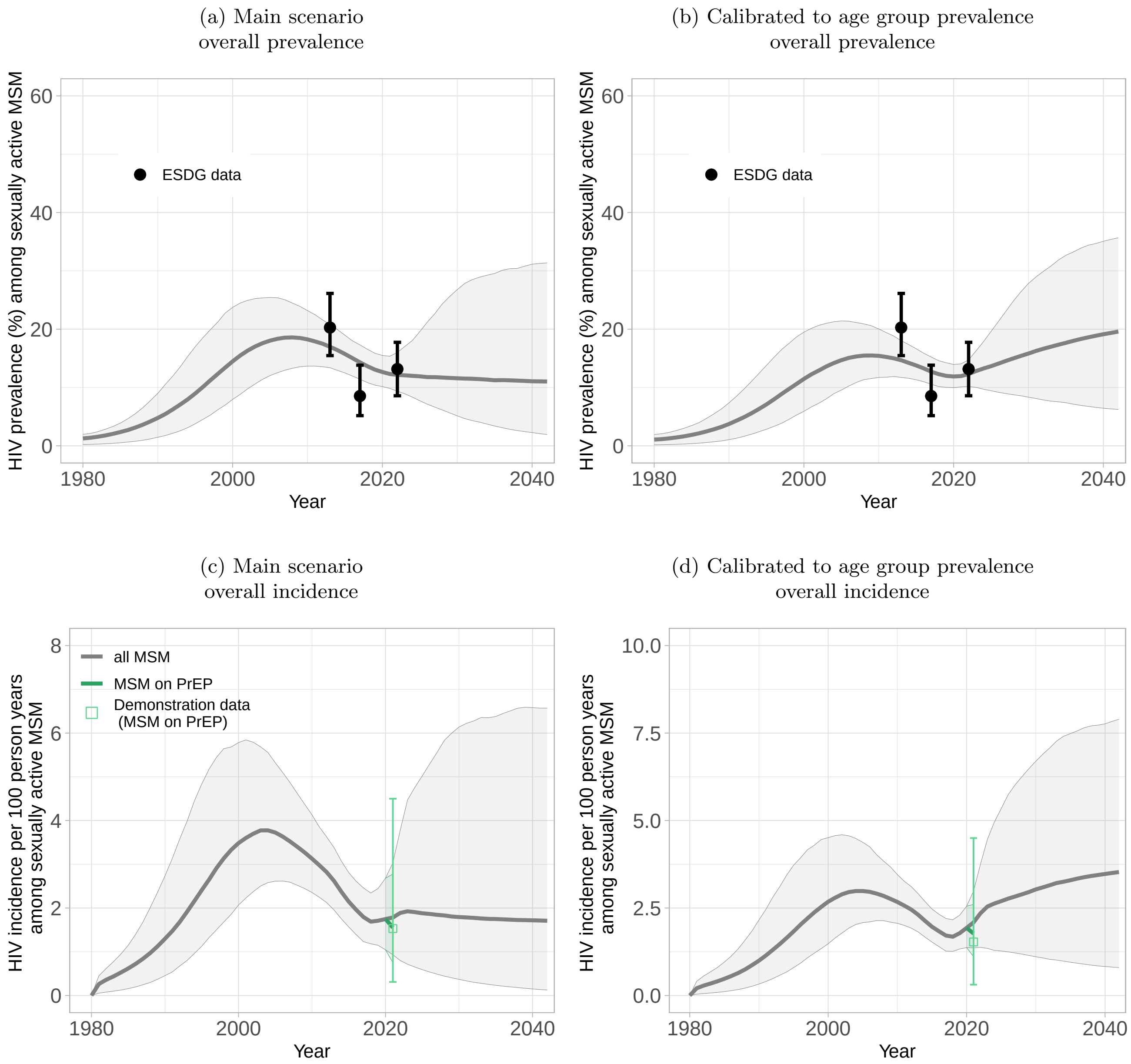

Figure S16: **Comparison of incidence trends between models fitted to overall prevalence and models additionally fitted to age-specific prevalence.** a-b) HIV prevalence trend and c-d) HIV incidence trends from our main model calibration, calibrated to overall prevalence (left) and an alternative calibration calibrated to baseline fitting criteria alongside age-group specific prevalence estimates (right) (criteria shown in Table S11). Square point makers denote point estimates of HIV incidence among study participants in the 2020 PrEP demonstration project. Cross-bars showing 95% confidence intervals. In all panels, solid lines and shaded ribbons represent median model predictions and 95% uncertainty intervals from 1000 posterior parameter sets.

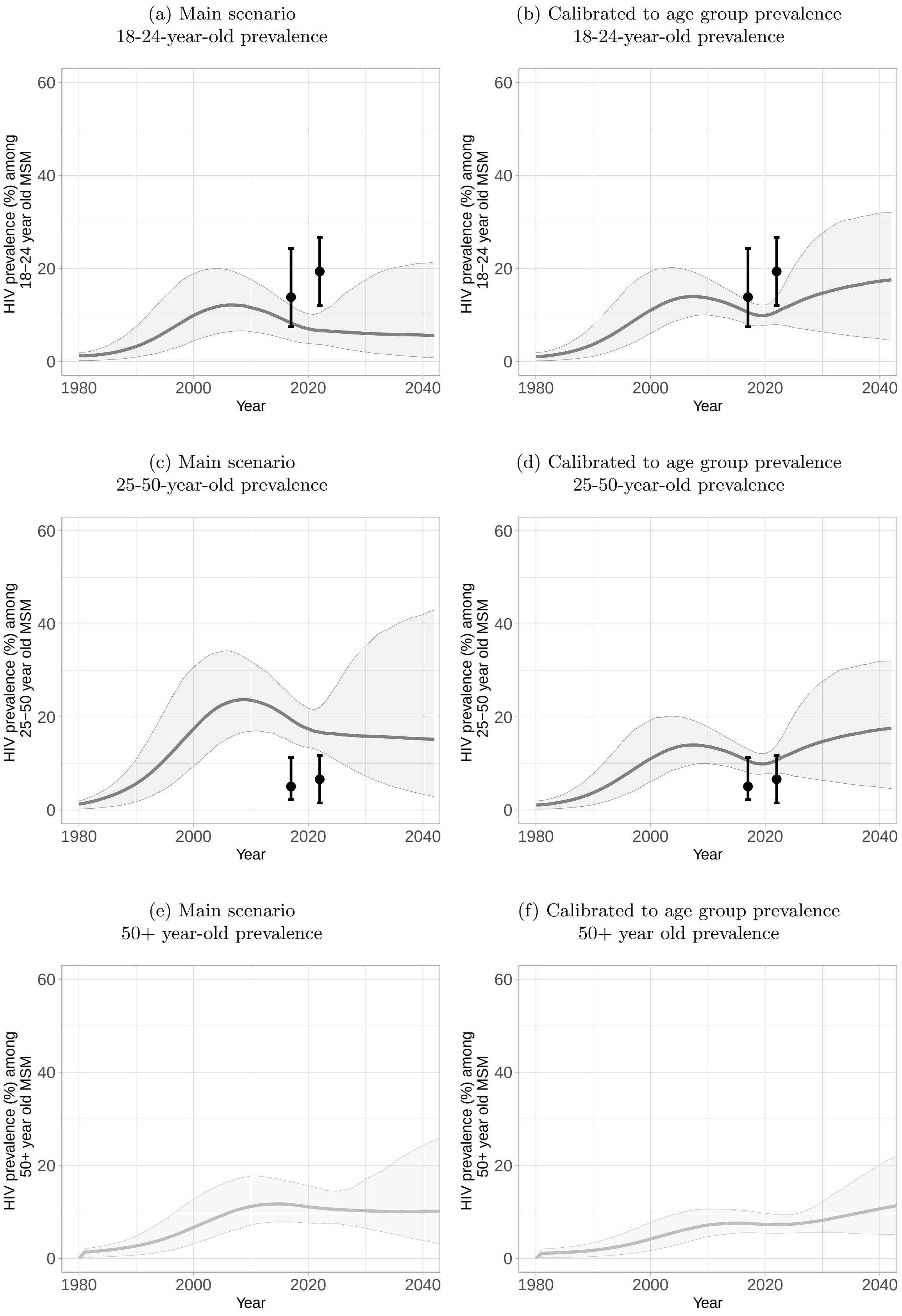

Figure S17: **Comparison between models fitted to overall prevalence and models additionally fitted to age-specific prevalence.** Left column: HIV-prevalence trends from our main model calibration, calibrated to overall prevalence. Right column: HIV-prevalence trends from an alternative calibration calibrated to baseline fitting criteria alongside age-group specific prevalence estimates (shown in Table S11). a–b) HIV prevalence trends among 18-24-year-old MSM through time. c–f) HIV prevalence among 25-50-year-old MSM, e-f) HIV prevalence among 50+ year-old (sexually inactive) MSM. Circles denote point estimates from ESDG MSM surveys, and cross-bars showing 95% confidence intervals. In all panels, solid lines and shaded ribbons represent median model predictions and 95% uncertainty intervals from 1000 posterior parameter sets.

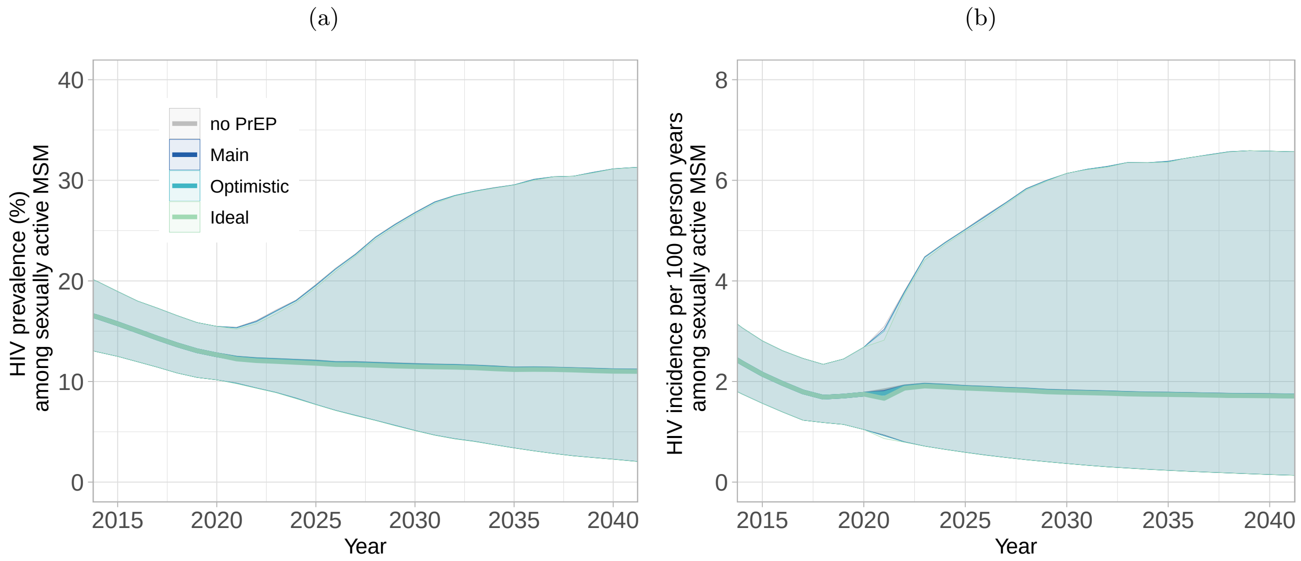

Figure S18: **Prevalence and incidence trends for the 1-year PrEP demonstration project.** a) HIV prevalence trends and b) HIV incidence trends with a 1-year PrEP demonstration project, under main (dark blue), optimistic (blue) and ideal (green) adherence scenarios. Cross-bars showing 95% confidence intervals. In all panels, solid lines and shaded ribbons represent median model predictions and 95% uncertainty intervals from 1000 posterior parameter sets.

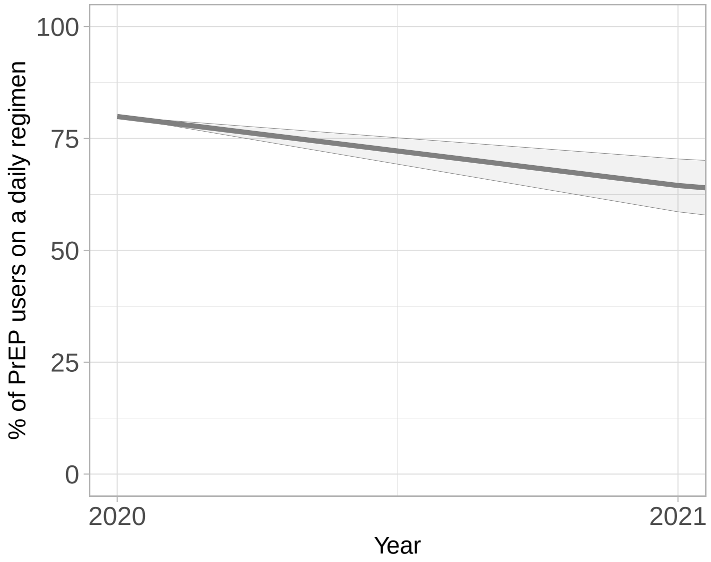

Figure S19: **PrEP regimen choice for the 1-year demonstration project.** Modelled trends in the percentage of PrEP users who are on a daily oral PrEP regimen during the 1-year demonstration. Solid lines and shaded ribbons represent median model predictions and 95% uncertainty intervals from 1000 posterior parameter sets.

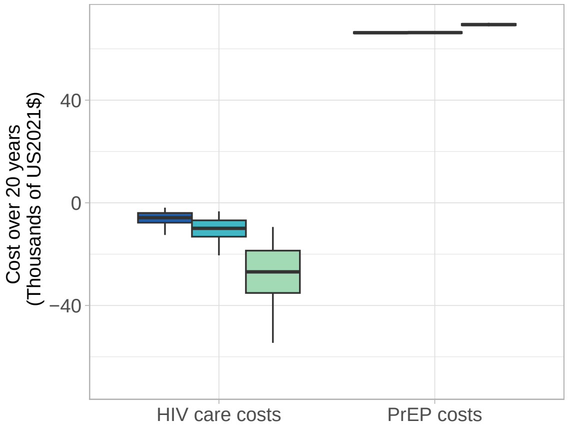

Figure S20: **The breakdown of costs over 20 years for the 1-year PrEP demonstration project in 2020.** HIV care costs and PrEP costs over 20 years from a 1-year demonstration project, compared to a counterfactual scenario where PrEP is not introduced, main (dark blue), optimistic (blue), and ideal (green) adherence scenarios. Whiskers represent the 2.5th and 97.5th percentiles, boxes indicate the 25th and 75th percentiles, and the central line represents the median value. Box plots depict model predictions generated from 1000 posterior parameter sets.

(a)

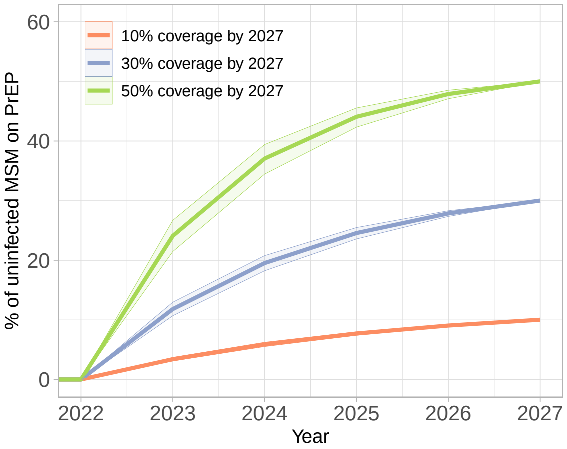

(b)

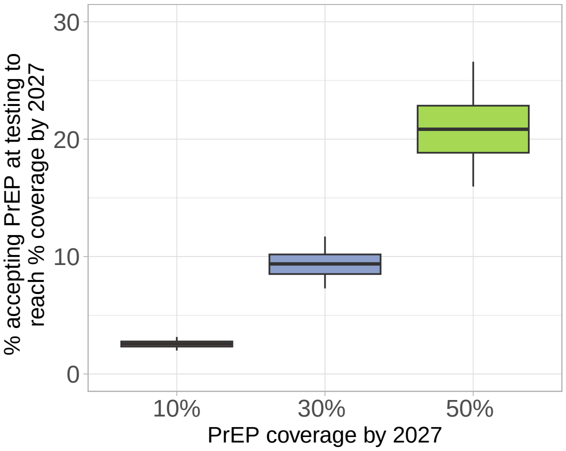

(c)

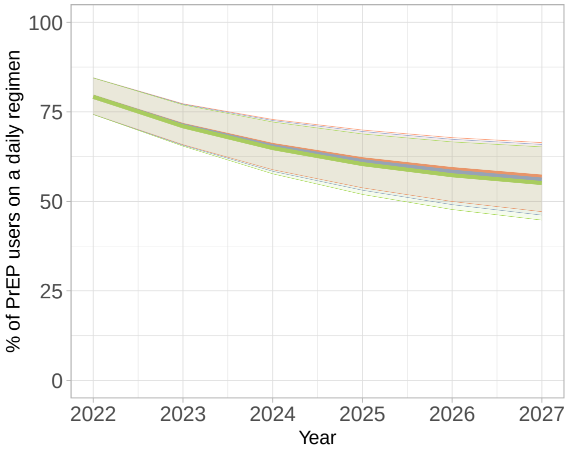

Figure S21: **Acceptance, coverage, and PrEP regimen choice for a 5-year PrEP scale-up from 2022.** a) Trends in the percentage of uninfected MSM on PrEP, for scenarios where 10% (orange), 30% (blue), and 50% (green) of uninfected MSM are on PrEP by 2027, for a PrEP scale-up from 2022 b) Box plots showing the percentage of MSM who accept PrEP at testing to reach 10%, 30%, and 50% of uninfected MSM on PrEP by 2027. Whiskers represent the 2.5th and 97.5th percentiles, boxes indicate the 25th and 75th percentiles, and the central line represents the median value. c) Trends in the percentage of PrEP users who are on a daily oral PrEP regimen during the 5-year PrEP scale-up. In a) and c), solid lines and shaded ribbons represent median model predictions and 95% uncertainty intervals. In all plots, model results are generated from 1000 posterior parameter sets.

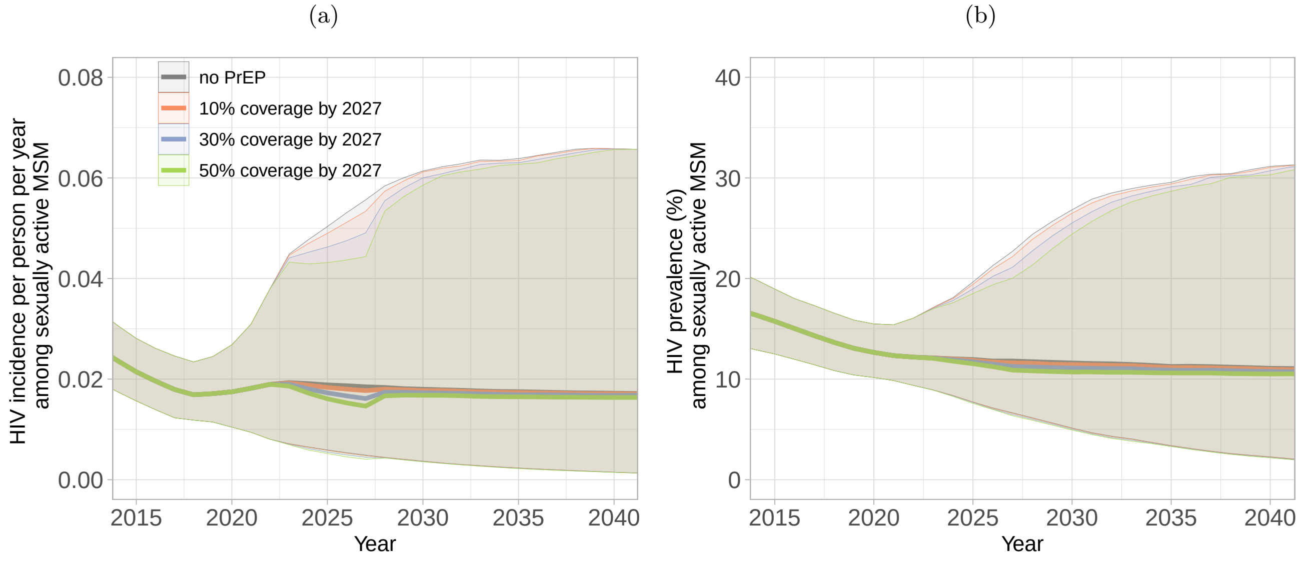

Figure S22: **Trends in HIV prevalence and incidence among sexually active MSM, for a 5-year PrEP scale up from 2022.** Modelled trends in a) HIV prevalence (%), and b) HIV incidence (per person per year) among sexually active MSM, for a PrEP scale-up to MSM in Grand Cotonou from 2022 (orange: 10% coverage, blue: 30% coverage, green: 50% coverage), compared to a scenario where PrEP is not introduced (grey). Solid lines and shaded ribbons represent median model predictions and 95% uncertainty intervals from 1000 posterior parameter sets.

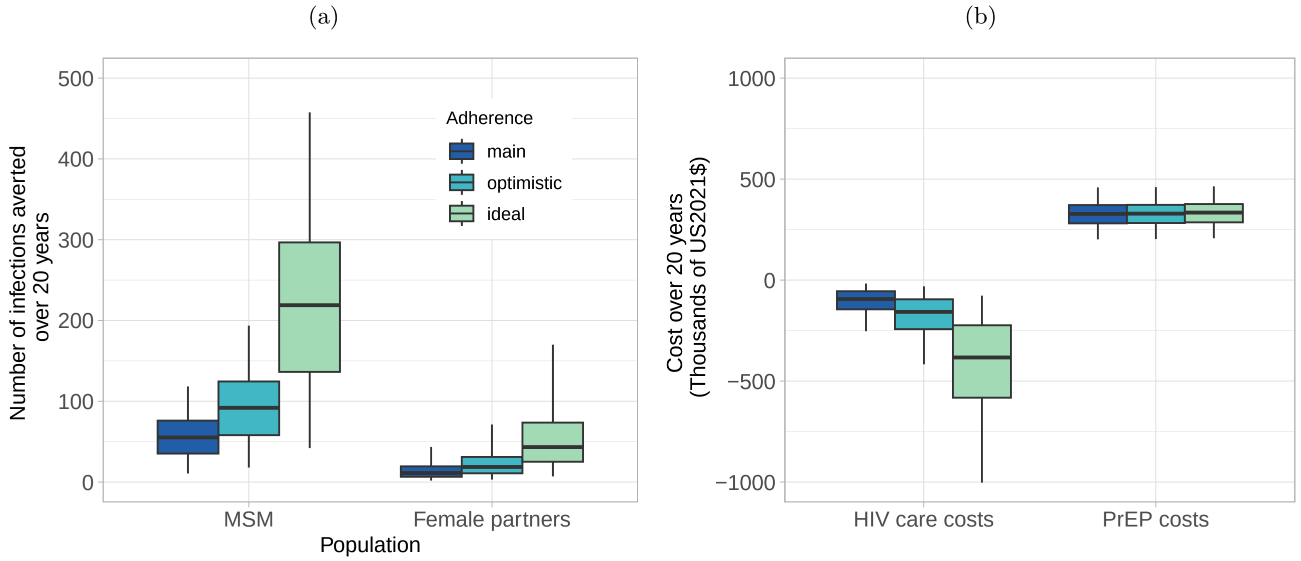

Figure S23: **The breakdown of infections averted and costs over 20 years for a 5-year PrEP scale-up from 2022 with 30% coverage by 2027.** a) The number of HIV infections averted over 20 years among MSM in Grand Cotonou (left) and among female partners of MSM (right), and b) HIV care costs and PrEP costs over 20 years from a 5-year PrEP scale-up, compared to a counterfactual scenario where PrEP is not introduced, for main (dark blue), optimistic (blue), and ideal (green) adherence scenarios. Whiskers represent the 2.5th and 97.5th percentiles, boxes indicate the 25th and 75th percentiles, and the central line represents the median value. Box plots depict model predictions generated from 1000 posterior parameter sets.

(a)

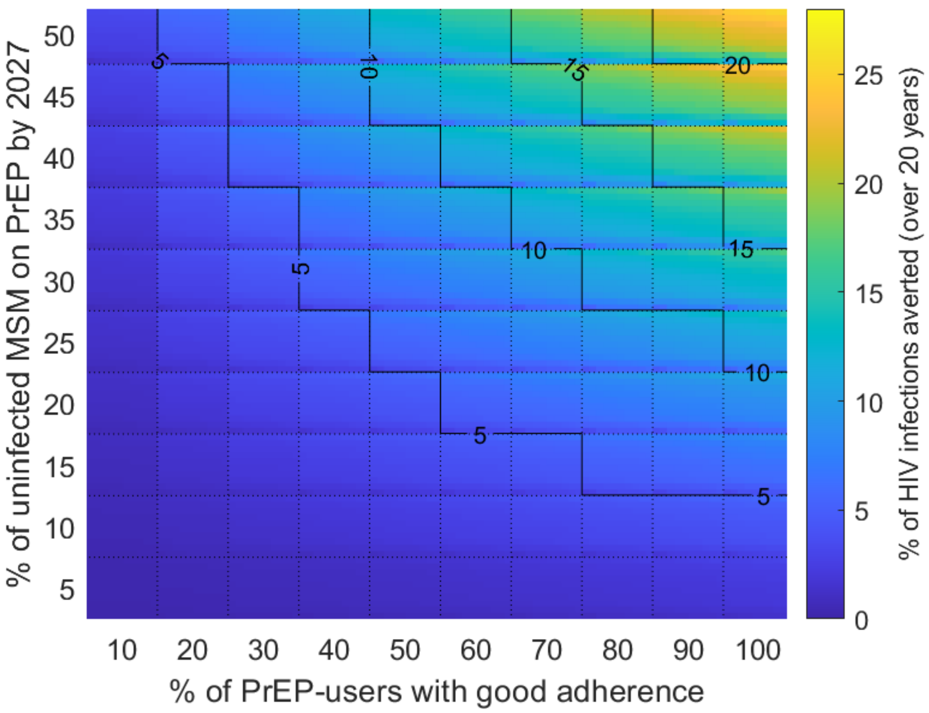

(b)

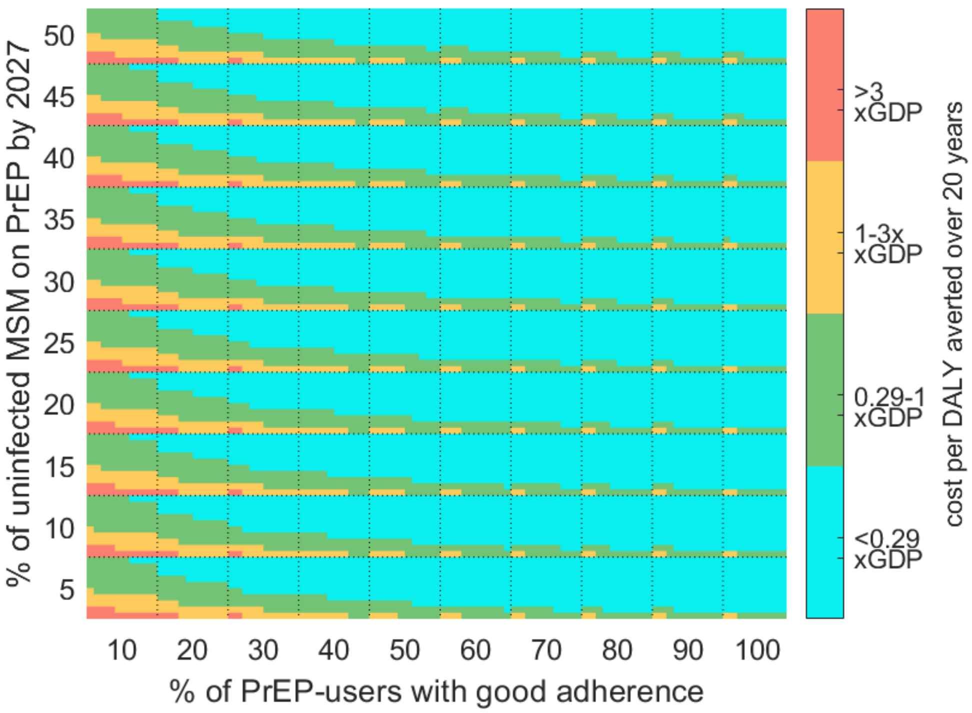

Figure S24: **Relationship between adherence, coverage, impact and cost-effectiveness.** a) Heat map of the percentage of HIV infections averted over 20 years of a 5-year PrEP scale-up to all MSM in Grand Cotonou from 2022, with colours corresponding to impact estimates. To the right of indicated contour lines, the median impact for each coverage and adherence combination is above the value specified on that line. b) Waffle plot showing the proportion of model runs with a cost per DALY averted greater than 3xGDP (red), 1–3xGDP (yellow), 0.29–1xGDP (green), and <0.29xGDP (light blue). In all cases, we assume 0% of PrEP users are partially adherent. Results are generated model runs from 100 posterior parameter sets for each coverage and adherence combination

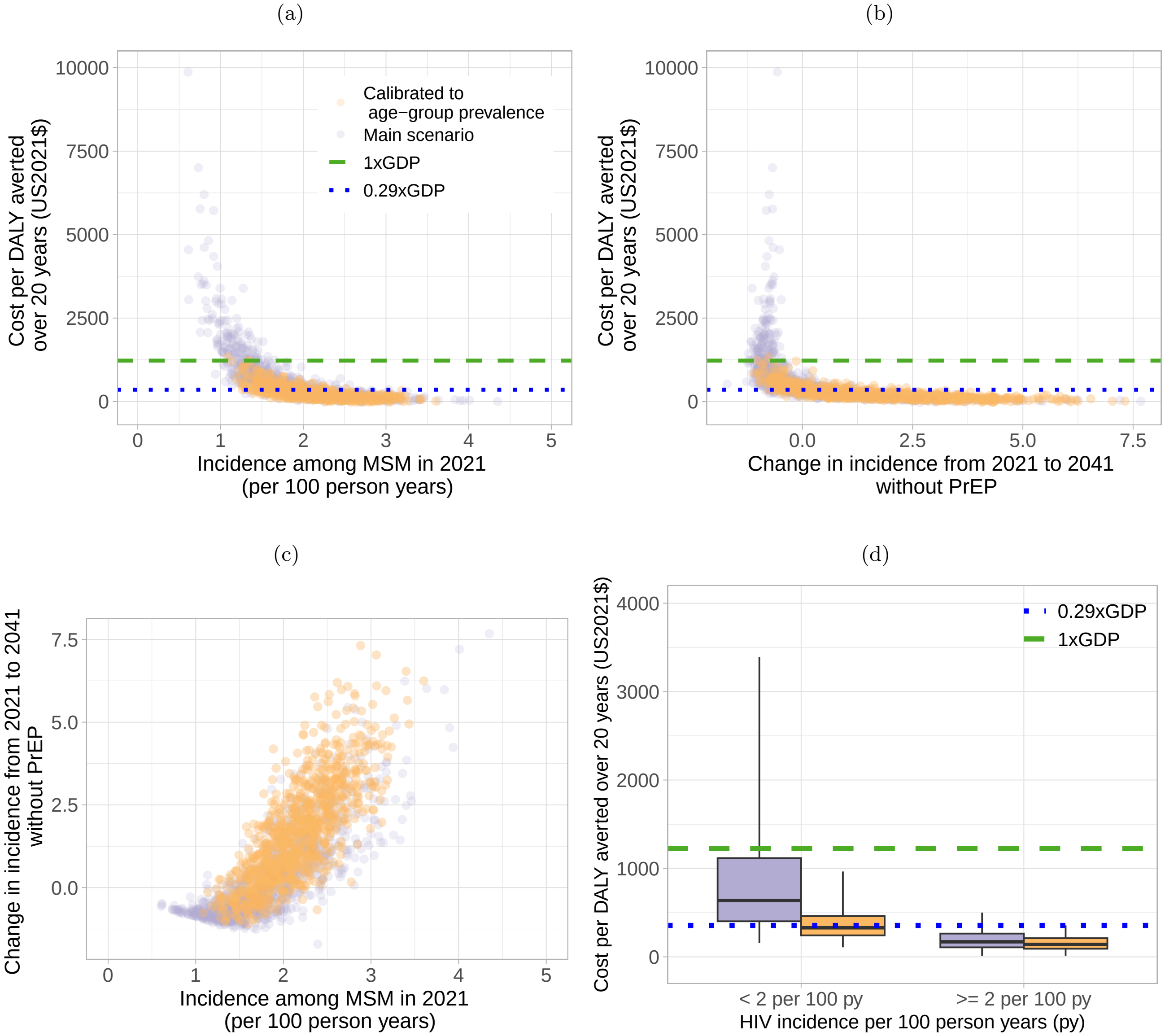

Figure S25: **The relationship between incidence and cost-effectiveness of a 5-year PrEP scale-up from 2022.** Scatter plots of a) HIV incidence (per 100 person years) in 2021 against cost per DALY averted over 20 years for PrEP scale-up with 30% coverage by 2027. b) The absolute change in HIV incidence (per 100 person years) from 2041 to 2021 in a scenario without PrEP against cost per DALY averted over 20 years for PrEP scale-up with 30% coverage by 2027(numbers > 0 represent an increase in incidence over time, and numbers < 0 represent a decrease in incidence over time), and c) HIV incidence in 2021 against the change in incidence from 2041 to 2021 in a scenario without PrEP. Purple circles represent results from our main model calibration, and orange markers represent results from an alternative calibration calibrated to baseline fitting criteria alongside age-group specific prevalence estimates (shown in Table S11). d) Box plots of cost per DALY averted over 20 years under each calibration considering model runs with HIV incidence < 2 per 100 person years in 2021 (left) and ≥ 2 per 100 person years in 2021 (right). Dashed green line and dark dotted line on panel F indicate 1xGDP and 0.29xGDP per capita cost-effectiveness thresholds, respectively. Whiskers represent the 2.5th and 97.5th percentiles, boxes indicate the 25th and 75th percentiles, and the central line represents the median value. For each calibration, results are generated from model predictions from 1000 posterior parameter sets.

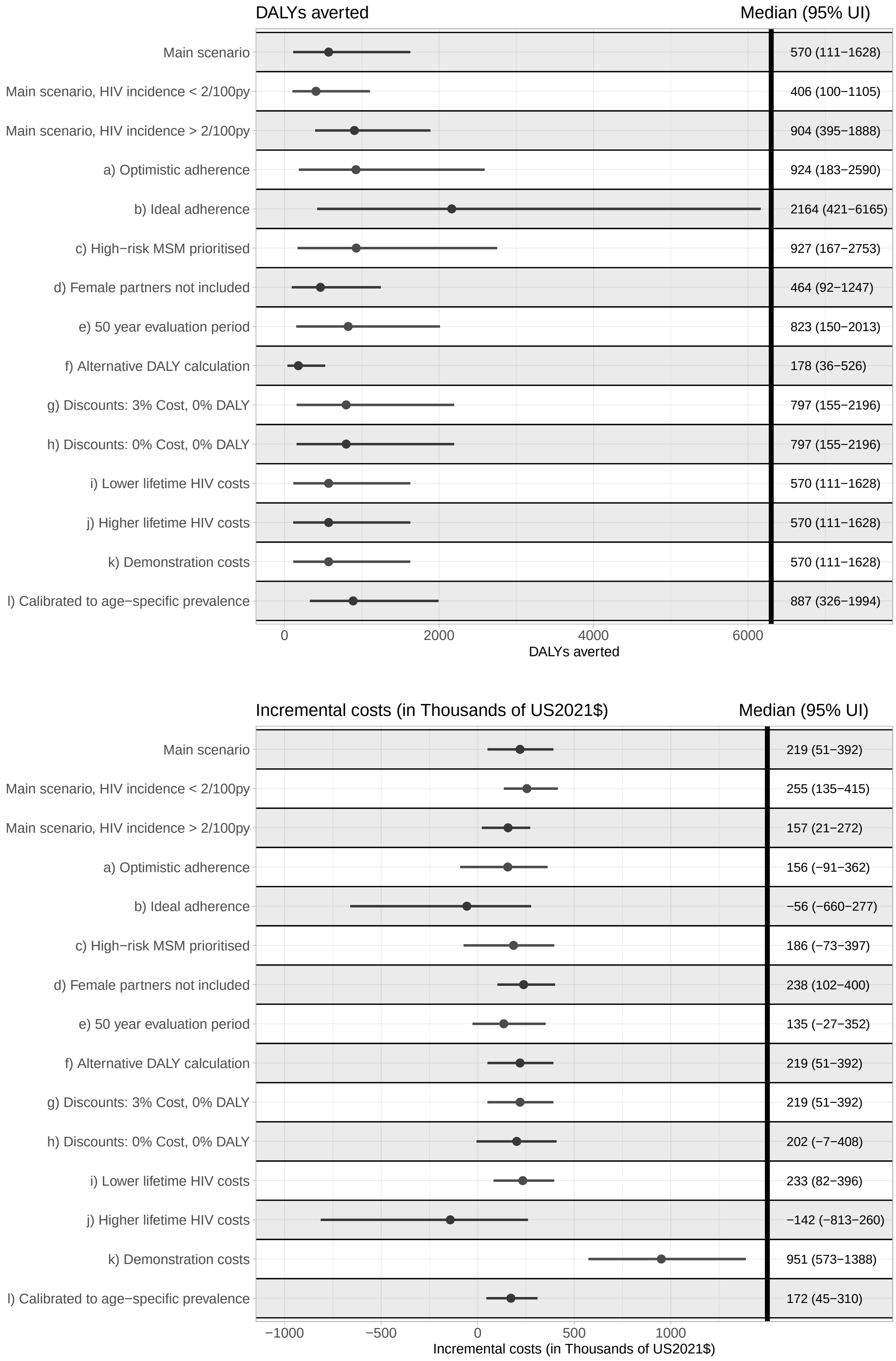

Figure S26: **Sensitivity analysis of DALYs averted and excess costs from a 5-year PrEP scale-up from 2022, with 30% coverage by 2027.** Above: DALYs averted and below: excess costs (costs of intervention minus costs of a counterfactual scenario without PrEP), from a PrEP scale-up to MSM in Grand Cotonou under our main scenario (overall and stratified by HIV incidence in 2021), alongside a range of adherence (a-b), distribution (c), DALY calculation (d-h),costing (i-k), and calibration (l) sensitivity scenarios (described in Table S12). In all scenarios, model predictions are generated from 1000 posterior parameter sets. Points and lines indicate median values and 95% uncertainty intervals, respectively.

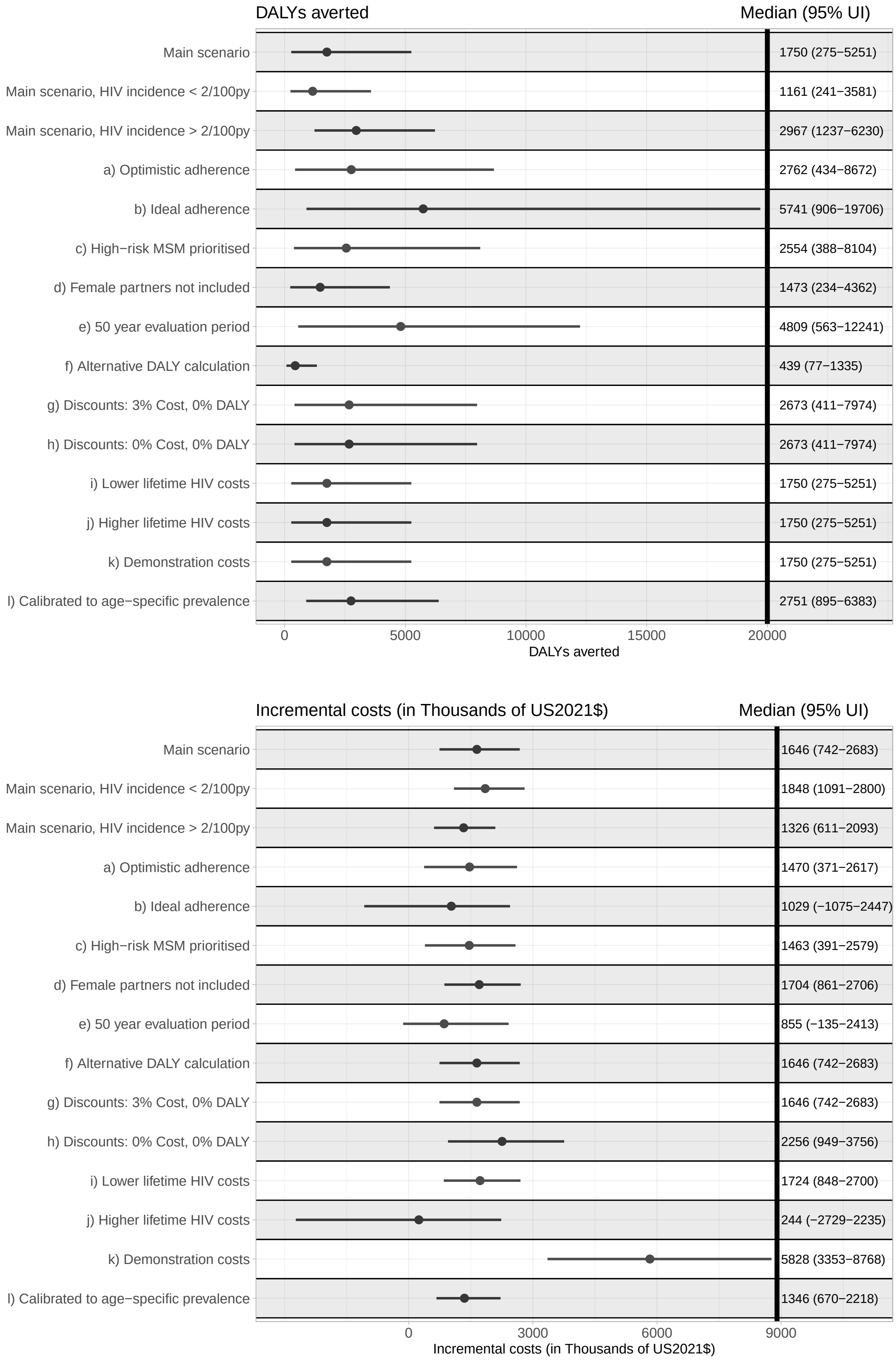

Figure S27: **Sensitivity analysis of DALYs averted and excess costs from a 20-year PrEP scale-up from 2022, with 30% coverage by 2027.** Above: DALYs averted and below: excess costs (costs of intervention minus costs of a counterfactual scenario without PrEP), from a PrEP scale-up to MSM in Grand Cotonou under our main scenario (overall and stratified by HIV incidence in 2021), alongside a range of adherence (a-b), distribution (c), DALY calculation (d-h),costing (i-k), and calibration (l) sensitivity scenarios (described in Table S12). In all scenarios, model predictions are generated from 1000 posterior parameter sets. Points and lines indicate median values and 95% uncertainty intervals, respectively.

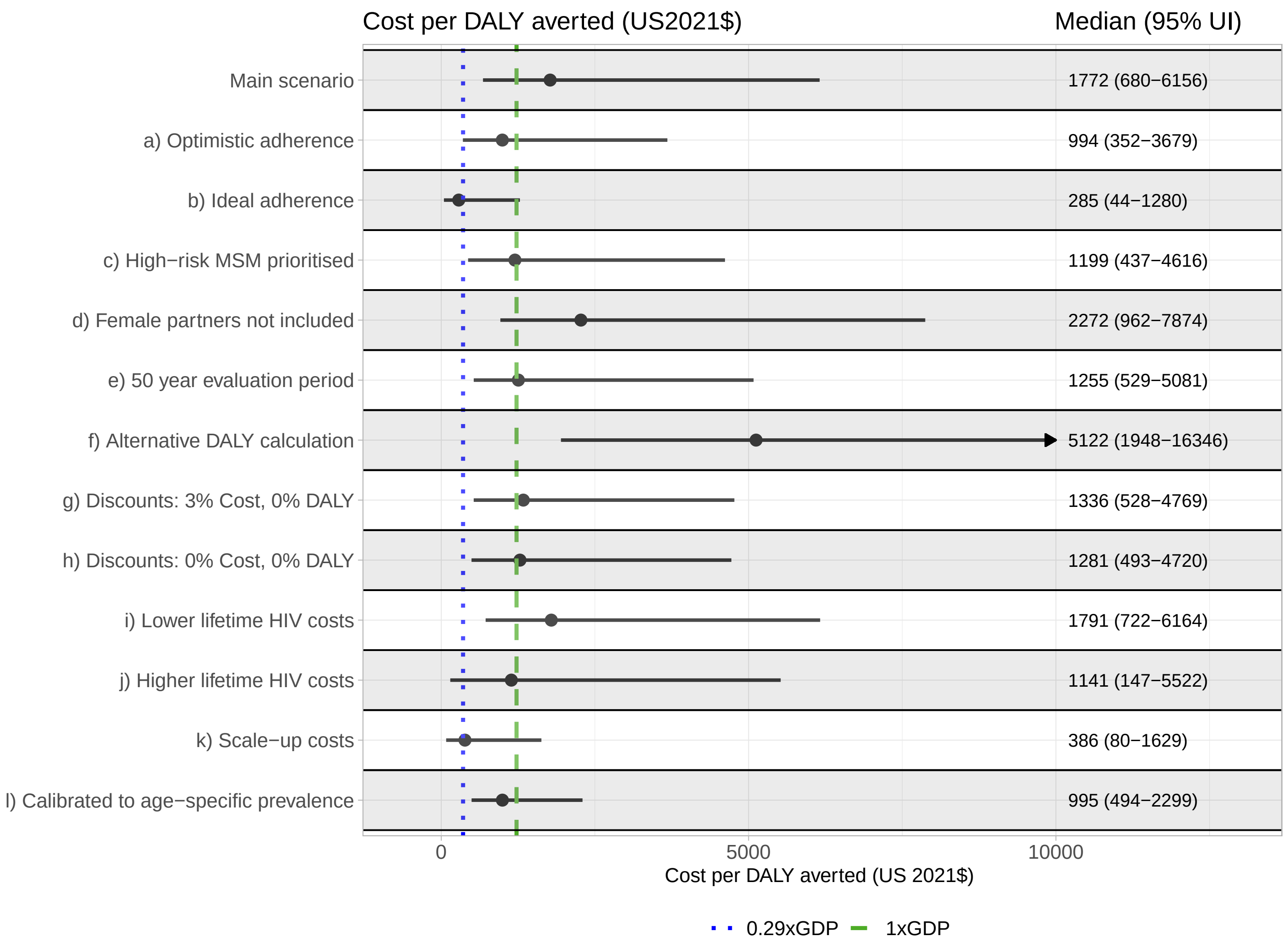

Figure S28: **Sensitivity analysis of the cost-effectiveness of a 1-year PrEP demonstration project in 2020.** Cost per DALY averted from a 1-year Cotonou MSM PrEP demonstration project in 2020 under under a range of adherence (a-b), distribution (c), DALY calculation (d-h),costing (i-k), and calibration (l) sensitivity scenarios (described in Table S12). In all scenarios, model predictions are generated from 1000 posterior parameter sets. Points and lines indicate median values and 95% uncertainty intervals, respectively. Note that costs per DALY averted are shown on a log-scale axis. In all scenarios, model predictions are generated from 1000 posterior parameter sets. Points and lines indicate median values and 95% uncertainty intervals, respectively.

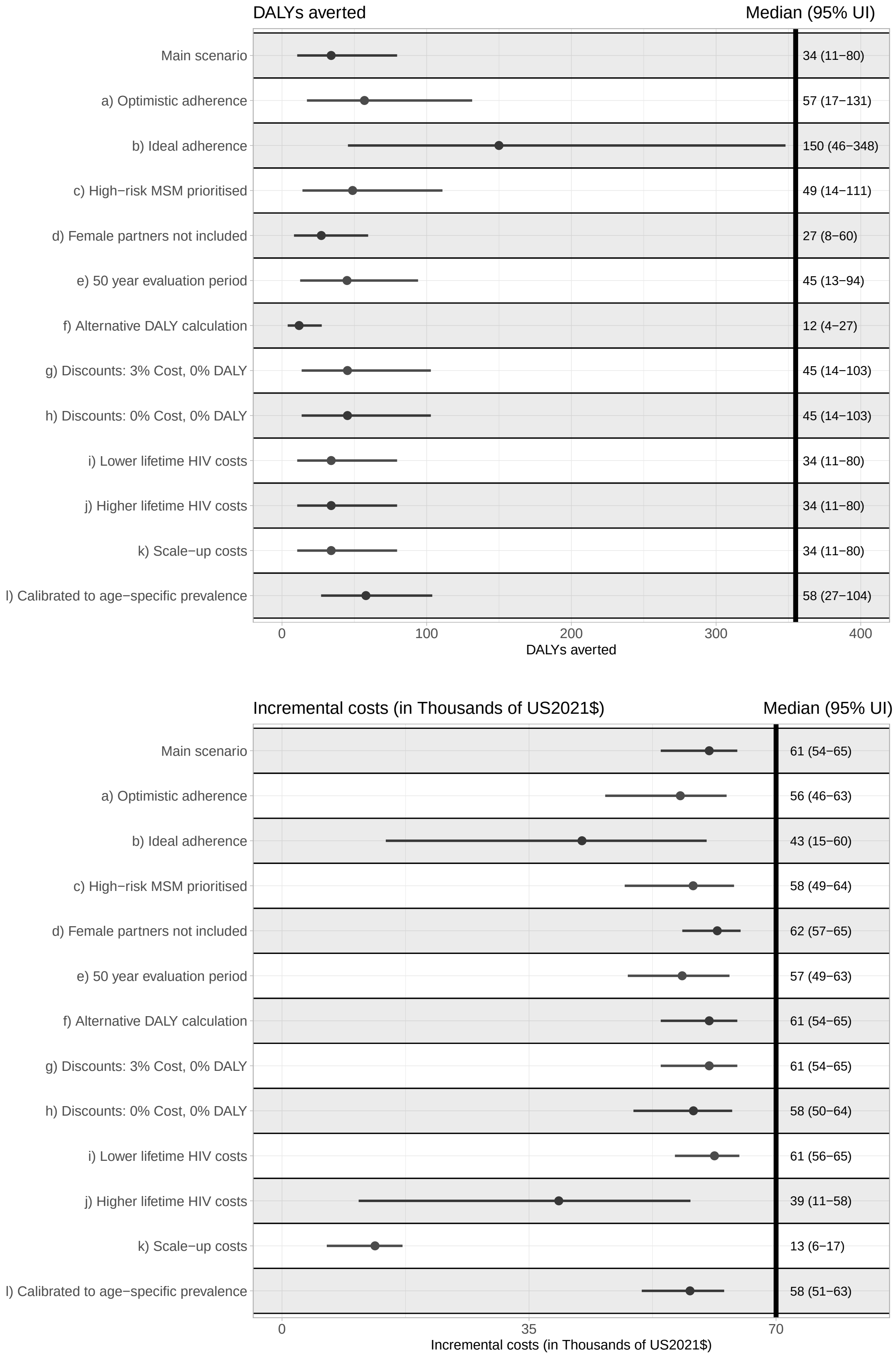

Figure S29: **Sensitivity analysis of DALYs averted and excess costs from a 1-year Cotonou MSM PrEP demonstration project in 2020.** Above: DALYs averted and below: excess costs (costs of intervention minus costs of a counterfactual scenario without PrEP), from a 1-year demonstration project under a range of adherence (a-b), distribution (c), DALY calculation (d-h),costing (i-k), and calibration (l) sensitivity scenarios (described in Table S12). In all scenarios, model predictions are generated from 1000 posterior parameter sets. Points and lines indicate median values and 95% uncertainty intervals, respectively.

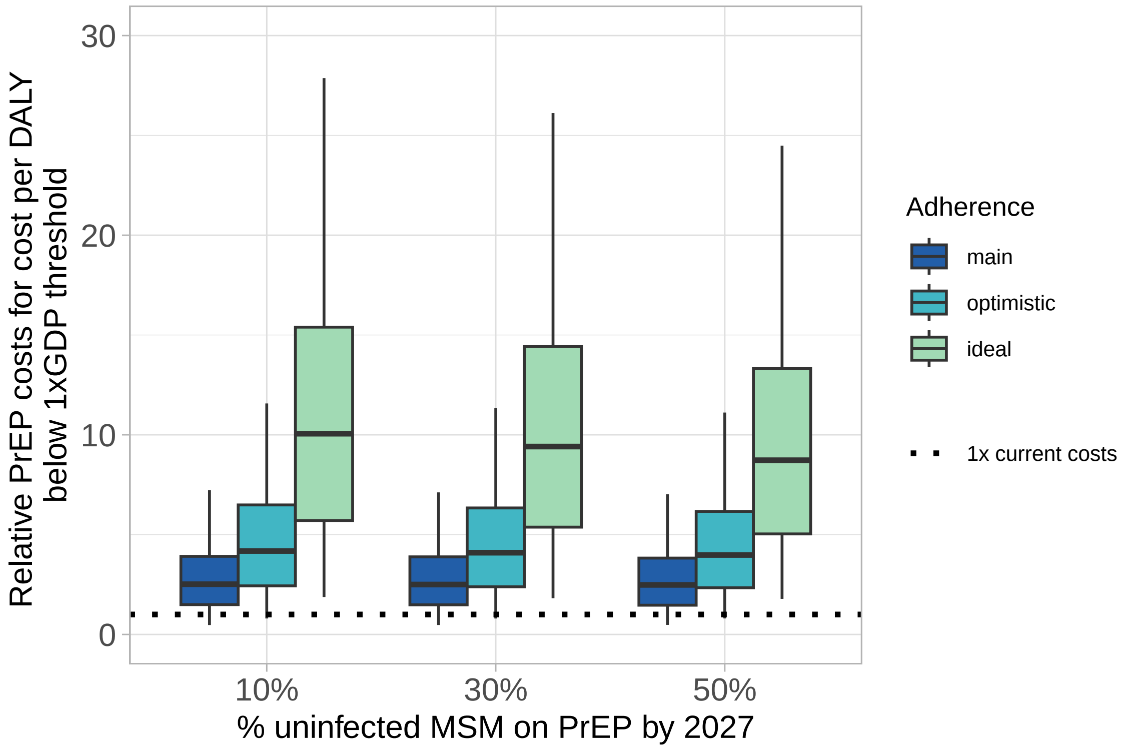

Figure S30: **Threshold analysis for the cost-effectiveness of a 5-year PrEP scale-up from 2022.** Box plots of relative PrEP costs (initiation and ongoing costs) for which a 5-year PrEP scale-up at 30% coverage by 2027 to MSM would be cost-effective at a 1×GDP threshold for main (dark blue), optimistic (blue), and ideal (green) adherence scenarios. If relative PrEP costs are below 1 for cost-effectiveness, then PrEP costs would have to be reduced for cost-effectiveness. If relative PrEP costs are above 1, then PrEP costs could be higher and PrEP scale-up could remain a cost-effective intervention (at a 1×GDP threshold). Whiskers represent the 2.5th and 97.5th percentiles, boxes indicate the 25th and 75th percentiles, and the central line represents the median value. Box plots depict model predictions generated from 1000 posterior parameter sets.

4 CHEERS checklist

CHEERS 2022 Checklist

	Item	Guidance for Reporting	Reported in section
TITLE			
Title	1	Identify the study as an economic evaluation and specify the interventions being compared.	Title
ABSTRACT			
Abstract	2	Provide a structured summary that highlights context, key methods, results and alternative analyses.	Summary
INTRODUCTION			
Background and objectives	3	Give the context for the study, the study question and its practical relevance for decision making in policy or practice.	Introduction
METHODS			
Health economic analysis plan	4	Indicate whether a health economic analysis plan was developed and where available.	Can be accessed as part of the study protocol: available on request, this is indicated in 'Data sharing'
Study population	5	Describe characteristics of the study population (such as age range, demographics, socioeconomic, or clinical characteristics).	Methods (Model structure)
Setting and location	6	Provide relevant contextual information that may influence findings.	Discussion
Comparators	7	Describe the interventions or strategies being compared and why chosen.	Methods (PreP scenarios)
Perspective	8	State the perspective(s) adopted by the study and why chosen.	Methods (Cost assumptions)
Time horizon	9	State the time horizon for the study and why appropriate.	Methods (Model outcomes, Cost assumptions)
Discount rate	10	Report the discount rate(s) and reason chosen.	Methods (Model outcomes, Cost assumptions)
Selection of outcomes	11	Describe what outcomes were used as the measure(s) of benefit(s) and harm(s).	Methods (Model outcomes)
Measurement of outcomes	12	Describe how outcomes used to capture benefit(s) and harm(s) were measured.	Main: Methods (Model outcomes)<br><br>Supplement: Table S11
Valuation of outcomes	13	Describe the population and methods used to measure and value outcomes.	Methods (Model outcomes)
Measurement and valuation of resources and costs	14	Describe how costs were valued.	Methods (Cost assumptions)
Currency, price date, and conversion	15	Report the dates of the estimated resource quantities and unit costs, plus the currency and year of conversion.	Methods (Cost assumptions)
Rationale and description of model	16	If modelling is used, describe in detail and why used. Report if the model is publicly available and where it can be accessed.	Summary in main paper: Methods (Model structure)<br><br>Access: Data sharing<br><br>Detailed description in supplement: Section 1
Analytics and assumptions	17	Describe any methods for analysing or statistically transforming data, any extrapolation methods, and approaches for validating any model used.	Validation in main paper: Methods (Model calibration)<br><br>Data analysis details in supplement: Section 1.4.1
Characterizing heterogeneity	18	Describe any methods used for estimating how the results of the study vary for sub-groups.	Female partners in main paper: Methods, and in supplement, Section 1.5.5, 1.5.7 and 1.6.4
Characterizing distributional effects	19	Describe how impacts are distributed across different individuals or adjustments made to reflect priority populations.	N/A – focussed on KP
Characterizing uncertainty	20	Describe methods to characterize any sources of uncertainty in the analysis.	Main: Methods (Sensitivity analysis)<br>Supplement: (Table S12)
Approach to engagement with patients and others affected by the study	21	Describe any approaches to engage patients or service recipients, the general public, communities, or stakeholders (e.g., clinicians or payers) in the design of the study.	Data derived from community-based participatory research study, for details see Diabaté et al. 2023 https://doi.org/10.1002/jia2.26130
RESULTS			
Study parameters	22	Report all analytic inputs (e.g., values, ranges, references) including uncertainty or distributional assumptions.	Main: Table 1<br>Supplement Table S1-S8
Summary of main results	23	Report the mean values for the main categories of costs and outcomes of interest and summarise them in the most appropriate overall measure.	Overall measure in main paper: Results<br>Supplement:<br>Figure S20 and S23
Effect of uncertainty	24	Describe how uncertainty about analytic judgments, inputs, or projections affect findings. Report the effect of choice of discount rate and time horizon, if applicable.	Uncertainty in inputs/ projections in main paper: Results and Figure 4<br>and in supplement: Figure S25<br><br>Impact of time horizon in main paper: Results and Figure 4
Effect of engagement with patients and others affected by the study	25	Report on any difference patient/service recipient, general public, community, or stakeholder involvement made to the approach or findings of the study	N/A

DISCUSSION			
Study findings, limitations, generalizability, and current knowledge	26	Report key findings, limitations, ethical or equity considerations not captured, and how these could impact patients, policy, or practice.	Discussion
OTHER RELEVANT INFORMATION			
Source of funding	27	Describe how the study was funded and any role of the funder in the identification, design, conduct, and reporting of the analysis	How the study was funded: Acknowledgments<br><br>Role of funder: Methods (Role of the funding source)
Conflicts of interest	28	Report authors conflicts of interest according to journal or International Committee of Medical Journal Editors requirements.	Declaration of interests

Husereau D, Drummond M, Augustovski F, de Bekker-Grob E, Briggs AH, Carswell C, Caulley L, Chaiyakunapruk N, Greenberg D, Loder E, Mauskopf J, Mullins CD, Petrou S, Pwu RF, Staniszewska S; CHEERS 2022 ISPOR Good Research Practices Task Force. Consolidated Health Economic Evaluation Reporting Standards 2022 (CHEERS 2022) Statement: Updated Reporting Guidance for Health Economic Evaluations. BMJ. 2022;376:e067975.
The checklist is Open Access distributed in accordance with the terms of the Creative Commons Attribution (CC BY 4.0) license, which permits others to distribute, remix, adapt and build upon this work, for commercial use, provided the original work is properly cited. See: http://creativecommons.org/licenses/by/4.0/.

References

[1] Mitchell KM, Boily MC, Hanscom B, Moore M, Todd J, Paz-Bailey G, et al. Estimating the impact of HIV PrEP regimens containing long-acting injectable cabotegravir or daily oral tenofovir disoproxil fumarate/emtricitabine among men who have sex with men in the United States: A mathematical modelling study for HPTN 083. The Lancet Regional Health-Americas. 2023:100416.

[2] Diabaté S, Béhanzin L, Guédou FA, Goma-Matsétsé E, Olodo M, Aza-Gnandji M, et al. Pre-exposure prophylaxis in real life: experience from a prospective, observational and demonstration project among men who have sex with men in Benin, West Africa. Journal of the International AIDS Society. 2023;26(6):e26130.

[3] Ministry of Health Benin. Enquete de Surveillance de Deuxieme Generation (ESDG) du VIH et des UST Aupres des Hommes Ayant des Rapports Sexuels avec les Hommes (HSH) au Benin [Second Generation Surveillance Survey (ESDG) of HIV and STIs Among Men Who Have Sex with Men (MSM) in Benin]. 2013.

[4] Ministry of Health Benin. Enquete de Surveillance de Deuxieme Generation (ESDG) du VIH et des UST Aupres des Hommes Ayant des Rapports Sexuels avec les Hommes (HSH) au Benin [Second Generation Surveillance Survey (ESDG) of HIV and STIs Among Men Who Have Sex with Men (MSM) in Benin]. 2017.

[5] Ministry of Health Benin. Enquete de Surveillance de Deuxieme Generation (ESDG) du VIH et des UST Aupres des Hommes Ayant des Rapports Sexuels avec les Hommes (HSH) au Benin [Second Generation Surveillance Survey (ESDG) of HIV and STIs Among Men Who Have Sex with Men (MSM) in Benin]. 2022.

[6] Chow EP, Wilson DP, Zhang L. Estimating HIV incidence among female partners of bisexual men in China. International Journal of Infectious Diseases. 2012;16(5):e312-20.

[7] Lewden C, Gabillard D, Minga A, Ekouévi DK, Avit D, Konate I, et al. CD4-specific mortality rates among HIV-infected adults with high CD4 counts and no antiretroviral treatment in West Africa. Journal of Acquired Immune Deficiency Syndromes. 2012;59(2):213.

[8] Mangal TD. Joint estimation of CD4+ cell progression and survival in untreated individuals with HIV-1 infection. AIDS. 2017;31(8):1073.

[9] Badri M, Lawn SD, Wood R. Short-term risk of AIDS or death in people infected with HIV-1 before antiretroviral therapy in South Africa: a longitudinal study. The Lancet. 2006;368(9543):1254-9.

[10] Hollingsworth TD, Anderson RM, Fraser C. HIV-1 transmission, by stage of infection. The Journal of Infectious Diseases. 2008;198(5):687-93.

[11] Payne CF, Houle B, Chinogurei C, Herl CR, Kabudula CW, Kobayashi LC, et al. Differences in healthy longevity by HIV status and viral load among older South African adults: an observational cohort modelling study. The Lancet HIV. 2022;9(10):e709-16.

[12] Edet A, Akinsola H, Bessong PO. Virologic and immunologic responses of patients on highly active antiretroviral therapy in a rural community health centre in Limpopo, South Africa: A retrospective study. Southern African Journal of HIV Medicine. 2019;20(1):1-7.

[13] Jiamsakul A, Kariminia A, Althoff KN, Cesar C, Cortes CP, Davies MA, et al. HIV viral load suppression in adults and children receiving antiretroviral therapy–results from the IeDEA collaboration. Journal of Acquired Immune Deficiency Syndrome. 2017;76(3):319.

[14] Fatti G, Grimwood A, Nachega J, Nelson J, LaSorda K, Zyl G, et al. Better Virological Outcomes Amongst People Living with HIV Initiating Early Antiretroviral Treatment (CD4 Counts $\geq$ 500 cells/µL) in the HPTN 071 (PopART) Trial in South Africa. Clinical Infectious Diseases. 2020;70:395-403.

[15] Todd J, Glynn JR, Marston M, Lutalo T, Biraro S, Mwita W, et al. Time from HIV seroconversion to death: a collaborative analysis of eight studies in six low and middle-income countries before highly active antiretroviral therapy. AIDS. 2007;21(Suppl 6):S55.

[16] Smith DK, Herbst JH, Zhang X, Rose CE. Condom effectiveness for HIV prevention by consistency of use among men who have sex with men in the United States. Journal of Acquired Immune Deficiency Syndromes. 2015;68(3):337-44.

[17] Rodger AJ, Cambiano V, Bruun T, Vernazza P, Collins S, Van Lunzen J, et al. Sexual activity without condoms and risk of HIV transmission in serodifferent couples when the HIV-positive partner is using suppressive antiretroviral therapy. Journal of the American Medical Association. 2016;316(2):171-81.

[18] Press WH, Teukolsky SA, Vetterling WT, Flannery BP. Numerical recipes 3rd edition: The art of scientific computing. Cambridge university press; 2007.

[19] De Cock KM, Jaffe HW, Curran JW. The evolving epidemiology of HIV/AIDS. Aids. 2012;26(10):1205-13.

[20] Republique du Bénin, Comité National de Lutte contre le Sida. Rapport national de suivi de la déclaration politique sur le VIH/Sida; 2012. Accessed: 7th October 2024. Available from: https://www.unaids.org/sites/default/files/country/documents/ce$_B J_N arrative_R eport[1].pdf$.

[21] United Nations,Department of Economic and Social Affairs, Population Division. World Population Prospects 2022, Online Edition File POP/02-2: Male population by five-year age group, region, subregion and country; 2022.

[22] Papworth E, Grosso A, Ketende S, Wirtz A, Cange C, Kennedy C, et al. Examining risk factors for HIV and access to services among female sex workers (FSW) and men who have sex with men (MSM) in Burkina Faso, Togo and Cameroon. Baltimore: USAID: Project Search: Research to Prevention. 2014.

[23] United Nations,Department of Economic and Social Affairs, Population Division. World Population Prospects 2022, Online Edition File MORT/07-2: Abridged life table, for males, by region, subregion and country, annually for 1950-2100; 2022.

[24] Direction Nationale du Deuxième Recensement Général de la Population et de l'Habitat Y, Cameroun, Enquête Démographique et de Santé Cameroun 1991 [National Directorate of the Second General Census of Population and Housing, Cameroon, Cameroon Demographic and Health Survey] 1991]. 1992.

[25] Geidelberg L, Mitchell KM, Alary M, Mboup A, Béhanzin L, Guédou F, et al. Mathematical Model Impact Analysis of a Real-Life Pre-exposure Prophylaxis and Treatment-As-Prevention Study Among Female Sex Workers in Cotonou, Benin. Journal of Acquired Immune Deficiency Syndrome . 2021;86(2):e28.

[26] Ministry of Health Benin. Enquête de surveillance de Deuxieme Generation des IST/VIH/SIDA au Benin (ESDG-2012): Travailleuses du sexe et Serveuses de bars/restaurants. [Second Generation STI/HIV/AIDS Surveillance Survey in Benin: Sex workers and bar/restaurant waitresses]. 2012.

[27] Ministry of Health Benin. Enquête de surveillance de Deuxieme Generation des IST/VIH/SIDA au Benin (ESDG-2015): Travailleuses du sexe et Serveuses de bars/restaurants [Second Generation STI/HIV/AIDS Surveillance Survey in Benin (ESDG-2012): Sex workers and bar/restaurant waitresses]. 2015.

[28] Baggaley RF, White RG, Boily MC. HIV transmission risk through anal intercourse: systematic review, meta-analysis and implications for HIV prevention. International Journal of Epidemiology. 2010;39(4):1048-63.

[29] Mitchell KM, Dimitrov D, Silhol R, Geidelberg L, Moore M, Liu A, et al. The potential effect of COVID-19-related disruptions on HIV incidence and HIV-related mortality among men who have sex with men in the USA: a modelling study. The Lancet HIV. 2021;8(4):e206-15.

[30] Boily MC, Baggaley RF, Wang L, Masse B, White RG, Hayes RJ, et al. Heterosexual risk of HIV-1 infection per sexual act: systematic review and meta-analysis of observational studies. The Lancet Infectious Diseases. 2009;9(2):118-29.

[31] Bellan SE, Dushoff J, Galvani AP, Meyers LA. Reassessment of HIV-1 acute phase infectivity: accounting for heterogeneity and study design with simulated cohorts. PLoS Medicine. 2015;12(3):e1001801.

[32] Donnell D, Baeten JM, Kiarie J, Thomas KK, Stevens W, Cohen CR, et al. Heterosexual HIV-1 transmission after initiation of antiretroviral therapy: a prospective cohort analysis. The Lancet. 2010;375(9731):2092-8.

[33] Giannou FK, Tsiara CG, Nikolopoulos GK, Talias M, Benetou V, Kantzanou M, et al. Condom effectiveness in reducing heterosexual HIV transmission: a systematic review and meta-analysis of studies on HIV serodiscordant couples. Expert Review of Pharmacoeconomics & Outcomes Research. 2016;16(4):489-99.

[34] Anderson PL, Glidden DV, Liu A, Buchbinder S, Lama JR, Guanira JV, et al. Emtricitabine-tenofovir concentrations and pre-exposure prophylaxis efficacy in men who have sex with men. Science Translational Medicine. 2012;4(151):151ra125-5.

[35] Marrazzo JM, Ramjee G, Richardson BA, Gomez K, Mgodi N, Nair G, et al. Tenofovir-based preexposure prophylaxis for HIV infection among African women. New England Journal of Medicine. 2015;372(6):509-18.

[36] Van Damme L, Corneli A, Ahmed K, Agot K, Lombaard J, Kapiga S, et al. Preexposure prophylaxis for HIV infection among African women. New England Journal of Medicine. 2012;367(5):411-22.

[37] Grant RM, Lama JR, Anderson PL, McMahan V, Liu AY, Vargas L, et al. Preexposure chemoprophylaxis for HIV prevention in men who have sex with men. New England Journal of Medicine. 2010;363(27):2587-99.

[38] Ekouevi DK, Balestre E, Ba-Gomis FO, Eholie SP, Maiga M, Amani-Bosse C, et al. Low retention of HIV-infected patients on antiretroviral therapy in 11 clinical centres in West Africa. Tropical Medicine & International Health. 2010;15:34-42.

[39] UNAIDS. Rapport de suivi de la declaration de politique sur le VIH/SIDA au Benin 2016. [Follow-up report on the policy statement on HIV/AIDS in Benin]. 2016.

[40] Cianci F, Geidelberg L, Mitchell KM, Kessou L, Mboup A, Diabate S, et al. Modelling the cost-effectiveness of TasP and PrEP in female sex workers in Cotonou, Benin. medRxiv. 2023:2023-12.

[41] Tran H, Saleem K, Lim M, Chow EP, Fairley CK, Terris-Prestholt F, et al. Global estimates for the lifetime cost of managing HIV. AIDS. 2021;35(8):1273-81.

[42] Wang H, Abbas KM, Abbasifard M, Abbasi-Kangevari M, Abbastabar H, Abd-Allah F, et al. Global age-sex-specific fertility, mortality, healthy life expectancy (HALE), and population estimates in 204 countries and territories, 1950–2019: a comprehensive demographic analysis for the Global Burden of Disease Study 2019. The Lancet. 2020;396(10258):1160-203.

[43] Sassi F. Calculating QALYs, comparing QALY and DALY calculations. Health Policy and Planning. 2006;21(5):402-8.

[44] US Public Health Service: Preexposure Prophylaxis for the Prevention of HIV Infection in the United States– 2021 Update;. Accessed: 18/04/2023. https://www.cdc.gov/hiv/pdf/risk/prep/cdc-hiv-prep-provider-supplement-2021.pdf.

[45] Foss AM, Vickerman PT, Heise L, Watts CH. Shifts in condom use following microbicide introduction: should we be concerned? AIDS. 2003;17(8):1227-37.

[46] Béhanzin L, Diabaté S, Minani I, Lowndes CM, Boily MC, Labbé AC, et al. Assessment of HIV-related risky behaviour: a comparative study of face-to-face interviews and polling booth surveys in the general population of Cotonou, Benin. Sexually Transmitted Infections. 2013;89(7):595-601.

[47] Wada N, Jacobson LP, Cohen M, French A, Phair J, Muñoz A. Cause-specific mortality among HIV-infected individuals, by CD4+ cell count at HAART initiation, compared with HIV-uninfected individuals. AIDS. 2014;28(2):257.

[48] Sucharitakul K, Boily MC, Dimitrov D, Mitchell KM. Influence of model assumptions about HIV disease progression after initiating or stopping treatment on estimates of infections and deaths averted by scaling up antiretroviral therapy. PLoS One. 2018;13(3):e0194220.

[49] Brooks KM, Anderson PL. Pharmacologic-based methods of adherence assessment in HIV prevention. Clinical Pharmacology and Therapeutics. 2018;104(6):1056.

[50] Anderson PL, Liu AY, Castillo-Mancilla JR, Gardner EM, Seifert SM, McHugh C, et al. Intracellular tenofovir-diphosphate and emtricitabine-triphosphate in dried blood spots following directly observed therapy. Antimicrobial Agents and Chemotherapy. 2018;62(1):e01710-7.

[51] Molina JM, Ghosn J, Assoumou L, Delaugerre C, Algarte-Genin M, Pialoux G, et al. Daily and on-demand HIV pre-exposure prophylaxis with emtricitabine and tenofovir disoproxil (ANRS PREVENIR): a prospective observational cohort study. The Lancet HIV. 2022;9(8):e554-62.

[52] Ministry of Health Benin. Enquete de Surveillance de Deuxieme Generation (ESDG) du VIH et des UST Aupres des Hommes Ayant des Rapports Sexuels avec les Hommes (HSH) au Benin [Second Generation Surveillance Survey (ESDG) of HIV and STIs Among Men Who Have Sex with Men (MSM) in Benin]. 2015.

[53] Mitchell KM, Lépine A, Terris-Prestholt F, Torpey K, Khamofu H, Folayan MO, et al. Modelling the impact and cost-effectiveness of combination prevention amongst HIV serodiscordant couples in Nigeria. Aids. 2015;29(15):2035-44.

[54] BeSyP. BeSyP en Marche: rapport illustré du mapping des hommes qui ont des rapports sexuels avec des hommes au Bénin. Cotonou, Benin: Réseau Bénin Synergie Plus (BeSyP); 2017.

[55] Gelman A, Carlin JB, Stern HS, Dunson DB, Vehtari A, Rubin DB. Bayesian data analysis. CRC press; 2013.

[56] Geweke J. Evaluating the Accuracy of Sampling-Based Approaches to the Calculation of Posterior Moments. In: Bernardo JM, Berger JO, Dawid AP, Smith AFM, editors. Bayesian Statistics 4. Oxford University Press; 1992. p. 169-93.

Supplementary References for the main paper

[1s] Smith DK, Herbst JH, Zhang X, Rose CE. Condom effectiveness for HIV prevention by consistency of use among men who have sex with men in the United States. Journal of Acquired Immune Deficiency Syndromes. 2015;68(3):337-44.

[2s] Rodger AJ, Cambiano V, Bruun T, Vernazza P, Collins S, Van Lunzen J, et al. Sexual activity without condoms and risk of HIV transmission in serodifferent couples when the HIV-positive partner is using suppressive antiretroviral therapy. Journal of the American Medical Association. 2016;316(2):171-81.

[3s] BeSyP. BeSyP en Marche: rapport illustré du mapping des hommes qui ont des rapports sexuels avec des hommes au Bénin. Cotonou, Benin: Réseau B enin Synergie Plus (BeSyP); 2017.

[4s] Ministry of Health Benin. Enquete de Surveillance de Deuxieme Generation (ESDG) du VIH et des UST Aupres des Hommes Ayant des Rapports Sexuels avec les Hommes (HSH) au Benin [Second Generation Surveillance Survey (ESDG) of HIV and STIs Among Men Who Have Sex with Men (MSM) in Benin]. 2013.
