# Supplementary material for: Potential impact and cost-effectiveness of oral HIV pre-exposure prophylaxis for men who have sex with men in Cotonou, Benin: a mathematical modelling study
Source: Lancet Glob Health. Author manuscript; Available in PMC 2025 Sep 30. (PMC12483189; doi:10.1016/S2214-109X(25)00098-1)
Supplement: Supplementary Appendix 3 [file NIHMS2109324-supplement-Supplementary_Appendix_3.pdf]

THE LANCET
Global Health

Supplementary appendix 3

This Equitable Partnership Declaration (EPD) was submitted by the authors, and we reproduce it as supplied. It has not been peer reviewed. *The Lancet*'s editorial processes have not been applied to the EPD.

Supplement to: Leng T, Kessou L, Heitner J, et al. Potential impact and cost-effectiveness of oral HIV pre-exposure prophylaxis for men who have sex with men in Cotonou, Benin: a mathematical modelling study. *Lancet Glob Health* 2025; **13:** e1111–21.

Equitable Partnership Declaration questions

Researcher considerations

1. Please detail the involvement that researchers who are based in the region(s) of study had during a) study design; b) clinical study processes, such as processing blood samples, prescribing medication, or patient recruitment; c) data interpretation; and d) manuscript preparation, commenting on all aspects. If they were not involved in any of these aspects, please explain why.

   This question is intended for international partnerships; if all your authors are based in the area of study, this question is not applicable.

   This should include a thorough description of their leadership role(s) in the study. Are local researchers named in the author list or the acknowledgements, or are they not mentioned at all (and, if not, why)? Please also describe the involvement of early career researchers based in the location of the study. Some of this information might be repeated from the Contributors section in the manuscript. Note: we adhere to ICMJE authorship criteria when deciding who should be named on a paper.

**a) Study design:** Fernand A Guedou, Luc Behanzin<br><br>These co-authors were involved in the conception of the study, and were co-authors of the initial study protocol.
**b) Clinical study processes:** N/A (the study is a modelling analysis of impact and cost-effectiveness, for details of the demonstration project see: https://doi.org/10.1002/jia2.26130)
**c) Data interpretation:** Léon Kessou, Marius Olodo<br><br>These co-authors collected the cost data and conducted the cost analysis and cost report. Mr Olodo (early career researcher) also analysed and interpreted biological adherence data from the study.
**d) Manuscript preparation:** Fernand A Guedou, Luc Behanzin, Léon Kessou, Marius Olodo<br><br>These co-authors were involved in reviewing and editing the manuscript.

2. Were the data used in your study collected by authors named on the paper, or have they been extracted from a source such as a national survey? ie, is this a secondary analysis of data that were not collected by the authors of this paper. If the authors of this paper were not involved in data collection, how were data interpreted with sufficient contextual knowledge?

   The Lancet Global Health *believe contextual understanding is crucial for informed data analysis and interpretation.*

The data underpinning this modelling and cost-effectiveness study was collected during the demonstration project by the authors named on this paper (Fernand A Guedou, Luc Behanzin) alongside individuals not involved in this modelling study, but who have been acknowledged for their contributions to data collection in the demonstration project study publication (https://doi.org/10.1002/jia2.26130). We consulted Plan International Bénin (Carin Ahouada) to

incorporate likely differences in PrEP implementation between the demonstration project and scale-up, and incorporated these changes in our analysis of PrEP scale-up.

This modelling and cost-effectiveness study also used data from national biobehavioural surveillance surveys *(L'enquête de surveillance de deuxième génération (ESDG) auprès des hommes qui ont des rapports sexuels avec d'autres hommes (HSH)),* which was not collected by the study authors. We consulted Plan International Bénin (Carin Ahouada) for context on partner numbers reported in these surveys.

3. How was funding used to remunerate and enhance the skills of researchers and institutions based in the area(s) of study? And how was funding used to improve research infrastructure in the area of study?

   Potentially effective investments into long-term skills and opportunities within institutions could include training or mentorship in analytical techniques and manuscript writing, opportunities to lead all or specific aspects of the study, financial remuneration rather than requiring volunteers, and other professional development and educational opportunities.

   Improvements to research infrastructure could be funding of extended trial designs (such as platform trials) and use of master protocols to enable these designs, establishment of long-term contracts for research staff, building research facilities, and local control of funding allocation.

Skills:
The research team from Université Laval and Imperial College London organised several meetings with the local Benin research team to explain the modelling methodology and familiarise them with modelling principles and the collection of appropriate data for modelling.

Research infrastructure:
N/A (the study is modelling analysis of impact and cost-effectiveness, for details of the demonstration project see: https://doi.org/10.1002/jia2.26130)

4. How did you safeguard the researchers who implemented the study?

   Please describe how you guaranteed safe working conditions for study staff, including provision of appropriate personal protective equipment, protection from violence, and prevention of overworking.

N/A (the study is a modelling analysis of impact and cost-effectiveness, for details of the demonstration project see: https://doi.org/10.1002/jia2.26130)

Benefits to the communities and regions of study

5. How does the study address the research and policy priorities of its location?

   How were the local priorities determined and then used to inform the research question? Who decided which priorities to take forward? Which elements of the study address those priorities?

This work was done as part of the collaborative POCAO research program (Population Clés en Afrique de l'Ouest) with collaborators from Benin (including from the *programme de lutte contre le SIDA*, university of Abomey-Calavi), Mali and Burkina Faso. Priorities for the different POCAO studies, adapted to each country, were discussed and decided at the first meeting of all investigators from each country in Canada in November 2015, at the start of the programme.

This study provides the first estimate of the impact and cost-effectiveness of oral PrEP for MSM in Cotonou (largest city of Benin) and its suburbs, as well as for MSM in Benin.

Our study prioritised retrospectively evaluating the impact/cost-effectiveness of the 2020-21 demonstration project, which was a key objective of the demonstration project study protocol.

Our study also prioritised prospectively evaluating the impact/cost-effectiveness of PrEP scale-up for MSM in Cotonou, to inform HIV prevention interventions for MSM used by the Beninese health authorities. We chose to focus on oral PrEP regimens and offering strategies that could be implemented in practice in Cotonou.

6. How will research products be shared in the community of study?

For instance, will you be providing written or oral layperson summaries for non-academic information sharing? Will study data be made available to institutions in the region(s) of study? The Lancet Global Health *encourages authors to translate the summary (abstract) into relevant languages after paper editing; do you intend to translate your summary?*

Preliminary results were disseminated in French at the final regional 3-day workshop of the research program POCAO (programme de recherche interventionnelle sur la santé sexuelle et l'équité pour les Populations Clés en Afrique de l'Ouest) meeting in Cotonou in 2022.

This workshop gathered more than 50 researchers from Bénin, Burkina-Faso, Mali, Congo, England and Canada, as well as the participation of the Minister of Health and the Coordinator of the National AIDS Control Programme in Benin.

Several reports of the different activities have been published on the POCAO dedicated website. https://pocao.org/

The modelling and cost-effectiveness paper abstract will be translated into French after paper editing.

7. How were individuals, communities, and environments protected from harm?

a) *How did you ensure that sensitive patient data was handled safely and respectfully? Was there any potential for stigma or discrimination against participants arising from any of the procedures or outcomes of the study?*

All data for secondary analysis were anonymised, and are securely stored on university computers. We ensured that the language used in publication and dissemination was respectful.

b) *Might any of the tests be experienced as invasive or culturally insensitive?*

N/A (the study is a modelling analysis of impact and cost-effectiveness, for details of the demonstration project see: https://doi.org/10.1002/jia2.26130)

c) *How did you determine that work was sensitive to traditions, restrictions, and considerations of all cultural and religious groups in the study population?*

The demonstration project research team included members of Benin's MSM community networks (BeSYP and RESEAU SIDA BENIN), who were involved in the were involved in the conception and implementation of the community aspect of the original demonstration project study, adhering to the principles of participatory research.<br><br>We ensured that the language used in publication and dissemination was respectful and culturally appropriate. Otherwise, our study was based on the demonstration project designed with our country collaborators which were able to inform on these.

d) *Were biowaste and radioactive waste disposed of in accordance with local laws?*

N/A

e) *Were any structures built that would have impacted members of the community or the environment (such as handwashing facilities in a public space)? If so, how did you ensure that you had appropriate community buy-in?*

N/A (the study is a modelling analysis of impact and cost-effectiveness, for details of the demonstration project observational study see: https://doi.org/10.1002/jia2.26130)

f) *How might the study have impacted existing health-care resources (such as staff workloads, use of equipment that is typically employed elsewhere, or reallocation of public funds)?*

N/A (the study is a modelling analysis of impact and cost-effectiveness, for details of the demonstration project see: https://doi.org/10.1002/jia2.26130)

8. Finally, please provide the title (eg, Dr/Prof, Mr/Mrs/Ms/Mx), name, and email address of an author who can be contacted about this statement. This can be the corresponding author.

**Name:** Trystan Leng<br>**Email:** trystan.leng@imperial.ac.uk
